# Design, Synthesis and Antifungal Activity of Novel 1,4-Pentadiene-3-one Containing Quinazolinone

**DOI:** 10.3390/ijms24032599

**Published:** 2023-01-30

**Authors:** Ran Zhou, Wenliang Zhan, Chunmei Yuan, Tao Zhang, Piao Mao, Zhiling Sun, Yousan An, Wei Xue

**Affiliations:** National Key Laboratory of Green Pesticide, Key Laboratory of Green Pesticide and Agricultural Bioengineering, Ministry of Education, Center for R&D of Fine Chemicals of Guizhou University, Guiyang 550025, China

**Keywords:** 1,4-pentadiene-3-one, quinazolinone, antifungal activity, *Sclerotinia sclerotiorum*, *Phomopsis* sp.

## Abstract

Twenty 1,4-pentadiene-3-one derivatives containing quinazolinone (**W1**–**W20**) were designed and synthesized. The bioactivity test results showed that some compounds had antifungal activities in vitro. **W12** showed excellent bioactivity against *Sclerotinia sclerotiorum* (*S. sclerotiorum*) and *Phomopsis* sp., with EC_50_ values of 0.70 and 3.84 μg/mL, which are higher than those of the control drug azoxystrobin at 8.15 and 17.25 μg/mL. In vivo activity tests were carried out on oilseed rape and kiwifruit. The protective effect of **W12** on oilseed rape infected with *S. sclerotiorum* (91.7 and 87.3%) was better than that of azoxystrobin (90.2 and 79.8%) at 100 and 50 μg/mL, respectively, and the protective effect on kiwifruit infected with *Phomopsis* sp. (96.2%) was better than that of azoxystrobin (94.6%) at 200 μg/mL. Scanning electron microscopy results showed the hyphae of *S. sclerotiorum* treated with compound **W12** abnormally collapsed and shriveled, inhibiting the growth of mycelium and, thus, laying the inhibiting effect on *S. sclerotiorum*. The results of the mechanism research showed that the action of **W12** changed the mycelial morphology of *S. sclerotiorum*, affected the permeability of cells, increased the leakage of cytoplasm and allowed the cell membrane to break down. This study shows that 1,4-pentadiene-3-one derivatives containing quinazolinone have good effects on plant fungi and the potential for becoming new fungicides.

## 1. Introduction

Plant pathogens such as *S. sclerotiorum*, *Phomopsis* sp. and *Rhizoctonia solani* affect agriculture seriously and reduce crop yield and quality greatly [1,2]. *S. sclerotiorum* is a necrotic homologous fungus, which belongs to Ascomycetes [3]. Oilseed rape is one of the important oil crops. *S. sclerotiorum* is the most serious disease of oilseed rape, which has threatened the safety of its production in the world deeply, leading to a decrease of more than 80% in the worldwide oilseed rape yield [4,5,6]. Kiwifruit is a very popular fruit, it has a unique taste and high nutritional value. *Phomopsis* sp. is a common plant fungus on kiwifruit, and mainly can cause fruit corruption, leaf bark necrosis and other diseases, affecting the fruit quality and economic benefits of kiwifruit [7]. At present, the use of a large number of fungicides is the main measure to control plant diseases. However, the use of a large number of fungicides has aggravated drug resistance of plant fungi, lessening the existing fungicides’ effect on plant pathogens. On the other hand, the widespread use of fungicides has caused serious environmental pollution [8,9,10]. Therefore, it is urgent to develop new green antifungal compounds with high efficiency and low toxicity.

**Figure 1:**
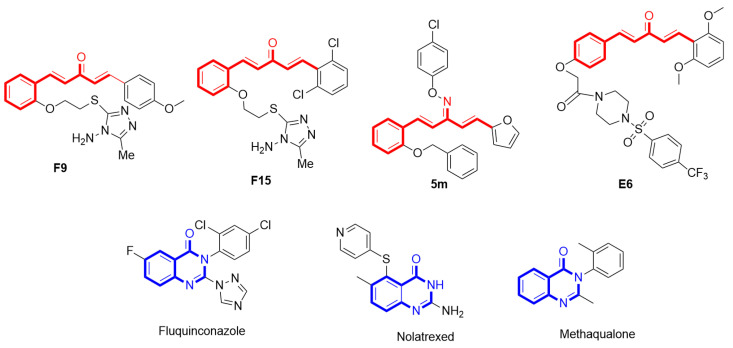
Reported active structures containing 1,4-pentadiene-3-one and quinazolinone fragments.

At present, drug molecular design and development based on the structure of natural products has become one of the hot spots of drug research. 1,4-pentadiene-3-one is the precursor compound of the biosynthesis of natural flavonoids [11]. The previous research work of our research group found that 1,4-pentadiene-3-one and its derivatives have a wide range of agricultural activities [12], such as antiviral [13], antibacterial [14], antifungal [15] and so on. For example, Chen et al. designed and synthesized a series of 1,4-pentadien-3-one derivatives containing 1,2,4-triazole, which showed great antibacterial and antiviral activities. Among them, compound **F9** showed good antibacterial activity against *Xanthomonas axonopodis pv. citri (Xac)* in vitro, with an EC_50_ of 5.4 μg/mL, which was better than that of the commercial agent bismerthiazol (54.9 μg/mL). Compound **F15** showed better protective activity against tobacco mosaic virus (TMV), with an EC_50_ of 105.4 μg/mL, which was better than that of ningnanmycin 214.7 μg/mL [16]. Tang et al. synthesized a series of 1,4-pentadiene-3-ketone derivatives containing oxime ether, among which compound **5m** had a strong passivating effect on TMV, which was 87.0%, better than that of ribavirin (77.9%) [17]. Zhou et al. introduced sulfonyl piperazine into 1,4-pentadiene-3-one and verified their antiviral, antibacterial and antifungal activities. Among them, the EC_50_ of compound **E6** (Figure 1) against *Phytophthora litchii* was 0.5 μg/mL, which was close to that of azoxystrobin (0.3 μg/mL) [18].

Quinazolinone exists in a variety of natural products and pharmaceutical molecules widely, with a wide range of pharmaceutical and biological activities, existing in a variety of natural alkaloids [19,20]. As an important pharmacophore of the antibacterial fluquinconazole, antineoplastic drug nolatrexed, sedative hypnone and other drugs [21], it has good antibacterial [22], anti-cancer [23], anti-inflammatory [24], hypotensive [25] and other activities, and has become a research hotspot in recent years [26].

**Figure 2:**
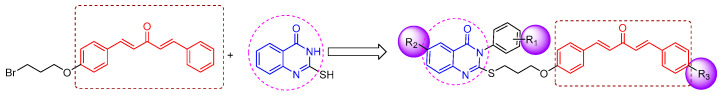
Design of the target compounds.

On the basis of these considerations, we have made a reasonable structural modification in 1,4-pentadiene-3-one, and a series of 1,4-pentadiene-3-one compounds containing quinazolinone were synthesized (Figure 2). Through biological activity screening and mechanism research, we screened out the compound **W12**, which had significantly better biological activity than azoxystrobin.

## 2. Result and Discussion

### 2.1. Chemistry

**Scheme 1:**
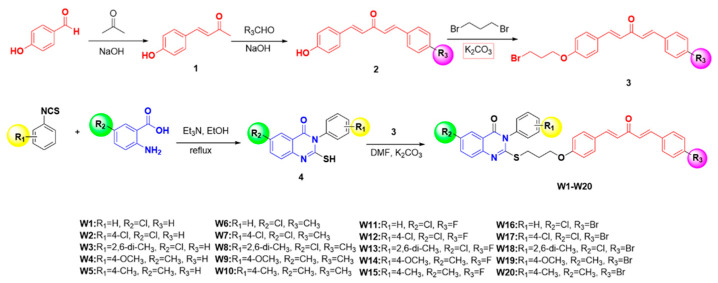
Schematic representation of the synthesis of target compounds **W1**–**W20**.

The synthetic route of title compounds **W1**–**W20** are shown in Scheme 1. According to the synthesis method in reference [27,28], the intermediate **1** was synthesized from hydroxybenzaldehyde and acetone in an ice bath, substituted benzaldehydes reacted with intermediate **1** to synthesize intermediate **2**, 1,3-dibromopropane reacted with intermediate **2** to synthesize intermediate **3** under heating conditions and intermediate **4** was synthesized from substituted phenyl isothiocyanate and substituted *o*-aminobenzoic acid. Intermediates **4** and **3** were stirred at room temperature for 4 h with K_2_CO_3_ as an acid binding agent and DMF as a solvent to finally synthesize quinazolinone containing 1,4-pentadiene-3-one derivatives **W1**–**W20**, and their structures were confirmed by ^1^H NMR, ^13^C NMR, ^19^F NMR and HRMS data and listed in the Supplementary Materials.

### 2.2. In Vitro Antifungal Bioassay

First of all, we determined the antifungal activity of 1,4-pentadiene-3-one derivatives containing quinazolinone at a concentration of 100 μg/mL against 10 important plant fungi. It can be seen from Table 1 that **W12** showed an excellent inhibition rate against *S. sclerotiorum* (100%), *Phomopsis* sp. (100%) and *R. solani* (100%) at this concentration, which is superior to that of azoxystrobin (56.6, 75.9 and 41.2%) at 100 μg/mL. It showed good activity against *P. capsica*, *B. dothidea* and *F. asiaticum* (91.1, 84.9 and 80.2%), and its inhibitory activity was better than that of the control drug azoxystrobin (44.1, 56.3 and 75.8%). The inhibition rate of **W17** against *S. sclerotiorum* (100%) and *Phomopsis* sp. (100%) at this concentration was superior to that of the control drug azoxystrobin. The antibacterial activity against *R. solani* was 79.1%, which was better than that of the control drug. At the same time, the inhibition rates of **W18** and **W19** against *S. sclerotiorum* in oilseed rape reached 100% at this concentration.

The results in Table 1 show that 1,4-pentadiene-3-one derivatives containing quinazolinone have obvious antifungal activities. We studied the substituents on the benzene ring in the structure and analyzed the structure–activity relationship. First, when R_3_ was substituted with -F and -Br, the antifungal activity of compounds was better than that of compounds substituted by -H and -CH_3_, for example, **W11**(-F) > **W16**(-Br) > **W6**(-CH_3_) > **W1**(-H). This shows that when the substituent phenyl group on the 1,4-pentadiene-3-one structure is replaced by an electron withdrawing group, the compound has better antifungal activity, which positively correlates with the polarity of the electron withdrawing group. The greater the polarity is, the greater the antifungal activity is. Based on these, we found that when R_1_ = 4-Cl and R_2_ = Cl were present, they showed better antifungal activity than in the condition where R_1_ = H, 2,6-di-CH_3_, 4-OCH_3_ and R_2_ = CH_3_ were.

**Figure 3:**
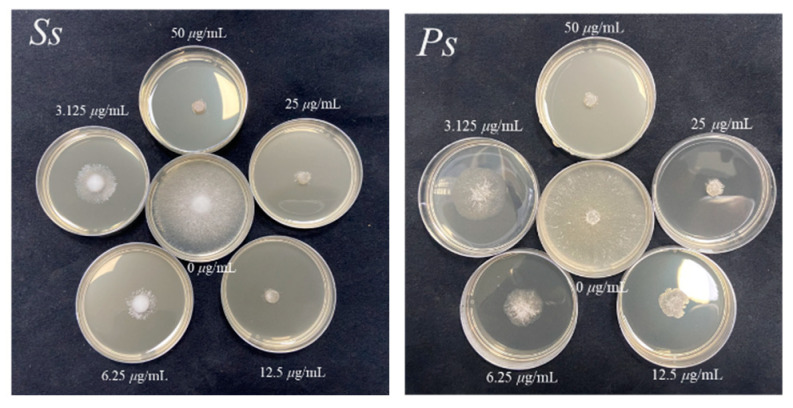
Antifungal activities of **W12** against *S. sclerotiorum* (*Ss*) and *Phomopsis* sp. (*Ps*) in vitro.

In summary, when the substituent group on the benzene ring of the compound is an electron withdrawing group, it is beneficial to increase the antibacterial activity, based on the results of fungal bioactivity test in vitro. In order to further verify the antifungal potential of this compounds, we measured the half maximal effective concentration (EC_50_) of some compounds with good activity (Table 2, Figure 3). It is noteworthy that the EC_50_ value of W12 against plant pathogens ranges from 0.70–14.04 μg/mL, which is higher than that of the control drug azoxystrobin 8.51–22.25 μg/mL. It can be seen that **W12** has good broad-spectrum antifungal activity. Therefore, a series of derivatives have been synthesized by introducing the active group quinazolinone into the 1,4-pentadiene-3-one structure, having good antifungal activity and the potential for being fungicides.

### 2.3. In Vivo Fungicidal Activities Resist S. sclerotiorum

**Figure 4:**
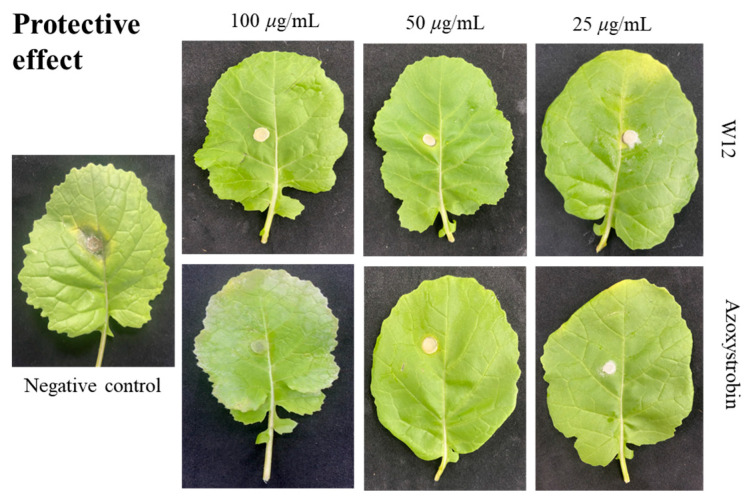

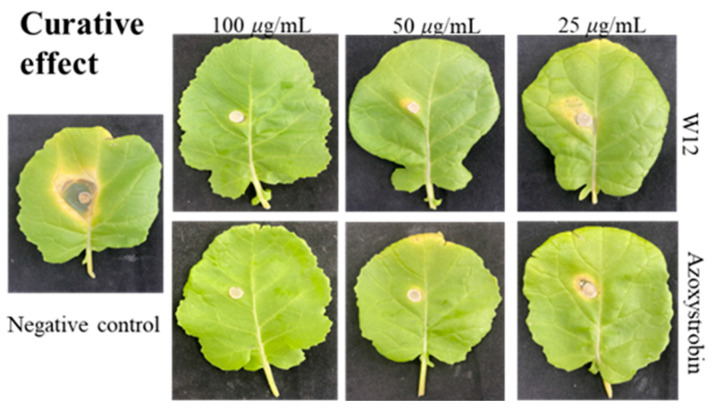
The curative effect and protective effect of **W12** against *S*. *sclerotiorum*.

The results of in vivo experiments showed that **W12** has good protective and curative effects (Figure 4, Table 3) on oilseed rape leaves infected with *S. sclerotiorum*. First, **W12** shows a strong protective effect (91.7, 87.3 and 72.3%) at 100, 50 and 25 μg/mL, and it also shows a good curative effect (87.6, 78.8 and 50.2%). Second, the protective effect (91.7 and 87.3%) was better than that of the positive control drug azoxystrobin (90.2 and 79.8%). The curative effect at 100 μg/mL (87.6%) was equivalent to that of azoxystrobin (88.2%). Therefore, we can draw the following conclusion, that **W12** has good curative and protective activities in vivo. As shown in the picture of Figure 3, **W12** shows no obvious toxicity to oilseed rape leaves at high concentration.

### 2.4. In Vivo Fungicidal Activities Resist Phomopsis sp.

**Figure 5:**
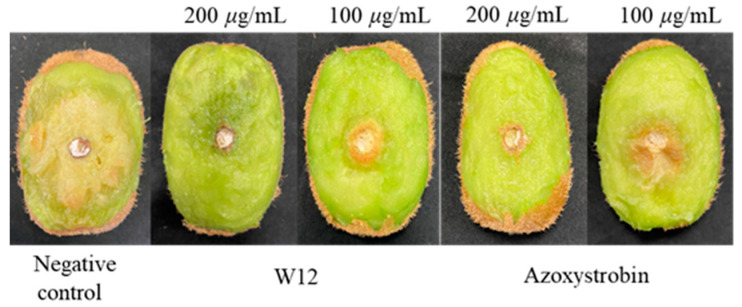
Protective activity of **W12** and azoxystrobin against *Phomopsis* sp. on kiwifruit.

The effects of **W12** on the postharvest of kiwifruit are demonstrated in Figure 5. As can be seen from Table 4, at 200 and 100 μg/mL **W12** has an obvious protective effect on kiwifruit and can significantly reduce the diameter of pathological changes and inhibit the pathological process. **W12** shows a good protective effect (96.2 and 86.4%) that is better than that of azoxystrobin (94.6 and 80.0%). Therefore, we can draw a conclusion that **W12** has a good protective activity in vivo.

### 2.5. Scanning Electron Microscopy (SEM) of Compound ***W12*** on the Hyphae Morphology

**Figure 6:**
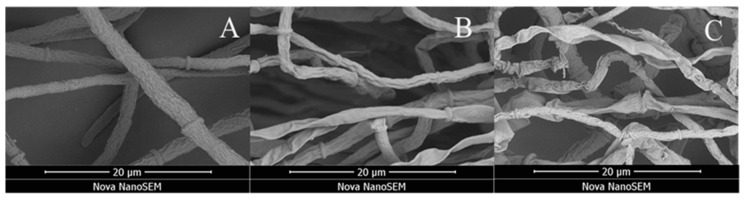
SEM images of the hyphae of *S. sclerotiorum* after treatment with different concentrations of **W12**. (**A**) 0 µg/mL, (**B**) 50 µg/mL, and (**C**) 100 µg/mL. Scale bar for (**A**–**C**) are 20 µm.

The SEM results of compound **W12** are shown in Figure 6. The mechanism of **W12** on *S. sclerotiorum* was further studied by using SEM, which is consistent with the previous experimental conclusions. The mycelia morphology of *S. sclerotiorum* treated with compound **W12** changed significantly. The mycelium of the group without being treated with the drug were complete and plump. However, when the concentration of **W12** was 50 μg/mL, curly folds and uneven surfaces appeared in the mycelium. When the drug concentration increased to 100 μg/mL, the mycelium folds became more obvious and shrunk. It is concluded that with the increase of drug concentration, the degree of damage to the mycelium is more obvious, indicating that **W12** can destroy its mycelium morphology, lower the growth of mycelium and, thus, lay the inhibiting effect on *S. sclerotiorum*.

### 2.6. Effect of ***W12*** on the Cell Membrane Permeability of S. sclerotiorum

**Figure 7:**
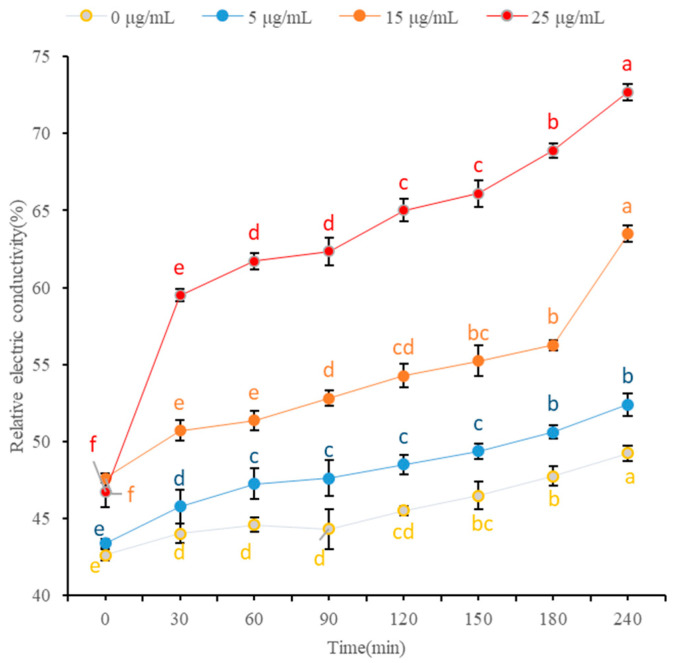
Changes in cell membrane permeability of **W12** against *S. sclerotiorum* (Different letters indicate significant differences at *p* < 0.05 in the same group).

As shown in Figure 7, compared with the blank control group, the relative conductivity of the mycelium increased with time after being treated with **W12**. The conductivity also increased with the increase of drug concentration; the rising trend was greater than that of the control group. The results showed that **W12** had a significant effect on the permeability of the mycelium cell membrane, which increased its permeability and, thus, laid inhibition. The final data were analyzed using SPSS v.25.0 (IBM, New York, NY, USA). Different letters indicate significant differences at *p* < 0.05 in the same group.

### 2.7. Effect of ***W12*** on the Cytoplasmic Leakage of S. sclerotiorum

**Figure 8:**
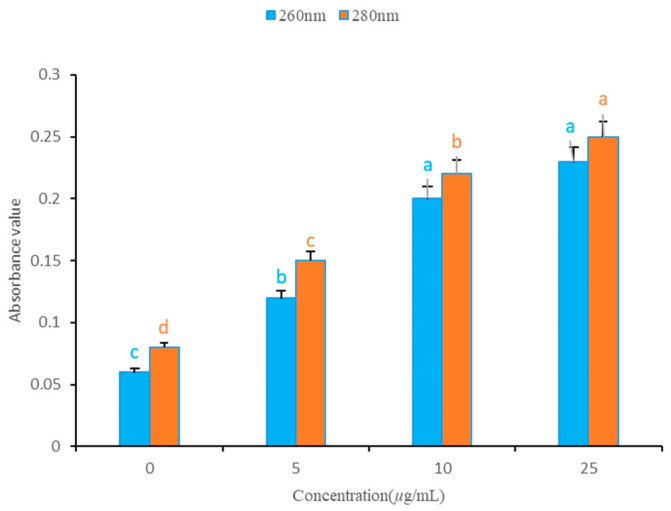
Release of cellular contents from *S. sclerotiorum* after treatment with **W12** (Different letters indicate significant differences at *p* < 0.05 in the same group).

In Figure 8, an ultraviolet spectrophotometer was used to measure the absorbance of mycelia clear solution at 260 nm and 280 nm and analyze the release degree of mycelium cytoplasm. It can be seen from the figure that the absorbance of the mycelium clear solution was in direct proportion to the concentration when being treated with **W12** at 260 nm and 280 nm, so the following conclusion can be drawn: the leakage of the cytoplasm content of the mycelium treated with **W12** was increasing, and its release degree was also increasing. The final data were analyzed using SPSS v.25.0 (IBM, New York, NY, USA). Different letters indicate significant differences at *p* < 0.05 in the same group.

### 2.8. Effect of ***W12*** on the Cytoplasmic Leakage of S. sclerotiorum

**Figure 9:**
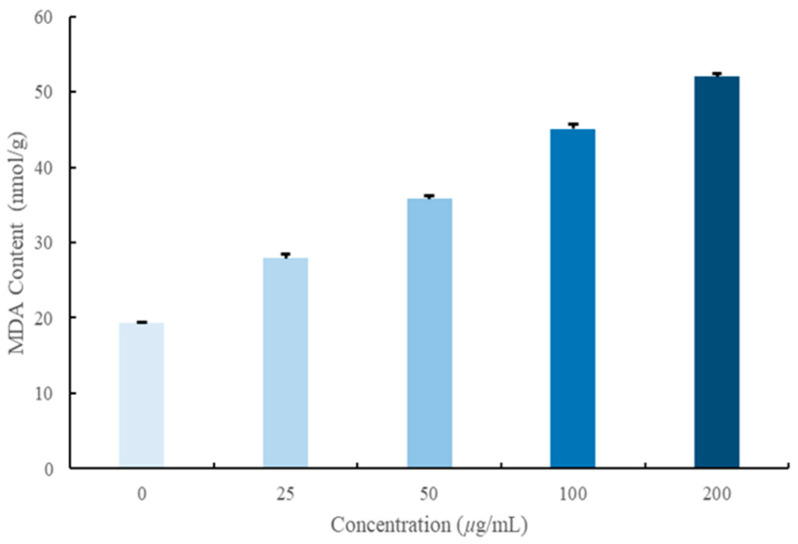
MDA content of *S. sclerotiorum* after treatment with **W12**.

The content of malondialdehyde (MDA) can indicate the degree of lipid peroxidation. The higher the content of malondialdehyde is, the more serious the damage to the cell membrane is. Figure 9 shows the content of MDA in *S. sclerotiorum* mycelium treated with different concentrations of **W12** (0, 25, 50, 100 and 200 μg/mL). It can be seen from the figure that the content of MDA gradually increases with the increase of the concentration, higher than the control group significantly. It can be concluded that **W12** can cause cell membrane damage, and the degree of damage increases with the increase of concentration.

### 2.9. Molecular Docking

**Figure 10:**
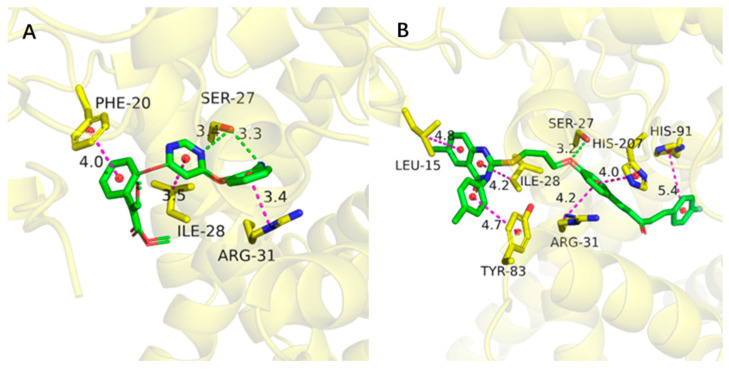
Binding models of the compounds azoxystrobin (**A**) and **W12** (**B**) with SDH protein (PDB ID: 2WDQ). The purple line indicates hydrophobic interactions between compounds and surrounding residues. The green line indicates hydrophilic hydrogen bond interactions.

We studied the action mode of compound **W12** on the associated protein Succinate dehydrogenase (SDH) of *S. sclerotiorum* by a molecular docking experiment. As shown in Figure 10, the positive control azoxystrobin mainly forms hydrophobic interactions with the amino acids around the active pocket, such as the π–π stacking formed by the benzene ring and Phe20 (4.0 Å), the π–Alkyl stacking formed by the pyrimidine ring and Ile28 (3.5 Å), the π–Alkyl stacking formed by another benzene ring and Arg31 (3.4 Å) and the hydrogen bonding formed by Ser27 with the bond lengths of 3.4 Å and 3.3 Å, respectively. The interaction between **W12** and the amino acids around the active pocket is mainly hydrophobic, which is similar to the positive control azoxystrobin. This is due to the fact that the compound contains more aromatic ring groups, such as Leu15 (4.8 Å), Ile28 (4.2 Å) and Tyr83 (4.7 Å), which form hydrophobic interactions with N and quinazolinone at the head, Arg31 and His207 form π–π stacking and π–Alkyl stacking with the middle benzene ring and the benzene ring at the tail forms π–π stacking and other hydrophobic interactions with His91. At the same time, similar to azoxystrobin, **W12** forms a hydrogen bond with Ser27, and the bond length is 3.2 Å. In conclusion, **W12** introduced more aromatic groups, resulting in a larger molecular volume of the compound, which formed more hydrophobic interactions with protein than azoxystrobin and, finally, made the **W12** have higher antibacterial activity.

## 3. Materials and Methods

### 3.1. Instruments and Chemicals

The melting point was measured on an XT-4 binocular microscope (Beijing Taike Instruments Co., Ltd., Beijing, China), without calibration. ^1^H, ^13^C and ^19^F were obtained by using a 500 MHz nuclear magnetic resonance (NMR) instrument (JEOL-ECX500, Japan Electronics Co., Tokyo, Japan). High-resolution mass spectra (HRMS) were obtained by using a Thermo Scientific Q Exactive Hybrid Quadrupole Mass Spectrometer (Thermo Scientific, St. Louis, MO, USA). Scanning electron microscopy (SEM) data were obtained on FEI Nova Nano 450 (FEI Co., Hillsboro, OR, USA). The cell permeability was measured on the conductivity meter, Leici DDSJ-3O8F (Shanghai Instrument & Electric Science Instrument Co., Ltd., Shanghai, China), and the cell leakage was recorded on the N-5000 ultraviolet spectrophotometer (Shanghai Yoke Instrument Co., Ltd., Shanghai, China). The reagents and solvents used in the experiment were purchased from Shanghai Titan Chemical Co., Ltd. (Shanghai, China), Beijing Solarbio Technology Co., Ltd. (Beijing, China) and Tianjin Zhiyuan Chemical Reagent Co., Ltd. (Tianjin, China). All reagents and solvents used were analytical grade, and they were directly used without further purification or drying.

#### 3.1.1. General Procedure for the Synthesis of Intermediate **1**

P-hydroxybenzaldehyde (20.6 mmol) was added to 60 mL of acetone and stirred for about 15 min. After the reaction system was bathed in ice for about 30 min, about 100 mL of 5% NaOH solution was added to the system. After the dripping was finished, the ice bath was removed and stirred for about 24 h at room temperature and monitored by TLC (petroleum ether: ethyl acetate =2:1). After the reaction, the system was transferred to a 500 mL beaker and an appropriate amount of ice water was added. After the pH of the system was adjusted to about 5–6 with 5% dilute hydrochloric acid solution, a large number of yellow solids were precipitated and intermediate **1** was obtained

#### 3.1.2. General Procedure for the Synthesis of Intermediates **2** and **3**

Intermediate **1** (30.8 mmol), aromatic formaldehyde (39.6 mmol) and ethanol (40 mL) were stirred until dissolved. Under the condition of an ice bath, 40 mL of about 5% sodium hydroxide solution was slowly added, and black-red turbid solution was obtained after stirring for 24 h at room temperature. Under the ice bath, 5% dilute hydrochloric acid was used to adjust the pH to about 5–6, and a yellow precipitate was generated. After standing, intermediate **2** was obtained by extraction, filtration and water washing, and intermediate **2** (20.4 mmol) was added to 50 mL acetonitrile for 1 h. After the reaction, dibromoethane (67.1 mmol) was added. The reaction was carried out at 80 ℃ for 6 h, followed by TLC (petroleum ether: ethyl acetate =3:1). After the reaction, the reaction was extracted with ethyl acetate, the substrate was dried with anhydrous Na_2_SO_4_ and the key intermediate **3** was distilled under reduced pressure.

#### 3.1.3. General Procedure for the Synthesis of Intermediate **4**

Substituted anthranilic acids (17.5 mmol) and 40 mL EtOH were added to a 100 mL single-neck round bottom flask, then substituted phenyl isothiocyanate (13.23 mmol) and EtN_3_ (14.55 mmol) were added, heated and stirred until reflux. The reaction was monitored by TLC (petroleum ether: ethyl acetate = 2:1) at this temperature to continue the reaction for 4–6 h. There was white solid precipitation. After the reaction was over, the heating device was turned off, the product was cooled to room temperature and vacuum filtration was used to obtain the precipitation product, which was washed with a small amount of ethanol to obtain intermediate **4**. The yield was 79.0–90.0%.

#### 3.1.4. General Procedure for the Synthesis of Target Compounds **W1**–**W20**

Intermediate **3** (1.25 mmol) was added to 20 mL of DMF, then intermediate **4** (1.25 mmol) and K_2_CO_3_ (2.5 mmol) were added successively and the reaction was monitored by TLC (petroleum ether: ethyl acetate = 2:1) for 6 h at room temperature. At the end of the reaction, the reaction system was poured into 100 mL ice water and yellow solid was precipitated. After standing for 2 h, the crude product was extracted and filtered under reduced pressure. After column chromatography separation and purification (ethyl acetate: petroleum ether = 5:1), the target compounds **W1–W20** were obtained, and the yield was 29–83%.

### 3.2. Antifungal Activity Bioassay In Vitro

*B. dothidea*, *R. solani*, *B. cinerea*, *S. sclerotiorum*, *Phomopsis* sp. and *P. capsici* were provided by the School of Food and Pharmaceutical Engineering, Guiyang University, and *F. asiaticum, F. equiseti*, *N. dimidiatum* and *C. gloeosporioides* were provided by the College of Sciences, Guizhou University. In vitro antifungal activity was determined with the mycelial growth rate method [29,30]. *Rhizoctonia solani (R. solani)*, *Botrytis cinerea (B. cinerea)*, *Sclerotinia sclerotiorum (S. sclerotiorum)*, *Botryosphaeria dothidea (B. dothidea), Phytophthora capsici (P. capsici), Fusarium asiaticum (F. asiaticum), Fusarium equiseti (F. equiseti)*, *Neoscytalidium dimidiatum (N. dimidiatum)*, *Colletotrichum gloeosporioides (C. gloeosporioides)* and *Phomopsis* sp. were selected for the experiment. First, potato dextrose agar (PDA) was sterilized for 20 min at 121 °C, then the compound was dissolved in dimethyl sulfoxide (DMSO) and mixed with PDA to obtain final concentrations of 100 μg/mL. The mixture was then poured into a 6 cm petri dish, with each group having 3 replicates. A solution of 0.5% DMSO was used as the blank control and 100 μg/mL azoxystrobin was used as the positive control. After cooling, a 5 mm agar plate containing mycelium was inoculated in the center of the culture dish with a sterile inoculation needle. Finally, when the culture dishes of the blank control group were completely occupied by fungi, the mycelial growth diameter was measured and the following formula was used to calculate:

I(%)=(C−T)/(C−5mm)×100

where *I* is the inhibition rate and C and T represent the average growth diameter of fungi on PDA of the blank control group and the drug treatment group, respectively.

The compounds with better activity were selected to further determine their half maximal effective concentrations (EC_50_) according to the same method mentioned above, and the inhibition rates of the target compound at 100, 50, 25, 12.5 and 6.125 µg/mL were determined. The logarithm of concentration 10 was taken as the abscissa and the corresponding value of inhibition rate was taken as the ordinate. The corresponding value of EC50 can then be found, and each experiment was repeated three times.

### 3.3. Antifungal Bioassay In Vivo

In vivo antifungal experiments were carried out on the basis of in vitro antifungal activity test results. The control effect of **W12** on *S. sclerotiorum* and *Phomopsis* sp. of oilseed rape was determined by using the oilseed rape variety “Taifei No. 1” and the kiwifruit variety “Miliang No. 1” as experimental materials, and the commercial fungicide azoxystrobin as a positive control. For the curative and protective activity of **W12** on oilseed rape, the leaves of oilseed rape with uniform size were selected, which were sterilized with 1% sodium hypochlorite for 1 min. After washing with sterile water, the surface water was dried with filter paper. To evaluate the protective activity, **W12** (100, 50 and 25 μg/mL) was uniformly sprayed on the surface of the leaves, azoxystrobin and sterile distilled water were used as a positive control and negative control, respectively, and the negative control group was prepared without compounds. After 24 h in the incubator, 5 mm agar disks containing mycelium were placed in the middle of the oilseed rape leaves. To evaluate the curative activity, the agar disk containing mycelium was inoculated on the leaves for 24 h and then sprayed on the leaf surface with different concentrations of **W12**. For the n vivo antifungal experiment of kiwifruit, it was necessary to drill a 5 mm hole with a hole punch at the equator of the kiwifruit, and its curative and protective activity was the same as the procedure of the appeal experiment. After inoculation, the experimental materials were incubated in an incubator at 25 °C and 85% relative humidity for 72 h, and the diameter of the plaque was measured. The control effect was calculated as follows:

*C* (%) = [(*A_CK_* − *A*_1_)/(*A_CK_* − 5)] × 100

*C* represents the control effect (%);

*A_CK_* represents the lesion diameter of the blank control group;

*A*_1_ represents the lesion diameter after being compound treated.

There were three parallel sets for each concentration, and the experiments were repeated at least twice [31,32,33].

### 3.4. Scanning Electron Microscope (SEM) Observation

In order to evaluate the effect of **W12** on mycelium, the morphology of mycelia was observed on a scanning electron microscope. **W12** (100 and 50 μg/mL) was used to treat the mycelium and prepare the sample according to the literature method [34]. A mycelial disk with 5 mm diameter was taken from the periphery of a colony grown on PDA medium containing 100 and 50 μg/mL of compound **W12** and incubated for 48 h at 28 °C. The youngest *S. sclerotiorum* hyphae were chosen from the margin of the mycelia, and the samples were routinely fixed in 2.5% glutaraldehyde at 4 °C overnight, briefly post-fixed for 3 h at 4 °C in tert-butanol and then dehydrated in a graded ethanol series, critical point dried and gold coated. The samples were imaged using the scanning electron microscope FEI Nova Nano SEM 450.

### 3.5. Determination of Cell Membrane Permeability of ***W12*** to S. sclerotiorum

According to previous reports, the permeability of the mycelium cell membrane was analyzed by measuring the relative electrical conductivity of mycelium suspension. A total of 4 (5 mm) *S. sclerotiorum* agar disks containing mycelium were placed into 100 mL PD medium and shaken at 25 °C at 180 rpm for 72 h. An appropriate amount of mycelium was taken, filtered and washed with sterile water three times. The mycelium was weighed with dry weight of 200 mg, and the mycelium was treated with **W12** (25, 10 and 5 μg/mL). The conductivity detector was used to obtain the data [35,36].

### 3.6. Determination of Cytoplasmic Leakage of ***W12*** to S. sclerotiorum

The release degree of mycelia cytoplasm was analyzed by measuring the leakage amount of cytoplasm contents. Mycelia were treated with different concentrations of **W12** (25, 10 and 5 μg/mL), and an ultraviolet spectrophotometer was used to measure the absorbance value of the clear solution after removing the mycelium at the wavelengths of 260 nm and 280 nm [37].

### 3.7. Determination of Malondialdehyde Content

Malondialdehyde is one of the main products of lipid peroxidation. Its content can indicate the degree of lipid peroxidation and can be used to judge the degree of cell membrane damage [38]. *S. sclerotiorum* was incubated in 50 mL of PD medium at 25 °C for 48 h at 180 rpm, then incubated in **W12** containing different concentrations (0, 25, 50, 100 and 200 μg/mL) for 1 d. The medium was washed with sterile water. After filtration, the mycelium were collected and freeze-dried for 5 h. A total of 0.1 g of mycelium was weighed, and 1 mL of the extract was added (Beijing Solarbio Technology Co., Ltd.) under the condition of an ice bath, 8000 rpm, 4 °C. It was centrifuged for 10 min, the supernatant was taken and each reagent was added according to the instructions. Next, the mixture was placed in a 100 °C water bath for 60 min, then placed in an ice bath for cooling. Then, the mixture was centrifuged at 10,000 rpm, 25 °C, for 10 min, 200 μL of the supernatant was sucked and placed into 96-well plates and the absorbance of each sample was measured at 532 nm and 600 nm.

### 3.8. Molecular Docking

The crystal structure of SDH protein (PDB ID: 2WDQ) was acquired from the RCSB Protein Data Bank and further treated with the addition of hydrogen atoms and the removal of water molecules with PYMOL. To prepare and optimize the compound structures, ChemOffice 2019 was used and the binding mode of the compounds to SDH proteins was determined by the program AutoDock vina 1.1.2. The conformations of azoxystrobin and **W12** were then clustered using root mean square deviation (RMSD) = 2 Å as the threshold value [39].

## 4. Conclusions

In this work, twenty 1,4-pentadiene-3-one derivatives containing quinazolinone (W1–W20) were designed and synthesized, and their biological activity tests were carried out. The results of the antifungal experiment in vitro showed that W12, W17 and W18 had good inhibitory activity. The EC_50_ value of W12 against *S. sclerotium* was 0.70 μg/mL. In addition, the in vivo activity experiment results showed that the protective and curative effect of W12 (91.7 and 87.6%) on oilseed rape was better than that of azoxystrobin (90.2 and 88.2%) at 100 μg/mL. The results of SEM also confirmed that W12 can cause the hyphae to curl and fold, the hyphae surface to be uneven and the hyphae structure to be damaged. The effects of W12 on cell membrane permeability and cytoplasmic leakage of *S. sclerotiorum* was consistent with the previous experimental results. It could also lead to an increase in malondialdehyde levels, which could cause cell membrane damage. The results of molecular docking experiments also show that W12 has more hydrophobic interactions with protein than azoxystrobin, which made W12 have higher antifungal activity. In view of this, we can assert that W12 shows excellent antifungal activity, which also confirms that the derivatives of 1,4-pentadiene-3-one containing quinazolinone can be further developed as potential fungicides and also provides a new idea and some theoretical basis for the creation of green pesticides.

## Figures and Tables

**Table 1:** In vitro antifungal activities of the title compounds **W1**–**W20** at 100 μg/mL.

Compounds	Inhibition Rate (%) *^a^*									
	*Bd* *^b^*	*Rs*	*Bc*	*Ss*	*Ps*	*Pc*	*Fa*	*Fe*	*Nd*	*Cg*
**W1**	2.8 ± 1.1	19.2 ± 1.7	3.1 ± 0.8	17.4 ± 1.1	14.9 ± 1.1	45.7 ± 0.9	15.8 ± 1.2	12.1 ± 2.6	11.2 ± 1.1	9.6 ± 3.2
**W2**	6.1 ± 4.3	29.2 ± 1.7	3.9 ± 1.6	16.6 ± 0.8	15.3 ± 0.9	35.1 ± 3.1	24.2 ± 1.1	13.3 ± 1.2	14.6 ± 1.6	11.2 ± 2.3
**W3**	5.6 ± 0.1	33.3 ± 4.9	4.6 ± 0.8	15.5 ± 1.6	9.2 ± 2.3	44.9 ± 1.2	18.3 ± 1.1	12.1 ± 1.7	7.0 ± 1.6	17.1 ± 1.6
**W4**	8.5 ± 1.1	24.3 ± 1.6	3.5 ± 2.1	7.9 ± 1.1	12.5 ± 4.1	33.1 ± 1.1	10.0 ± 2.5	10.4 ± 1.7	12.1 ± 1.6	5.0 ± 1.9
**W5**	2.4 ± 0.1	23.5 ± 8.2	5.8 ± 2.2	9.4 ± 0.2	10.8 ± 1.9	30.6 ± 1.4	13.3 ± 1.1	15.8 ± 1.8	15.0 ± 1.4	7.1 ± 1.6
**W6**	2.9 ± 1.2	19.2 ± 1.7	5.0 ± 0.1	14.3 ± 0.8	2.4 ± 1.2	34.3 ± 1.7	10.0 ± 2.0	10.4 ± 1.7	22.5 ± 1.9	17.5 ± 1.9
**W7**	2.2 ± 0.4	25.4 ± 1.1	6.2 ± 1.1	9.1 ± 0.8	8.4 ± 1.5	26.9 ± 3.3	9.2 ± 1.2	13.7 ± 1.9	12.9 ± 2.5	5.8 ± 3.5
**W8**	2.1 ± 0.5	21.5 ± 1.7	4.3 ± 1.0	9.4 ± 0.1	8.3 ± 1.9	27.4 ± 1.1	13.7 ± 1.2	10.8 ± 1.2	7.9 ± 1.6	2.1 ± 1.6
**W9**	4.8 ± 2.2	19.2 ± 2.2	6.2 ± 2.2	8.3 ± 1.1	6.4 ± 1.6	30.6 ± 2.7	5.0 ± 1.4	3.7 ± 1.2	25.8 ± 1.1	3.7 ± 1.8
**W10**	2.5 ± 0.2	18.0 ± 0.9	5.7 ± 1.7	18.3 ± 1.1	5.2 ± 1.8	31.0 ± 3.3	9.2 ± 1.8	7.9 ± 2.6	18.7 ± 1.8	25.4 ± 1.6
**W11**	32.4 ± 0.3	22.2 ± 1.1	33.9 ± 1.8	47.2 ± 0.3	26.4 ± 0.9	35.5 ± 1.1	27.5 ± 1.4	15.8 ± 2.3	10.0 ± 1.9	20.8 ± 1.1
**W12**	84.9 ± 1.7	100	50.2 ± 1.1	100	100	91.1 ± 1.6	68.7 ± 1.2	80.2 ± 2.0	77.1 ± 1.6	57.5 ± 1.9
**W13**	23.7 ± 1.1	18.8 ± 1.2	46.9 ± 0.9	52.8 ± 1.1	33.3 ± 1.4	43.9 ± 1.4	45.4 ± 1.7	20.4 ± 1.7	37.5 ± 1.4	36.2 ± 1.2
**W14**	2.6 ± 1.1	16.5 ± 1.1	6.2 ± 1.8	15.9 ± 0.8	4.4 ± 1.1	26.5 ± 2.4	21.6 ± 1.2	15.0 ± 2.0	5.8 ± 1.7	14.2 ± 1.8
**W15**	3.9 ± 0.5	9.8 ± 1.1	3.1 ± 0.8	17.7 ± 1.1	27.7 ± 0.1	32.2 ± 2.3	17.5 ± 1.4	12.5 ± 1.4	23.3 ± 1.7	5.8 ± 1.8
**W16**	13.2 ± 1.2	16.1 ± 1.1	6.2 ± 2.2	11.3 ± 2.0	17.3 ± 1.1	22.5 ± 2.2	9.6 ± 2.2	16.6 ± 1.1	29.6 ± 1.6	34.6 ± 1.6
**W17**	46.5 ± 0.5	79.1 ± 0.3	10.8 ± 1.1	100	100	49.1 ± 1.9	11.6 ± 1.8	58.7 ± 1.2	62.9 ± 1.6	42.1 ± 1.6
**W18**	32.8 ± 0.1	29.8 ± 0.8	8.1 ± 1.1	100	48.6 ± 2.3	43.7 ± 1.4	33.3 ± 1.1	25.4 ± 1.7	36.2 ± 1.2	43.8 ± 1.2
**W19**	4.9 ± 0.9	30.2 ± 1.1	5.8 ± 1.9	100	28.5 ± 1.1	35.1 ± 1.2	10.4 ± 1.7	24.2 ± 2.3	10.4 ± 2.5	12.1 ± 1.6
**W20**	6.1 ± 1.1	24.3 ± 1.6	11.9 ± 1.8	7.9 ± 1.1	44.9 ± 1.6	45.1 ± 1.7	14.6 ± 1.7	20.4 ± 1.7	8.7 ± 1.8	19.2 ± 1.1
**Azoxystrobin** *^c^*	56.3 ± 2.6	41.2 ± 1.9	61.7 ± 1.5	56.6 ± 1.6	75.9 ± 0.2	44.1 ± 2.6	51.2 ± 1.9	75.8 ± 1.1	93.3 ± 1.1	74.6 ± 1.6

*^a^* Values are mean ± SD of three replicates. *^b^* ***Bd*** (*B. dothidea*), ***Rs*** (*R. solani*), ***Bc*** (*B. cinerea*), ***Ss*** (*S. sclerotiorum*), ***Ps*** (*Phomopsis sp.*), ***Pc*** (*P. capsici*), ***Fa*** (*F. asiaticum*), ***Fe*** (*F. equiseti*), ***Nd*** (*N. dimidiatum*), ***Cg*** (*C. gloeosporioides*)*. ^c^* The commercial antifungal agent.

**Table 2:** EC_50_ values of several target compounds *^a b^*.

Compound	Phytopathogen	Regression Equation	EC_50_ (μg/mL)	r
**W12**	*Ss*	Y = 0.89X + 5.13	0.70 d	0.98
**W17**	*Ss*	Y = 0.87X + 3.93	16.22 b	0.97
**W18**	*Ss*	Y = 0.39X + 4.34	45.30 a	0.96
Azoxystrobin *^c^*	*Ss*	Y = 1.11X + 3.95	8.51 c	0.97
**W12**	*Ps*	Y = 0.96X + 3.91	3.84 c	0.95
**W17**	*Ps*	Y = 0.21X + 4.72	18.80 a	0.99
Azoxystrobin	*Ps*	Y = 0.61X + 4.24	17.25 b	0.97
**W12**	*Rs*	Y = 0.54X + 4.68	14.04 c	0.99
**W17**	*Rs*	Y = 0.36X + 4.47	27.82 a	0.99
Azoxystrobin	*Rs*	Y = 0.73X + 4.01	22.25 b	0.99

*^a^* Values are mean ± SD of three replicates. *^b^* Statistical analysis was conducted by SPSS 25.0 software. Different letters indicate significant differences at *p* < 0.05 in the same group. *^c^* The commercial antifungal agent.

**Table 3:** Curative and protective activities of **W12** against *S. sclerotiorum* in vivo ^*a b*^.

Compound	Concentration (μg/mL)	Curative Effect		Protective Effect	
		Lesion Length (mm ± SD)	Controlling Efficacy (%)	Lesion Length (mm ± SD)	Controlling Efficacy (%)
**W12**	100	7.5 ± 0.3 e	87.6	6.1 ± 0.6 d	91.7
	50	9.3 ± 0.6 d	78.8	6.7 ± 0.8 cd	87.3
	25	15.1 ± 0.4 b	50.2	8.7 ± 0.2 b	72.3
Azoxystrobin *^c^*	100	7.2 ± 0.2 e	88.2	6.3 ± 0.4 d	90.2
	50	7.9 ± 0.9 e	85.7	7.7 ± 0.3 bc	79.8
	25	10.5 ± 0.8 c	72.1	8.1 ± 0.5 b	76.8
Control	0	25.3 ± 0.2 a		18.4 ± 0.7 a	

*^a^* Values are mean ± SD of three replicates. *^b^* Statistical analysis was conducted by SPSS 25.0 software. Different letters indicate significant differences at *p* < 0.05 in the same group. *^c^* The commercial antifungal agent.

**Table 4:** Curative and protective activities of **W12** against *Phomopsis* sp. in vivo *^a b^*.

Compound	Concentration (μg/mL)	Curative Effect		Protective Effect	
		Lesion Length (mm ± SD)	Controlling Efficacy (%)	Lesion Length (mm ± SD)	Controlling Efficacy (%)
**W12**	200	11.4 ± 0.7 c	82.3	6.3 ± 0.9 d	96.2
	100	21.4 ± 0.6 b	54.6	9.8 ± 0.8 c	86.4
Azoxystrobin *^c^*	200	8.0 ± 0.5 d	91.7	6.9 ± 0.3 d	94.6
	100	12.3 ± 1.0 c	79.8	12.1 ± 1.1 b	80.0
Control	0	41.2 ± 1.5 a		40.5 ± 10.9 a	

*^a^* Values are mean ± SD of three replicates. *^b^* Statistical analysis was conducted by SPSS 25.0 software. Different letters indicate significant differences at *p* < 0.05 in the same group. *^c^* The commercial antifungal agent.

## Data Availability

All data generated in this study are presented in the current manuscript. No new datasets were generated. Data are available upon request from the corresponding author.

## Supplementary Materials

The following supporting information can be downloaded at: https://www.mdpi.com/article/10.3390/ijms24032599/s1.
