# Supplementary material for: Design, Synthesis and Antifungal Activity of Novel 1,4-Pentadiene-3-one Containing Quinazolinone
Source: Int J Mol Sci. 2023 Jan 30;24(3):2599. doi: 10.3390/ijms24032599 (PMC9916701; doi:10.3390/ijms24032599)
Supplement: Supplementary file 1 [file ijms-24-02599-s001.zip › ijms-2124541-supplementary.pdf]

Supplementary Materials

Design, synthesis and antifungal activity of novel 1, 4-pentadiene-3-one containing quinazolinone

Ran Zhou, Wenliang Zhan, Chunmei Yuan, Tao Zhang, Piao Mao, Zhiling Sun, Yousan An, Wei Xue*

National Key Laboratory of Green Pesticide, Key Laboratory of Green Pesticide and Agricultural Bioengineering, Ministry of Education, Center for R&D of Fine Chemicals of Guizhou University, Guiyang, 550025, China; rzhou12345@163.com (R.Z.); zwl1058542168@163.com (W.Z.); ycm13985689220@163.com (C.Y.); 15186949302@163.com (T.Z.); 15086014640@163.com (P.M.); 13312636928@163.com (Z.S.); anyoushan123456@163.com (Y.A.)
* Correspondence: wxue@gzu.edu.cn

The characterization data of the target compounds including ^{1}H NMR, ^{13}C NMR, ^{19}FNMRand HRMS were shown as below.

Data for 6-chloro-2-((3-(4-((1*E*,4*E*)-3-oxo-5-phenylpenta-1,4-dien-1-yl)phenoxy)propyl)thio)-3-phenylquinazolin-4(3*H*)-one **(W1).** Yellow solid; m.p. 198.2-199.6 °C; yield 58%; ^{1}HNMR (500 MHz, $CDCl_3$-*d6*) δ 8.18 (s, 1H, Ph-H), 7.70 (t, *J* = 12.9 Hz, 2H, 2-Ph-CH=CH-), 7.62 (d, *J* = 15.1 Hz, 3H, Ph-3H), 7.54 (s, 5H, -C-Ph-5H), 7.48 (d, *J* = 8.2 Hz, 1H, Ph-H), 7.40 (s, 3H, -N-Ph-3H), 7.29 (d, *J* = 2.6 Hz, 2H, -N-Ph-2H), 7.01 (dd, *J* = 58.1, 15.6 Hz, 2H, 2CO=CH), 6.88 (d, *J* = 6.0 Hz, 2H, -O-Ph-2H), 4.09 (d, *J* = 4.9 Hz, 2H, -S-$CH_2CH_2\underline{CH_2}$-O-), 3.32 (s, 2H, -S-$\underline{CH_2}CH_2CH_2$-O-), 2.22 (s, 2H, -S-$CH_2\underline{CH_2}CH_2$-O-); ^{13}C NMR (126 MHz, $CDCl_3$-*d6*) δ 188.98, 160.91, 160.85, 157.72, 146.36, 143.17, 143.02, 135.72, 135.08, 134.98, 131.51, 130.49, 130.24, 129.87, 129.12, 129.04, 128.44, 127.97, 127.72, 126.64 , 125.65, 123.50, 120.98, 115.00, 66.42, 29.30, 28.57; HRMS (ESI) calcd for $C_{34}H_{27}ClN_2O_3S$ $[M+H]^+$ 579.15037, found 579.15009.

Data for 6-chloro-3-(4-chlorophenyl)-2-((3-(4-((1*E*,4*E*)-3-oxo-5-phenylpenta-1,4-dien-1-yl)phenoxy)propyl)thio)quinazolin-4(3*H*)-one **(W2).** Yellow solid; m.p. 201.2-203.1 °C; yield 83%; ^{1}H NMR (500 MHz, $CDCl_3$-*d6*) δ 8.17 (d, *J* = 2.5 Hz, 1H, Cl-Ph-H), 7.69 (dd, *J* = 15.8, 6.8 Hz, 2H, 2-Ph-CH=CH-), 7.65 – 7.63 (m, 1H, Cl-Ph-H), 7.54 (d, *J* = 8.8 Hz, 2H, -O-Ph-2H), 7.53 – 7.49 (m, 6H, CH_3-Ph-4H, -Cl-Ph-2H), 7.48 (d, *J* = 5.6 Hz, 2H, -O-Ph-2H), 7.24 (s, 2H, -N-Ph-2H), 7.02 (d, *J* = 15.9 Hz, 1H, CO=CH), 6.95 (d, *J* = 15.9 Hz, 1H, CO=CH), 6.88 (d, *J* = 8.8 Hz, 2H, -O-Ph-2H), 4.09 (t, *J* = 5.9 Hz, 2H, -S-$CH_2CH_2\underline{CH_2}$-O-), 3.34 (t, *J* = 7.0 Hz, 2H, -S-$\underline{CH_2}CH_2CH_2$-O-), 2.22 (s, 2H, -S-$CH_2\underline{CH_2}CH_2$-O-); ^{13}C NMR (126 MHz, DMSO-d6) δ 189.03, 160.73, 159.37, 157.29, 146.26, 143.12, 142.86, 141.03, 136.42, 135.25, 134.09, 132.23, 131.70, 130.53, 130.21, 130.07, 129.79, 128.47, 128.02, 127.85, 126.64, 124.75, 123.59, 120.81, 114.97, 66.36, 29.79, 29.39; HRMS (ESI) calcd for $C_{34}H_{26}Cl_2N_2O_3S$ $[M+H]^+$ 613.11140, found 613.11035.

Data for 6-chloro-3-(2,6-dimethylphenyl)-2-((3-(4-((1*E*,4*E*)-3-oxo-5-phenylpenta-1,4-dien-1-yl)phenoxy)propyl)thio)quinazolin-4(3*H*)-one **(W3).** Yellow solid; m.p. 188. 6-189.9 °C; yield 53%; ^{1}HNMR (500 MHz, $CDCl_3$-*d6*) δ 8.25 (d, *J* = 2.5 Hz, 1H, Cl-Ph-1H), 7.72 (dd, *J* = 15.9, 9.3 Hz, 2H, 2-Ph-CH=CH-), 7.66 (dd, *J* = 8.7, 2.5 Hz, 1H, Cl-Ph-1H), 7.61 (dd, *J* = 6.5, 3.1 Hz, 2H, Ph-2H), 7.56 – 7.54 (m, 2H, Ph-2H), 7.52 (dd, *J* = 10.4, 5.5 Hz, 2H, O-Ph-2H), 7.40 (dd, *J* = 5.0, 1.9 Hz, 2H, -S-$CH_2\underline{CH_2}CH_2$-O-), 7.33 (dd, *J* = 13.2, 5.6 Hz, 2H, -O-Ph-2H), 7.21 (d, *J* = 7.7 Hz, 3H, -N-Ph-3H), 7.08 (dd, *J* = 12.1, 3.3 Hz, 1H, Cl-Ph-1H), 6.97 (dd, *J* = 15.9,

1.9 Hz, 1H, Ph-1H), 6.89 (dd, *J* = 8.8, 2.0 Hz, 2H, 2CO=CH), 4.09 (t, *J* = 6.0 Hz, 2H, -S-$CH_2CH_2\underline{CH_2}$-O-), 3.32 (t, *J* = 7.1 Hz, 2H, -S-$\underline{CH_2}CH_2CH_2$-O-), 2.10 (s, 6H, N-Ph-2CH_3). ^{13}C NMR (126 MHz, $CDCl_3$-*d_6*) δ 189.08, 160.86, 159.92, 157.64, 146.67, 143.27, 143.11, 136.55, 136.00, 135.17, 134.95, 133.94, 131.52, 130.54, 130.30, 129.81, 129.32, 129.08, 128.84, 128.47, 128.22, 127.97, 127.70, 126.84, 125.62, 123.47, 120.75, 116.82, 114.99, 66.38, 28.66, 28.63, 17.80; HRMS (ESI) calcd for $C_{36}H_{31}ClN_2O_3S$ $[M+H]^+$ 607.18167, found 607.18079.

Data for 3-(4-methoxyphenyl)-6-methyl-2-((3-(4-((1*E*,4*E*)-3-oxo-5-phenylpenta-1,4-dien-1-yl)phenoxy)propyl)thio)quinazolin-4(3*H*)-one **(W4).** Yellow solid; m.p. 178. 3-179.9 °C; yield 71%; 1H NMR (500 MHz, $CDCl_3$-*d_6*) δ 8.01 (s, 1H, CH_3-Ph-H), 7.71 (s, 2H, 2-Ph-$\underline{CH}$=CH-), 7.61 – 7.50 (m, 4H, 2-CH=CH-Ph-2H), 7.42 (d, *J* = 24.8 Hz, 4H, -N-Ph-2H, CH3-Ph-2H), 7.19 (s, 2H, CH_3-Ph-2H), 7.05 (d, *J* = 29.8 Hz, 3H, -O-Ph-3H), 6.95 (d, *J* = 16.1 Hz, 1H, CH_3-O-Ph-H), 6.89 (s, 2H, 2CO=CH), 4.09 (s, 2H, -S-$CH_2CH_2\underline{CH_2}$-O-), 3.85 (s, 3H, -O-CH_3), 3.31 (s, 2H, -S-$\underline{CH_2}CH_2CH_2$-O-), 2.44 (s, 3H, Ph-CH_3), 2.22 (s, 2H, -S-$CH_2\underline{CH_2}CH_2$-O-). ^{13}C NMR (126 MHz, $CDCl_3$-*d_6*) δ 189.01, 162.29, 160.94, 160.58, 156.54, 145.94, 143.27, 143.00, 136.10, 136.01, 134.97, 130.51, 130.34, 130.27, 129.05, 128.53, 128.46, 127.61, 126.76, 126.11, 125.64, 123.40, 119.64, 115.02, 114.97, 66.53, 55.61, 29.16, 28.61, 21.36; HRMS (ESI) calcd for $C_{36}H_{32}N_2O_4S$ $[M+H]^+$ 589.21555, found 589.21509.

Data for 6-methyl-2-((3-(4-((1*E*,4*E*)-3-oxo-5-phenylpenta-1,4-dien-1-yl)phenoxy)propyl)thio)-3-(p-tolyl)quinazolin-4(3*H*)-one **(W5).** Yellow solid; m.p. 166.5-167.8 °C; yield 52%; ^{1}HNMR (500 MHz, $CDCl_3$-*d_6*) δ 8.01 (s, 1H, CH_3-Ph-1H), 7.73 (d, *J* = 10.5 Hz, 1H, -Ph-$\underline{CH}$=CH-), 7.69 (d, *J* = 10.4 Hz, 1H, -Ph-$\underline{CH}$=CH-), 7.63 – 7.58 (m, 2H, -O-Ph-2H), 7.53 (dd, *J* = 11.0, 8.5 Hz, 3H, -Ph-3H), 7.45 (s, 1H, CH_3-Ph-1H), 7.40 (dd, *J* = 5.1, 2.0 Hz, 2H, -O-Ph-2H), 7.33 (d, *J* = 8.1 Hz, 3H,-N-Ph-3H), 7.18 (d, *J* = 8.2 Hz, 2H, -Ph-2H), 7.07 (d, *J* = 15.9 Hz, 1H, CH_3-Ph-1H), 6.95 (d, *J* = 15.9 Hz, 1H, -N-Ph-1H), 6.89 (d, *J* = 8.8 Hz, 2H, 2CO=CH), 4.09 (t, *J* = 6.0 Hz, 2H, -S-$CH_2CH_2\underline{CH_2}$-O-), 3.32 (t, *J* = 7.0 Hz, 2H, -S-$\underline{CH_2}CH_2CH_2$-O-), 2.44 (d, *J* = 6.0 Hz, 5H, Ph-CH_3, -S-$CH_2\underline{CH_2}CH_2$-O-), 2.35 – 2.13 (m, 3H, -N-Ph-CH_3); ^{13}C NMR (126 MHz, $CDCl_3$-*d_6*) δ 188.98, 162.11, 160.95, 156.16, 145.95, 143.24, 142.97, 140.20, 136.07, 136.00, 135.00, 133.46, 130.45, 130.25, 130.21, 129.04, 128.93, 128.44, 128.17, 127.64, 126.75, 126.11, 125.67, 123.43, 119.68, 115.03, 66.53, 29.13, 28.65, 21.52, 21.33; HRMS (ESI) calcd for $C_{36}H_{32}N_2O_3S$ $[M+H]^+$ 573.22064, found 573,21960.

Data for 6-chloro-2-((3-(4-((1*E*,4*E*)-3-oxo-5-(p-tolyl)penta-1,4-dien-1-yl)phenoxy)pro pyl)thio)-3-phenylquinazolin-4(3*H*)-one **(W6).** Yellow solid; m.p. 215.3-216.7 °C; yield 58%; ^{1}HNMR (500 MHz, $CDCl_3$-*d_6*) δ 8.18 (s, 1H, Ph-H), 7.69 (dd, *J* = 15.5, 5.6 Hz, 2H, 2-Ph-$\underline{CH}$=CH-), 7.65 – 7.62 (m, 1H, Ph-H), 7.54 (s, 4H, 2-Ph-2H), 7.49 (s, 3H, -N-Ph-3H), 7.47 (s, 1H, Ph-H), 7.29 (s, 2H, -N-Ph-2H), 7.20 (d, *J* = 6.3 Hz, 2H, Ph-H), 6.98 (dd, *J* = 37.5, 15.8 Hz, 2H, 2CO=CH), 6.88 (d, *J* = 7.0 Hz, 2H, -O-Ph-H), 4.08 (s, 2H, -S-$CH_2CH_2\underline{CH_2}$-O-), 3.32 (s, 2H, -S-$\underline{CH_2}CH_2CH_2$-O-), 2.38 (s, 3H, Ph-CH_3), 2.22 (d, *J* = 4.8 Hz, 2H, -S-$CH_2\underline{CH_2}CH_2$-O-); ^{13}CNMR (126 MHz, DMSO-*d_6*) δ 189.05, 160.92, 160.78, 157.72, 146.35, 143.11, 142.91, 141.02, 135.71, 135.09, 132.23, 131.52, 130.20, 129.88, 129.79, 129.12, 128.47, 127.97, 127.79, 126.64, 124.75, 123.55, 120.98, 114.98, 66.41, 29.31, 28.57, 21.62; HRMS (ESI) calcd for $C_{35}H_{29}ClN_2O_3S$ $[M+H]^+$ 593.16602, found 593.16516.

Data for 6-chloro-3-(4-chlorophenyl)-2-((3-(4-((1*E*,4*E*)-3-oxo-5-(p-tolyl)penta-1,4-dien-1-yl)phenoxy)propyl)thio)quinazolin-4(3*H*)-one **(W7).** Yellow solid; m.p. 189.5-190.7 °C; yield 66%; ^{1}H NMR (500 MHz, $CDCl_3$-*d_6*) δ 8.16 (s, 1H, Cl-Ph-H), 7.69 (d, *J* = 15.9 Hz, 2H, 2-Ph-CH=CH-), 7.63 (s, 1H, Cl-Ph-H), 7.60 – 7.48 (m, 6H, CH_3-Ph-4H, -Cl-Ph-2H), 7.35 (d, *J* = 45.7 Hz, 2H, -O-Ph-2H), 7.24 (s, 3H, -N-Ph-3H), 6.98 (dd, *J* = 35.9, 15.8 Hz, 2H, 2CO=CH), 6.89 (s, 2H, -O-Ph-2H), 4.08 (s, 2H, -S-$CH_2CH_2\underline{CH_2}$-O-O-), 3.33 (s, 2H, -S-$\underline{CH_2}CH_2CH_2$-O-), 2.38 (s, 3H, Ph-CH_3), 2.22 (s, 2H, -S-$CH_2\underline{CH_2}CH_2$-O-); ^{13}CNMR (126 MHz, $CDCl_3$-*d_6*) δ

189.05, 160.92, 160.78, 157.27, 146.35, 143.11, 142.91, 141.02, 135.71, 135.09, 132.23, 131.52, 130.20, 129.88, 129.79, 129.12, 128,47, 127.97, 127,79, 126.64, 124.75, 123.55, 120.98, 114.98, 66.41, 29.79, 29.31, 28.57; HRMS (ESI) calcd for $C_{35}H_{28}Cl_2N_2O_3S$ [M+H]$^+$ 627.12705, found 627.12671.

Data for 6-chloro-3-(2,6-dimethylphenyl)-2-((3-(4-((1*E*,4*E*)-3-oxo-5-(p-tolyl)penta-1,4 -dien-1-yl)phenoxy)propyl)thio)quinazolin-4(3*H*)-one **(W8).** Yellow solid; m.p. 210.3-211.8 ˚C; yield 55%; 1HNMR (500 MHz, $CDCl_3$-*d6*) δ 8.21 (d, *J* = 2.5 Hz, 1H, Cl-Ph-1H), 7.71 (d, *J* = 6.1 Hz, 1H, -Ph-CH=CH-), 7.68 (d, *J* = 6.1 Hz, 1H, -Ph-CH=CH-), 7.66 – 7.64 (m, 1H, Cl-Ph-1H), 7.54 (d, *J* = 8.7 Hz, 2H, -O-Ph-2H), 7.50 (d, *J* = 8.5 Hz, 3H, -N-Ph-3H), 7.32 (d, *J* = 7.6 Hz, 1H, Cl-Ph-1H), 7.21 (d, *J* = 7.8 Hz, 4H, CH_3-Ph-4H), 7.02 (d, *J* = 15.9 Hz, 1H, -O-Ph-1H)), 6.95 (d, *J* = 15.9 Hz, 1H, -O-Ph-1H), 6.90 – 6.87 (m, 2H, 2CO=CH), 4.09 (t, *J* = 6.0 Hz, 2H, -S-$CH_2CH_2\underline{CH_2}$-O-), 3.31 (t, *J* = 7.1 Hz, 2H, --S-$\underline{CH_2}CH_2CH_2$-O-), 2.38 (s, 3H, Ph-CH_3), 2.23 (s, 2H, -S-$CH_2\underline{CH_2}CH_2$-O-), 2.10 (s, 6H, N-Ph-2CH_3); ^{13}C NMR (126 MHz, $CDCl_3$-*d6*) δ 189.02, 160.78, 159.80, 157.68, 142.66, 143.09, 142.88, 141.01, 136.53, 135.09, 133.95, 132.24, 131.47, 130.27, 130.21 129.79, 129.05, 128.46, 127.96, 127.81, 126.77, 124.76, 123.57, 120.80, 114.98, 66.40, 29.79, 28.69, 28.63, 21.62, 17.75; HRMS (ESI) calcd for $C_{37}H_{33}ClN_2O_3S$ [M+H]$^+$ 621.19732, found 621.19635.

Data for 3-(4-methoxyphenyl)-6-methyl-2-((3-(4-((1*E*,4*E*)-3-oxo-5-(p-tolyl)penta-1,4-dien-1-yl)phenoxy)propyl)thio)quinazolin-4(3*H*)-one **(W9).** Yellow solid; m.p. 199.6-200.8 ˚C; yield 62%; ^{1}HNMR (500 MHz, DMSO-*d6*) δ 8.01 (s, 1H, CH_3-Ph-H), 7.69 (d, *J* = 15.7 Hz, 2H, 2-Ph-$\underline{CH}$=CH-), 7.51 (d, *J* = 9.7 Hz, 5H, CH_3-Ph-H, 2-CH=CH-Ph-2H), 7.44 (s, 1H, CH_3-Ph-H), 7.20 (d, *J* = 2.7 Hz, 4H, -N-Ph-2H, CH3-Ph-2H), 7.02 (d, *J* = 10.4 Hz, 3H, -O-Ph-3H), 6.97(s, 1H,CH_3-O-Ph-H), 6.87 (d, 2H, 2CO=CH), 4.09 (s, 2H, -S-$CH_2CH_2\underline{CH_2}$-O-), 3.85 (s, 3H, -O-CH_3), 3.31 (s, 2H, -S-$\underline{CH_2}CH_2CH_2$-O-), 2.44 (s, 3H, Ph-CH_3), 2.37 (s, 3H, -CH-Ph-CH_3), 2.23 (s, 2H, -S-$CH_2\underline{CH_2}CH_2$-O-); ^{13}C NMR (126 MHz, $CDCl_3$-*d6*) δ 189.08, 162.29, 160.87, 160.58, 156.54, 145.94, 143.08, 143.00, 141.03, 136.10, 136.00, 132.22, 130.33, 130.22, 129.80, 129.45, 128.53, 128.48, 127.68, 126.76, 126.11, 124.75, 123.46, 119.64, 114.99, 114.97, 114.90, 66.52, 55.61, 29.16, 28.61, 21.65, 21.35; HRMS (ESI) calcd for $C_{37}H_{34}N_2O_4S$ [M+H]$^+$ 603.23120, found 603.23016

Data for 6-methyl-2-((3-(4-((1*E*,4*E*)-3-oxo-5-(p-tolyl)penta-1,4-dien-1-yl)phenoxy)pro pyl)thio)-3-(p-tolyl)quinazolin-4(3*H*)-one **(W10).** Yellow solid; m.p. 187.6-188.1 ˚C; yield 53%; ^{1}HNMR (500 MHz, $CDCl_3$-*d6*) δ 8.01 (s, 1H, CH_3-Ph-1H), 7.69 (dd, *J* = 15.9, 6.3 Hz, 2H, 2-Ph-$\underline{CH}$=CH-), 7.55 – 7.50 (m, 5H, -N-Ph-4H, CH_3-Ph-1H), 7.45 (s, 1H, CH_3-Ph-1H), 7.33 (d, *J* = 8.2 Hz, 2H, -O-Ph-2H), 7.21 (d, *J* = 7.9 Hz, 2H, CH3-Ph-2H), 7.17 (d, *J* = 8.1 Hz, 2H, CH_3-Ph-2H), 7.02 (d, *J* = 15.9 Hz, 1H, -O-Ph-1H), 6.95 (d, *J* = 15.9 Hz, 1H, -O-Ph-1H), 6.89 (d, *J* = 8.6 Hz, 2H, 2CO=CH), 4.09 (t, *J* = 6.0 Hz, 2H, -S-$CH_2CH_2\underline{CH_2}$-O-), 3.31 (t, *J* = 7.0 Hz, 2H, -S-$\underline{CH_2}CH_2CH_2$-O-), 2.44 (d, *J* = 6.0 Hz, 8H, Ph-CH_3, -S-$CH_2\underline{CH_2}CH_2$-O-, -O-CH_3), 2.38 (s, 3H, -N-Ph-CH_3); ^{13}C NMR (126 MHz, $CDCl_3$-*d6*) δ 189.09, 162.14, 160.88, 156.15, 145.94, 143.09, 143.02, 141.03, 140.23, 136.10, 136.02, 133.43, 132.22, 130.48, 130.23, 129.80, 128.92, 128.48, 127.68, 126.75, 126.11, 124.75, 123.46, 119.66, 114.99, 66.49, 29.13, 28.63, 21.65, 21.55, 21.35; HRMS (ESI) calcd for $C_{37}H_{34}N_2O_3S$ [M+H]$^+$ 587.23629, found 587.23541.

Data for 6-chloro-2-((3-(4-((1*E*,4*E*)-5-(4-fluorophenyl)-3-oxopenta-1,4-dien-1-yl)phenox y)propyl)thio)-3-phenylquinazolin-4(3*H*)-one **(W11).** Yellow solid; m.p. 184.5-186.2 ˚C; yield 62%; ^{1}H NMR (500 MHz, $CDCl_3$-*d6*) δ 8.17 (s, 1H, Cl-Ph-H), 7.71 – 7.57 (m, 5H, 2-Ph-2H, Ph-H), 7.53 (s, 5H, -N-Ph-3H, 2-Ph-$\underline{CH}$=CH-), 7.48 (s, 1H, Ph-H), 7.29 (s, 2H, -N-Ph-2H), 7.09 (s, 2H, Ph-H), 6.95 (dd, *J* = 37.8, 22.5 Hz, 4H, 2CO=CH, -O-Ph-2H), 4.09 (s, 2H, -S-$CH_2CH_2\underline{CH_2}$-O-), 3.32 (s, 2H, -S-$\underline{CH_2}CH_2CH_2$-O-), 2.22 (s, 2H, -S-$CH_2\underline{CH_2}CH_2$-O-).

^{13}C NMR (126 MHz, $CDCl_3$-*d_6*) δ 188.74, 165.06, 163.06, 160.93, 160.89, 157.71, 146.35, 143.27, 141.71, 135.70, 135.10, 131.51, 131.22, 130.36, 130.28, 129.90, 129.12, 127.98, 127.63, 126.64, 125.29, 123.46, 120.97, 116.30, 116.13, 114.99, 66.40, 29.29, 28.54; ^{19}F NMR (471 MHz, $CDCl_3$-*d_6*) δ -109.16; HRMS (ESI) calcd for $C_{34}H_{26}ClFN_2O_3S$ $[M+H]^+$ 597.14095, found 597.14044.

Data for 6-chloro-3-(4-chlorophenyl)-2-((3-(4-((1*E*,4*E*)-5-(4-fluorophenyl)-3-oxopenta-1,4-dien-1-yl)phenoxy)propyl)thio)quinazolin-4(3*H*)-one **(W12).** Yellow solid; m.p. 200.5-202.1 °C; yield 42%; ^{1}HNMR (500 MHz, $CDCl_3$-*d_6*) δ 8.16 (d, *J* = 2.5 Hz, 1H, Cl-Ph-H), 7.69 (dd, *J* = 15.9, 6.0 Hz, 2H, 2-Ph-<u>CH</u>=CH-), 7.65 – 7.63 (m, 1H, Cl-Ph-H), 7.59 (dd, *J* = 8.7, 5.4 Hz, 2H, F-Ph-2H), 7.54 (d, *J* = 8.8 Hz, 2H, F-Ph-2H), 7.52 – 7.50 (m, 2H, -O-Ph-2H), 7.48 (d, *J* = 8.6 Hz, 2H, -O-Ph-2H), 7.24 – 7.22 (m, 2H, -N-Ph-2H), 7.09 (s, 1H, Cl-Ph-H), 6.99 (d, *J* = 15.9 Hz, 1H, -N-Ph-1H), 6.93 (d, *J* = 15.8 Hz, 1H, -N-Ph-1H), 6.89 (d, *J* = 8.8 Hz, 2H, 2CO=CH), 4.09 (t, *J* = 5.9 Hz, 2H, -S-CH_2CH_2<u>CH_2</u>-O-), 3.34 (t, *J* = 7.0 Hz, 2H, -S-<u>CH_2</u>CH_2CH_2-O-), 2.23 (s, 2H, -S-CH_2<u>CH_2</u>CH_2-O-).^{13}C NMR (126 MHz,) δ 188.74, 160.84, 157.27, 146.26, 143.25, 141.73, 136.42, 135.27, 134.07, 131.69, 130.53, 130.36, 130.29, 130.23, 128.03, 127.68, 126.64, 125.28, 123.50, 120.79, 116.31, 116.13, 114.99, 66.35, 29.80, 29.38, 28.50; ^{19}F NMR (471 MHz, $CDCl_3$-*d_6*) δ -109.16 (s); HRMS (ESI) calcd for $C_{34}H_{25}Cl_2$ FN_2O_3S $[M+H]^+$ 631.10197, found 631.09991.

Data for 6-chloro-3-(2,6-dimethylphenyl)-2-((3-(4-((1*E*,4*E*)-5-(4-fluorophenyl)-3-oxopen ta-1,4-dien-1-yl) phenoxy)propyl)thio)quinazolin-4(3*H*)-one **(W13).** Yellow solid; m.p. 197.3-198.9 °C; yield 53%; ^{1}H NMR (500 MHz, $CDCl_3$-*d_6*) δ 8.22 (d, *J* = 2.5 Hz, 1H, Cl-Ph-1H), 7.69 (dd, *J* = 15.9, 7.1 Hz, 2H, 2-Ph-<u>CH</u>=CH-), 7.65 (dd, *J* = 8.7, 2.5 Hz, 1H, -O-Ph-2H), 7.61 – 7.57 (m, 2H, -N-Ph-2H), 7.54 (d, *J* = 8.8 Hz, 2H, F-Ph-2H), 7.50 (d, *J* = 8.7 Hz, 1H, -N-Ph-H), 7.34 (d, *J* = 7.6 Hz, 1H, Cl-Ph-1H), 7.21 (d, *J* = 7.7 Hz, 2H, F-Ph-2H), 7.09 (t, *J* = 8.6 Hz, 2H, -O-Ph-2H), 6.99 (d, *J* = 15.9 Hz, 1H, -O-Ph-1H), 6.93 (d, *J* = 15.9 Hz, 1H, -O-Ph-1H), 6.90 – 6.87 (m, 2H, 2CO=CH), 4.09 (t, *J* = 6.0 Hz, 2H, -S-CH_2CH_2<u>CH_2</u>-O-), 3.32 (t, *J* = 7.1 Hz, 2H, -S-<u>CH_2</u>CH_2CH_2-O-), 2.23 (t, *J* = 6.8 Hz, 2H, -S-CH_2<u>CH_2</u>CH_2-O-), 2.10 (s, 6H, N-Ph-2CH_3); ^{13}C NMR (126 MHz, $CDCl_3$-*d_6*) δ 188.74, 165.06, 163.06, 160.89, 159.83, 157.67, 146.66, 143.26, 141.70, 136.54, 135.11, 133.95, 131.49, 131.25, 131.22, 130.34, 130.27, 129.06, 128.79, 127.96, 127.68, 126.79, 125.33, 123.50, 120.79, 116.29, 116.12, 115.01, 66.41, 28.68, 28.62, 17.76; ^{19}F NMR (471 MHz, $CDCl_3$-*d_6*) δ -109.15 (s); HRMS (ESI) calcd for $C_{36}H_{30}ClFN_2O_3S$ $[M+H]^+$ 625.17225, found 625.17133.

Data for 2-((3-(4-((1*E*,4*E*)-5-(4-fluorophenyl)-3-oxopenta-1,4-dien-1-yl)phenoxy)propyl) thio)-3-(4-methoxyphenyl)-6-methylquinazolin-4(3*H*)-one **(W14).** Yellow sol id; m.p. 185.5-186.2 °C; yield 63%; ^{1}HNMR (500 MHz, DMSO-*d_6*) δ 8.01 (s, 1H, CH_3-Ph-H), 7.70 (d, *J* = 15.7 Hz, 2H, 2-Ph-<u>CH</u>=CH-), 7.58 – 7.43 (m, 5H, 5H, CH_3-Ph-H, 2-CH=CH-Ph-2H), 7.21 – 7.18 (m, 2H, -N-Ph-2H), 7.08 (s, 2H, CH_3-Ph-2H), 7.01 (d, *J* = 7.4 Hz, 3H, -O-Ph-3H), 6.98 – 6.83 (m, 4H, 2CO=CH, CH_3-Ph-2H), 4.09 (s, 2H, -S-CH_2CH_2<u>CH_2</u>-O-), 3.85 (s, 3H, -O-CH_3), 3.30 (s, 2H, -S-<u>CH_2</u>CH_2CH_2-O-), 2.44 (s, 3H, Ph-CH_3), 2.22 (s, 2H, -S-CH_2<u>CH_2</u>CH_2-O-); ^{13}C NMR (126 MHz, $CDCl_3$-*d_6*) δ 188.76, 162.28, 160.98, 160.58, 156.54, 145.94, 143.35, 141.67, 136.10, 136.01, 130.34, 130.29, 130.05, 128.53, 127.56, 126.76, 126.11, 125.32, 123.40, 119.64, 116.30, 116.12, 115. 03, 114.97, 66.53, 55.61, 29.16, 28.61, 21.35; ^{19}F NMR (471 MHz, $CDCl_3$-*d_6*) δ -109.15 (s); HRMS (ESI) calcd for $C_{36}H_{31}FN_2O_4S$ $[M+H]^+$ 607.20613, found 607.20544.

Data for 2-((3-(4-((1*E*,4*E*)-5-(4-fluorophenyl)-3-oxopenta-1,4-dien-1-yl)phenoxy)propyl) thio)-6-methyl-3-(p-tolyl)quinazolin-4(3*H*)-one **(W15).** Yellow solid; m.p. 164. 0-166.2 °C; yield 42%; ^{1}HNMR (500 MHz, $CDCl_3$-*d_6*) δ 8.02 (dd, *J* = 1.3, 0.6 Hz, 1H, CH_3-Ph-1H), 7.71 (dd, *J* = 15.9, 10.2 Hz, 2H, 2-Ph-<u>CH</u>=CH-), 7.61 –

7.60 (m, 1H, CH_3-Ph-1H), 7.55 – 7.49 (m, 4H, F-Ph-4H), 7.44 (d, *J* = 8.3 Hz, 1H, CH_3-Ph-1H), 7.40 (dd, *J* = 5.1, 1.9 Hz, 2H, -N-Ph-2H), 7.21 – 7.19 (m, 2H, -N-Ph-2H), 7.07 (d, *J* = 15.9 Hz, 1H, -O-Ph-H), 7.03 – 7.01 (m, 2H, -O-Ph-2H), 6.95 (dd, *J* = 15.8, 2.2 Hz, 1H, -O-Ph-H), 6.89 (dd, *J* = 8.5, 1.5 Hz, 2H, 2CO=CH), 4.10 (t, *J* = 6.0 Hz, 2H, -S-$CH_2CH_2\underline{CH_2}$-O-), 3.85 (s, 3H, -O-CH_3), 3.31 (t, *J* = 7.0 Hz, 2H, -S-$\underline{CH_2}CH_2CH_2$-O-), 2.45 (s, 3H, Ph-CH_3), 2.23 (s, 2H, -S-$CH_2\underline{CH_2}CH_2$-O-); ^{13}C NMR (126 MHz, $CDCl_3$-*d*$_6$) δ 188.98, 162.26, 160.95, 160.60, 156.56, 145.95, 143.24, 142.97, 136.08, 135.99, 135.00, 130.48 130.35, 130.25, 130.21, 129.79, 129.04, 128.56, 128.44, 127.65, 126.76, 126.11, 125.67, 123.43, 119.66, 115.03, 114.97, 114.87, 66.56, 55.60, 29.17, 28.64, 21.33; ^{19}F NMR (471 MHz, $CDCl_3$-*d*$_6$) δ -109.13 (s); HRMS (ESI) calcd for $C_{36}H_{31}FN_2O_3S$ $[M+H]^+$ 591.21122, found 591.20880.

Data for 2-((3-(4-((1*E*,4*E*)-5-(4-bromophenyl)-3-oxopenta-1,4-dien-1-yl)phenoxy)propyl) thio)-6-chloro-3-phenylquinazolin-4(3*H*)-one **(W16).** Yellow solid; m.p. 192.6-194.1 °C; yield 53%; ^1H NMR (500 MHz, $CDCl_3$-*d*$_6$) δ 8.18 (d, *J* = 2.4 Hz, 1H, Cl-Ph-H), 7.69 (d, *J* = 15.8 Hz, 1H, Ph-H), 7.64 (dd, *J* = 9.1, 6.5 Hz, 2H, 2-Ph-$\underline{CH}$=CH-), 7.53 (dd, *J* = 6.4, 2.3 Hz, 6H, 2-Ph-2H, -N-Ph-2H), 7.49 (s, 1H, Ph-H), 7.47 – 7.45 (m, 2H, Ph-H), 7.31 – 7.28 (m, 3H, -N-Ph-3H), 7.05 (d, *J* = 15.9 Hz, 1H, CO=CH), 6.92 (d, *J* = 15.9 Hz, 1H, CO=CH), 6.88 (d, *J* = 8.8 Hz, 2H, -O-Ph-2H), 4.09 (t, *J* = 6.0 Hz, 2H, -S-$CH_2CH_2\underline{CH_2}$-O-), 3.32 (t, *J* = 7.0 Hz, 2H, -S-$\underline{CH_2}CH_2CH_2$-O-), 2.22 (s, 2H, -S-$CH_2\underline{CH_2}CH_2$-O-); ^{13}C NMR (126 MHz, $CDCl_3$-*d*$_6$) δ 188.65, 160.95, 157.71, 146.35, 143.45, 141.55, 135.71, 135.08, 133.92, 132.28, 131.52, 130.29, 130.23, 129.87, 128.78, 129.12, 128.41, 127.96, 127.61, 126.64, 126.06, 124.71, 123.45, 120.98, 115.02, 66.43, 29.79, 29.29. HRMS (ESI) calcd for $C_{34}H_{26}BrClN_2O_3S$ $[M+H]^+$ 657.06088, found 657,06061.

Data for 2-((3-(4-((1*E*,4*E*)-5-(4-bromophenyl)-3-oxopenta-1,4-dien-1-yl)phenoxy)prop yl)thio)-6-chloro-3-(4-chlorophenyl)quinazolin-4(3*H*)-one **(W17).** Yellow solid; m.p. 173.5-174.0 °C; yield 33%; ^1HNMR (500 MHz, $CDCl_3$-*d*$_6$) δ 8.16 (s, 1H, Cl-Ph-H), 7.66 (dd, *J* = 25.8, 14.1 Hz, 3H, 2-Ph-$\underline{CH}$=CH-, Cl-Ph-H), 7.51 (d, *J* = 15.3 Hz, 6H, Br-Ph-4H, -Cl-Ph-2H), 7.47 (s, 3H, -O-Ph-3H), 7.23 (s, 2H, -N-Ph-2H), 7.13 – 6.92 (m, 2H, 2CO=CH), 6.89 (s, 2H, -O-Ph-2H), 4.09 (d, *J* = 4.5 Hz, 2H, -S-$CH_2CH_2\underline{CH_2}$-O-), 3.33 (s, 2H, -S-$\underline{CH_2}CH_2CH_2$-O-), 2.22 (s, 2H, -S-$CH_2\underline{CH_2}CH_2$-O-); ^{13}C NMR (126 MHz, $CDCl_3$-*d*$_6$) δ 188.66, 160.81, 157.27, 146.26, 143.44, 141.59, 136.42, 135.26, 134.07, 133.89, 132.29, 131.69, 130.53, 130.32, 130.23, 129.80, 128.02, 127.63, 126.64, 126.03, 124.74, 123.46, 120.80, 115.00, 66.35, 29.81, 29.37; HRMS (ESI) calcd for $C_{34}H_{25}BrCl_2N_2O_3S$ $[M+Na]^+$ 713.00385, found 713.00183.

Data for 2-((3-(4-((1*E*,4*E*)-5-(4-bromophenyl)-3-oxopenta-1,4-dien-1-yl)phenoxy)prop yl)thio)-6-chloro-3-(2,6-dimethylphenyl)quinazolin-4(3*H*)-one **(W18).** Yellow solid; m.p. 189.5-191.1 °C; yield 41%; ^1HNMR (500 MHz, $CDCl_3$-*d*$_6$) δ 8.22 (d, *J* = 2.5 Hz, 1H, Cl-Ph-1H), 7.69 (dd, *J* = 15.9, 7.1 Hz, 2H, 2-Ph-$\underline{CH}$=CH-), 7.65 (dd, *J* = 8.7, 2.5 Hz, 1H, -O-Ph-H), 7.59 (dd, *J* = 8.7, 5.4 Hz, 2H, -N-Ph-2H), 7.55 – 7.53 (m, 2H, Br-Ph-2H), 7.50 (d, *J* = 8.7 Hz, 1H, -N-Ph-H), 7.34 (d, *J* = 7.6 Hz, 1H, Cl-Ph-1H), 7.21 (d, *J* = 7.7 Hz, 2H, Br-Ph-2H), 7.11 – 7.07 (m, 2H, -O-Ph-2H), 6.99 (d, *J* = 15.9 Hz, 1H, -O-Ph-1H), 6.93 (d, *J* = 15.9 Hz, 1H, -O-Ph-1H), 6.89 (d, *J* = 8.8 Hz, 2H, 2CO=CH), 4.09 (t, *J* = 6.0 Hz, 2H, -S-$CH_2CH_2\underline{CH_2}$-O-), 3.32 (t, *J* = 7.1 Hz, 2H, -S-$\underline{CH_2}CH_2CH_2$-O-), 2.23 (t, *J* = 6.8 Hz, 2H, -S-$CH_2\underline{CH_2}CH_2$-O-), 2.10 (s, 6H, N-Ph-2CH_3). ^{13}C NMR (126 MHz, $CDCl_3$-*d*$_6$) δ 188.73, 160.96, 159.90, 157.64, 146.67, 143.52, 141.62, 136.55, 135.93, 135.14, 133.96, 133.92, 132.28, 131.52, 130.33, 130.28, 129.80, 129.27, 129.07, 128.82, 128.18, 127.96, 127.62, 126.83, 126.06, 124.74, 123.45, 120.77, 115.03, 66.42, 29.79, 28.68, 28.63, 17.78; HRMS (ESI) calcd for $C_{36}H_{30}BrClN_2O_3S$ $[M+H]^+$ 685.09218, found 685.09186.

Data for 2-((3-(4-((1E,4E)-5-(4-bromophenyl)-3-oxopenta-1,4-dien-1-yl)phenoxy)prop yl)thio)-3-(4-methoxyphenyl)-6-methylquinazolin-4(3H)-one **(W19).** Yellow solid; m.p. 191.8-192.7 °C; yield 67%; ^{1}HNMR (500 MHz, $CDCl_3$-d_6) δ 8.01 (s, 1H, CH_3-Ph-H), 7.71 – 7.67 (m, 1H, -Ph-CH=CH-), 7.66 – 7.62 (m, 1H, -Ph-CH=CH-), 7.54 (t, J = 2.1 Hz, 2H, CH_3-Ph-2H,), 7.52 (d, J = 2.0 Hz, 3H, -CH=CH-Ph-2H, Ph-H), 7.51 (t, J = 2.3 Hz, 1H, CH_3-Ph-H), 7.47 – 7.44 (m, 3H, -O-Ph-3H), 7.21 – 7.19 (m, 2H, CH_3-Ph-2H), 7.03 – 7.01 (m, 3H, -N-Ph-3H), 6.89 (dd, J = 8.5, 1.5 Hz, 2H, 2CO=CH), 4.10 (t, J = 6.0 Hz, 2H, -S-$CH_2CH_2$$CH_2$-O-), 3.86 (s, 3H, -O-$CH_3$), 3.31 (t, J = 7.0 Hz, 2H, -S-$CH_2$$CH_2CH_2$-O-), 2.45 (s, 3H, Ph-$CH_3$), 2.23 (t, J = 6.6 Hz, 2H, -S-$CH_2$$CH_2$$CH_2$-O-); ^{13}C NMR (126 MHz, $CDCl_3$-d_6) δ 188.68, 162.28, 161.03, 160.58, 156.53, 145.94, 143.55, 141.53, 136.09, 136.01, 133.91, 132.28, 130.33, 129.80, 128.53, 127.50, 126.77, 126.10, 126.05, 124.71, 123.35, 119.64, 115.04, 1114.97, 66.54, 55.61, 29.81, 29.16, 28.61, 21.35. HRMS (ESI) calcd for $C_{36}H_{31}BrN_2O_4S$ $[M+H]^+$ 667.12607, found 667.12518.

Data for 2-((3-(4-((1E,4E)-5-(4-bromophenyl)-3-oxopenta-1,4-dien-1-yl)phenoxy)prop yl)thio)-6-methyl-3-(p-tolyl)quinazolin-4(3H)-one **(W20).** Yellow solid; m.p. 178.1-180.5 °C; yield 29%; ^{1}H NMR (500 MHz, $CDCl_3$-d_6) δ 8.01 (d, J = 0.5 Hz, 1H, CH_3-Ph-1H), 7.69 (d, J = 15.8 Hz, 1H, -Ph-CH=CH-), 7.64 (d, J = 15.9 Hz, 1H, -Ph-CH=CH-), 7.53 (dd, J = 8.6, 2.0 Hz, 5H, CH_3-Ph-1H, Br-Ph-4H), 7.46 (d, J = 8.2 Hz, 3H, -N-Ph-3H), 7.33 (d, J = 8.1 Hz, 2H, -O-Ph-2H), 7.17 (d, J = 8.2 Hz, 2H, -O-Ph-2H), 7.05 (d, J = 15.9 Hz, 1H, -N-Ph-H), 6.90 (dd, J = 15.1, 12.3 Hz, 3H, 2CO=CH, -O-Ph-H), 4.09 (t, J = 6.0 Hz, 2H, -S-$CH_2CH_2$$CH_2$-O-), 3.31 (t, J = 7.0 Hz, 2H, -S-$CH_2$$CH_2CH_2$-O-), 2.44 (d, J = 6.0 Hz, 6H, Ph-CH_3, -O-CH_3), 2.23 (s, 2H, -S-$CH_2$$CH_2$$CH_2$-O-); ^{13}C NMR (126 MHz, $CDCl_3$-d_6) δ 188.67, 162.12, 161.03, 156.13, 145.93, 143.54, 141.52, 140.23, 136.09, 136.01, 133.91, 133.43, 132.28, 130.47, 130.31, 129.79, 128.92, 127.49, 126.75, 126.10, 126.05, 124.71, 123.35, 119.66, 115.03, 66.51, 29.11, 28.62, 21.55, 21.35; HRMS (ESI) calcd for $C_{36}H_{31}BrN_2O_3S$ $[M+H]^+$ 651.13115, found 651.12970.

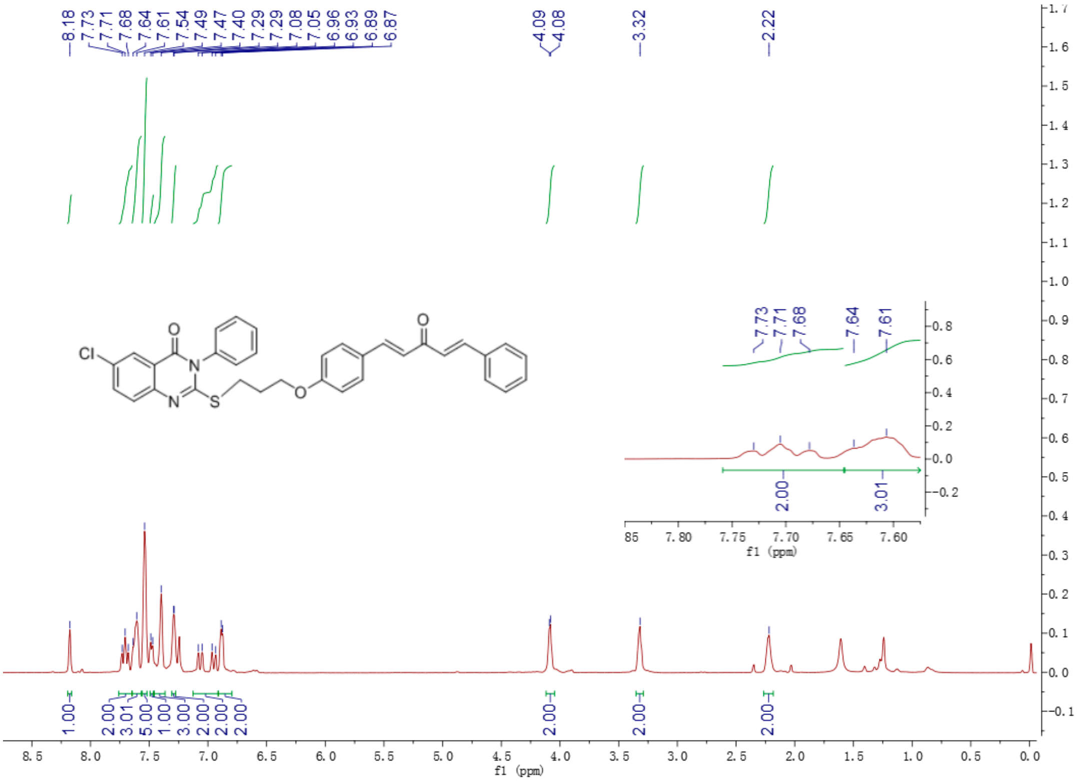

^{1}H NMR for compound **W1**

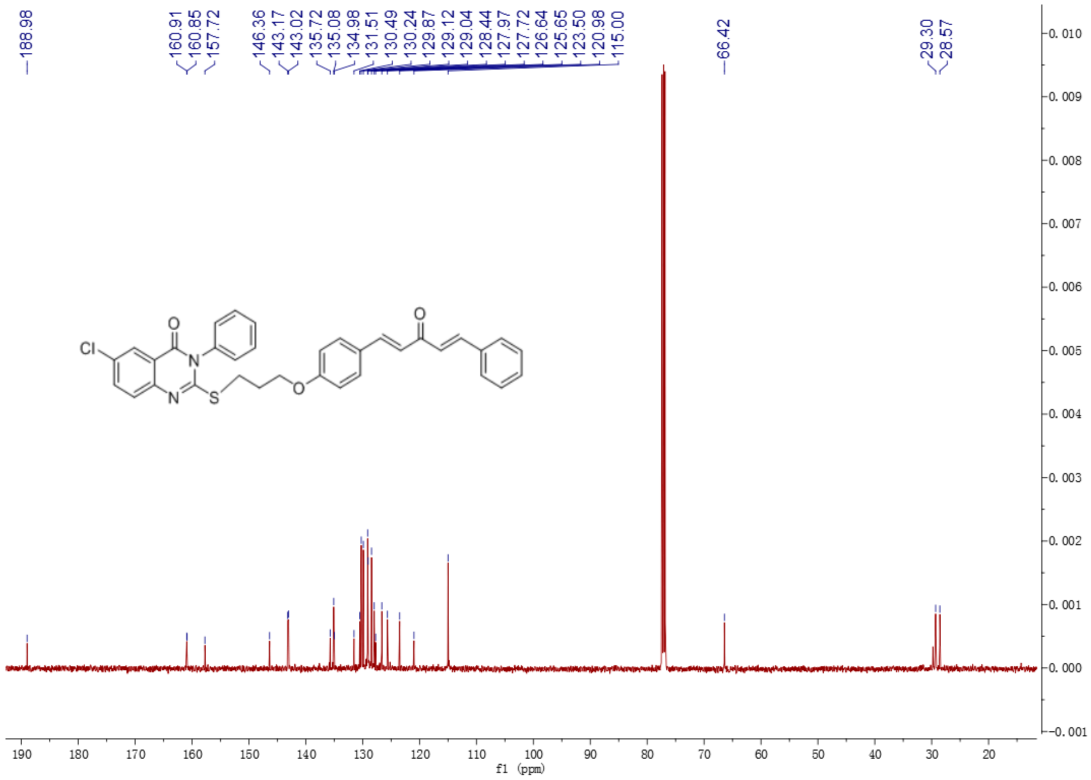

^{13}C NMR for compound **W1**

T: FTMS + p ESI Full ms [100.0000-1300.0000]

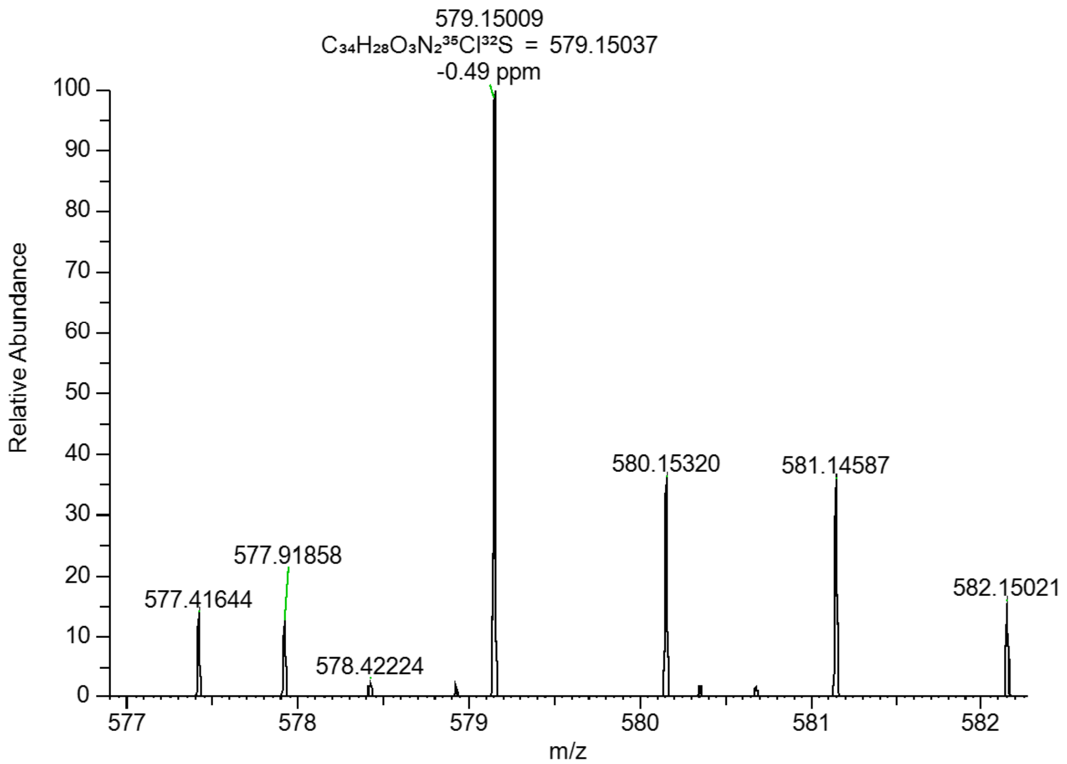

HRMS (ESI) for compound **W1**

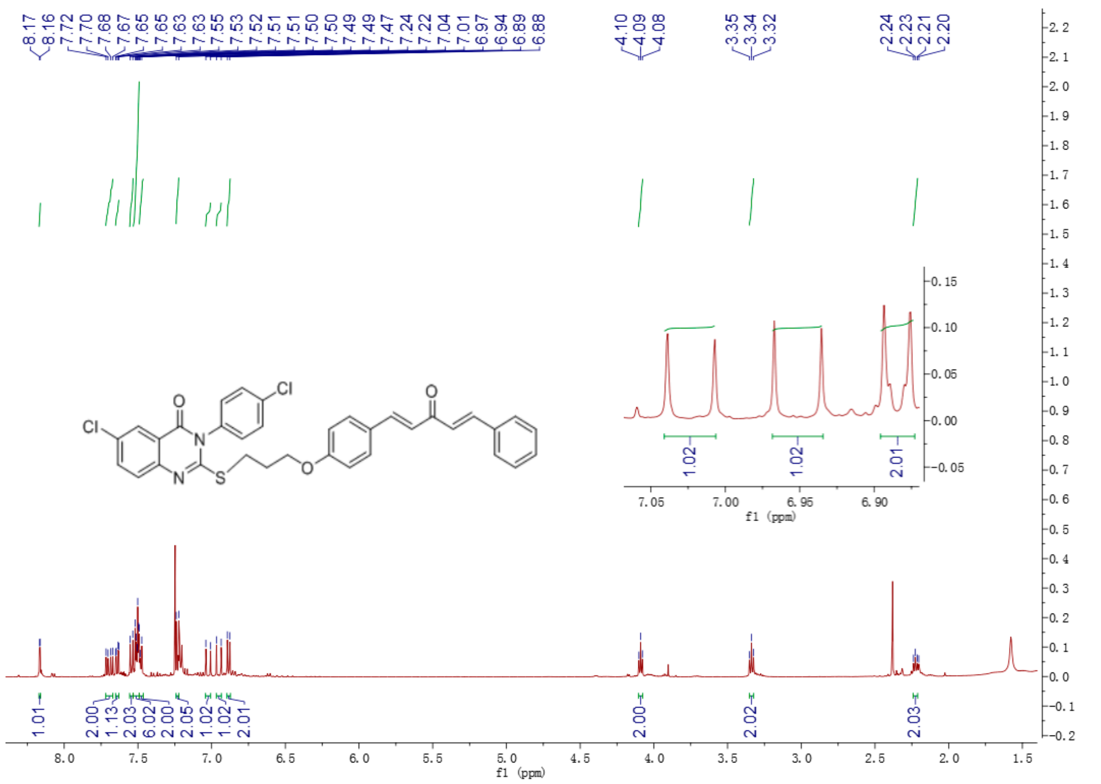

^{1}H NMR for compound **W2**

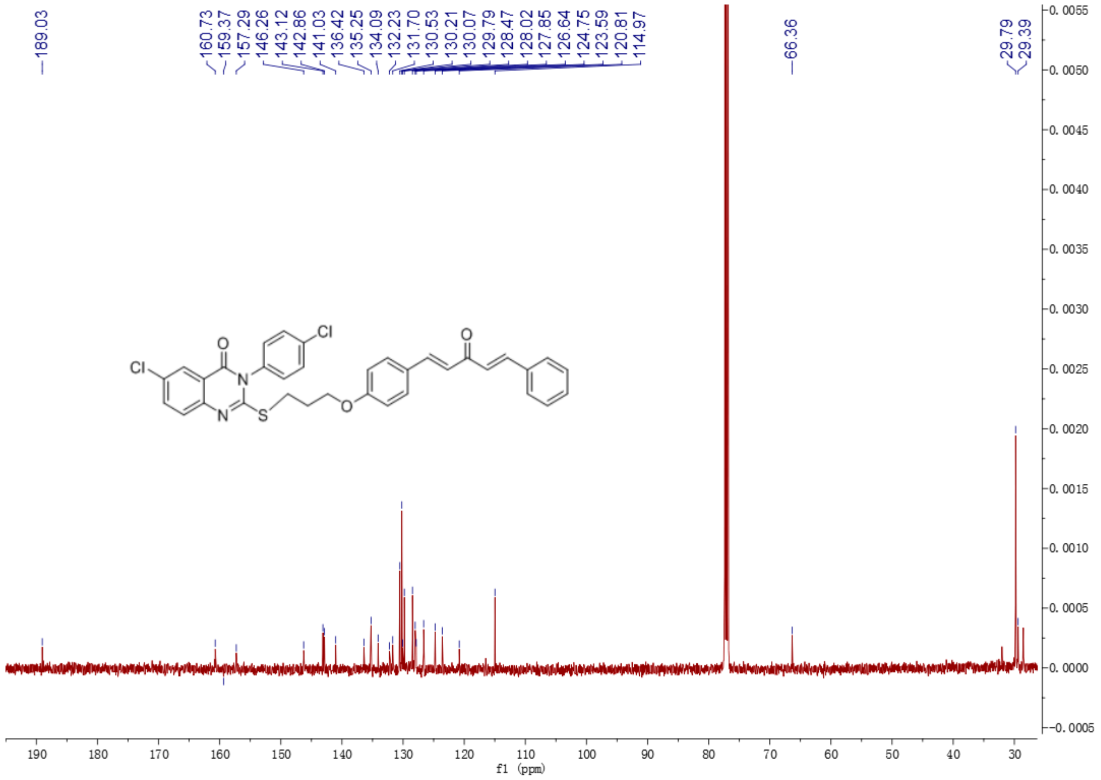

^{13}C NMR for compound **W2**

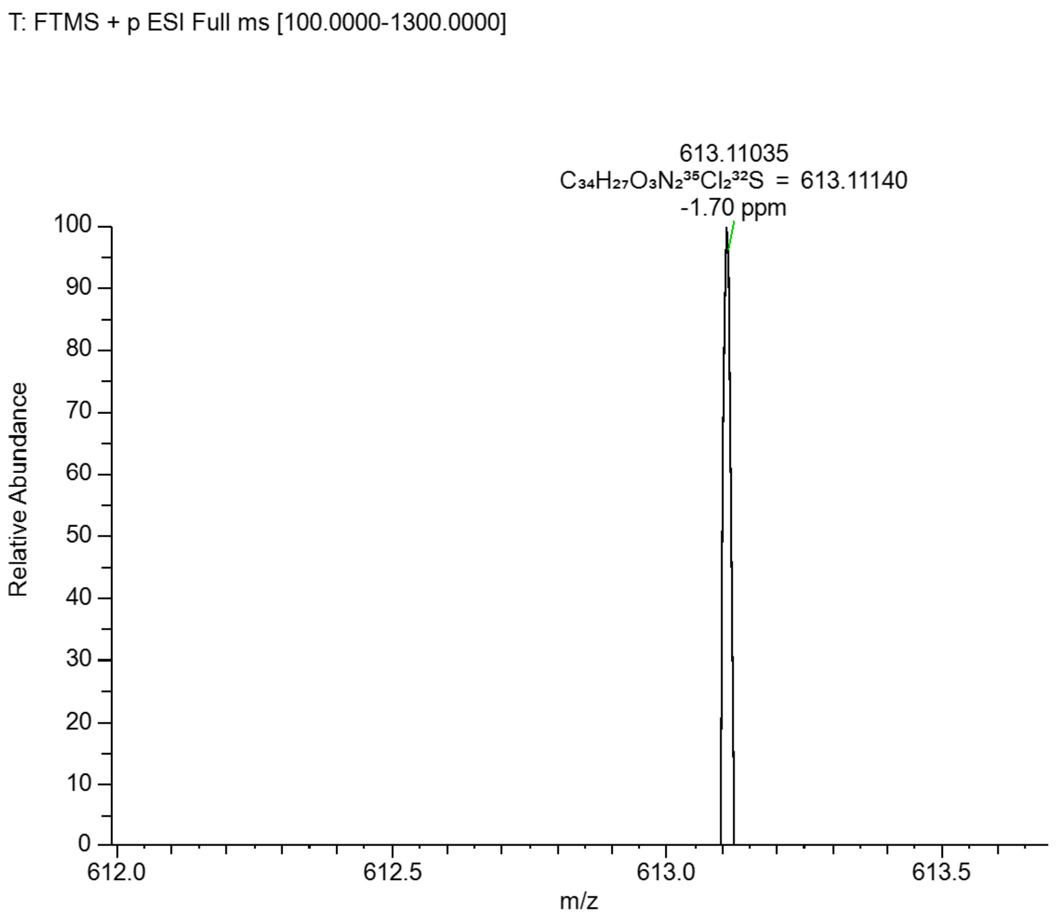

HRMS (ESI) for compound **W2**

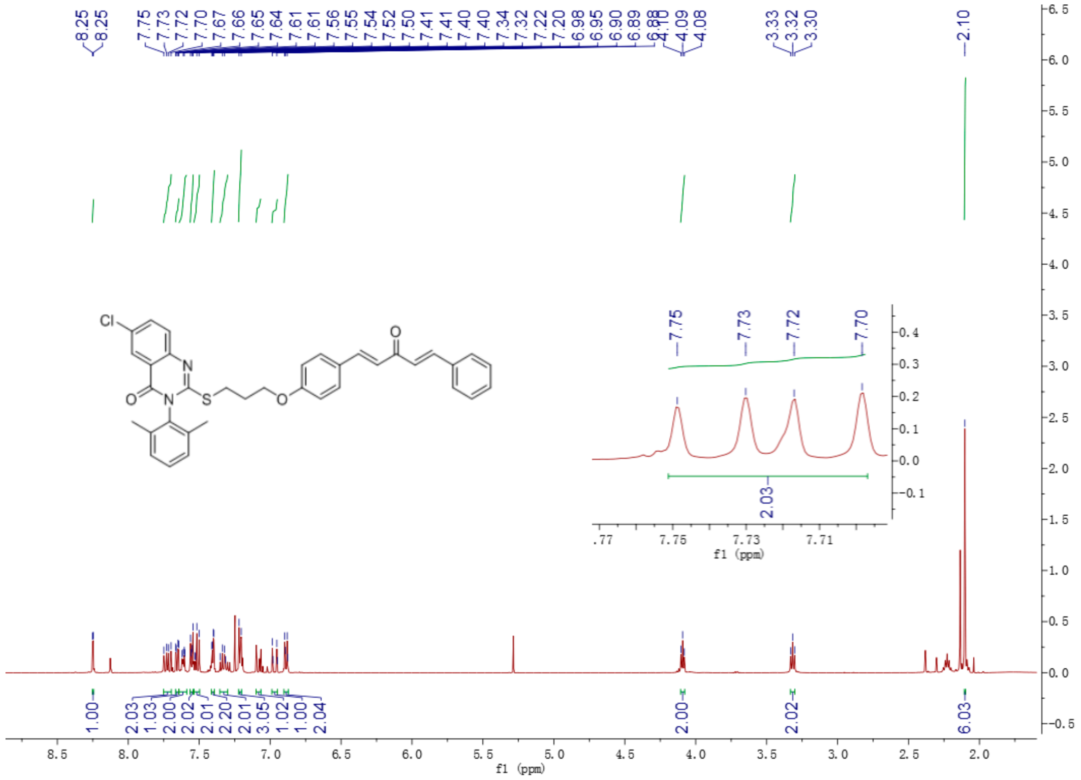

^{1}H NMR for compound **W3**

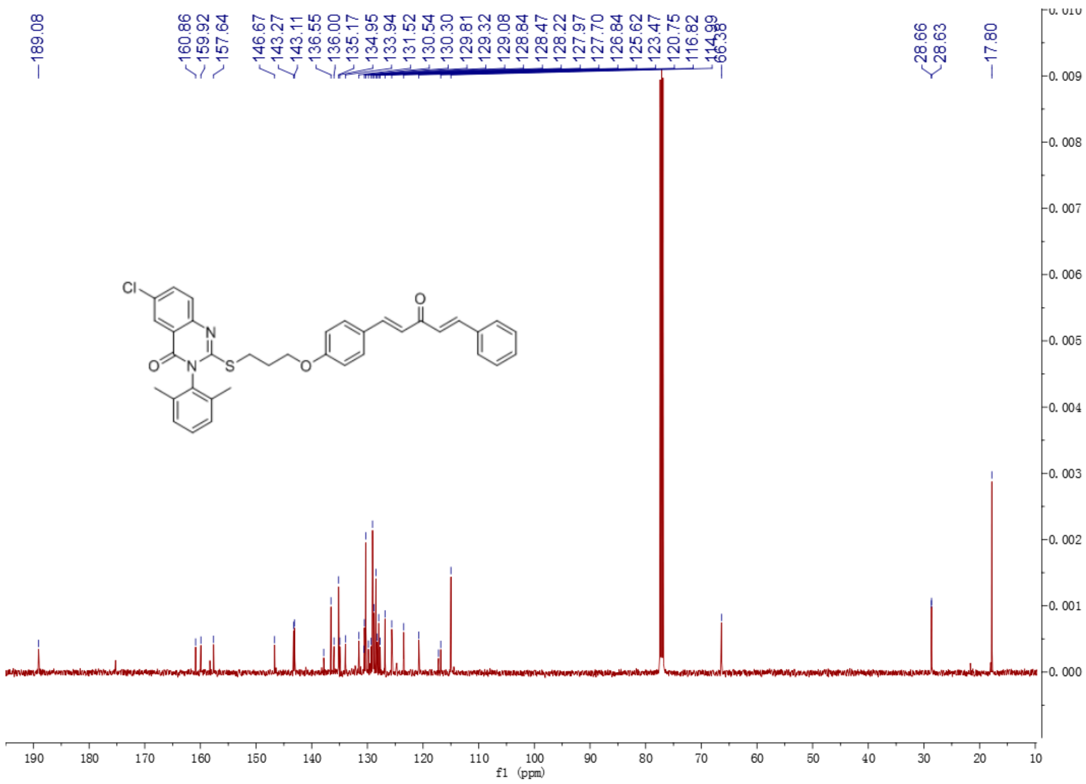

^{13}C NMR for compound **W3**

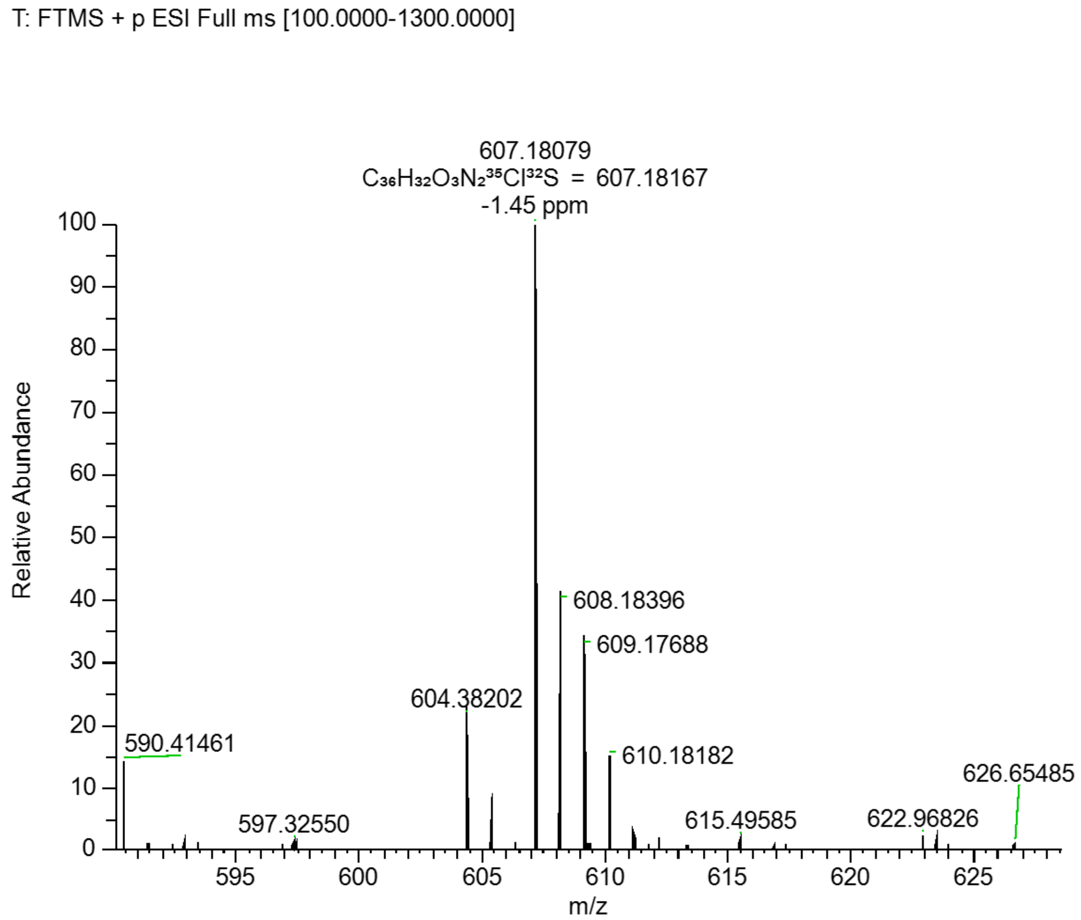

HRMS (ESI) for compound **W3**

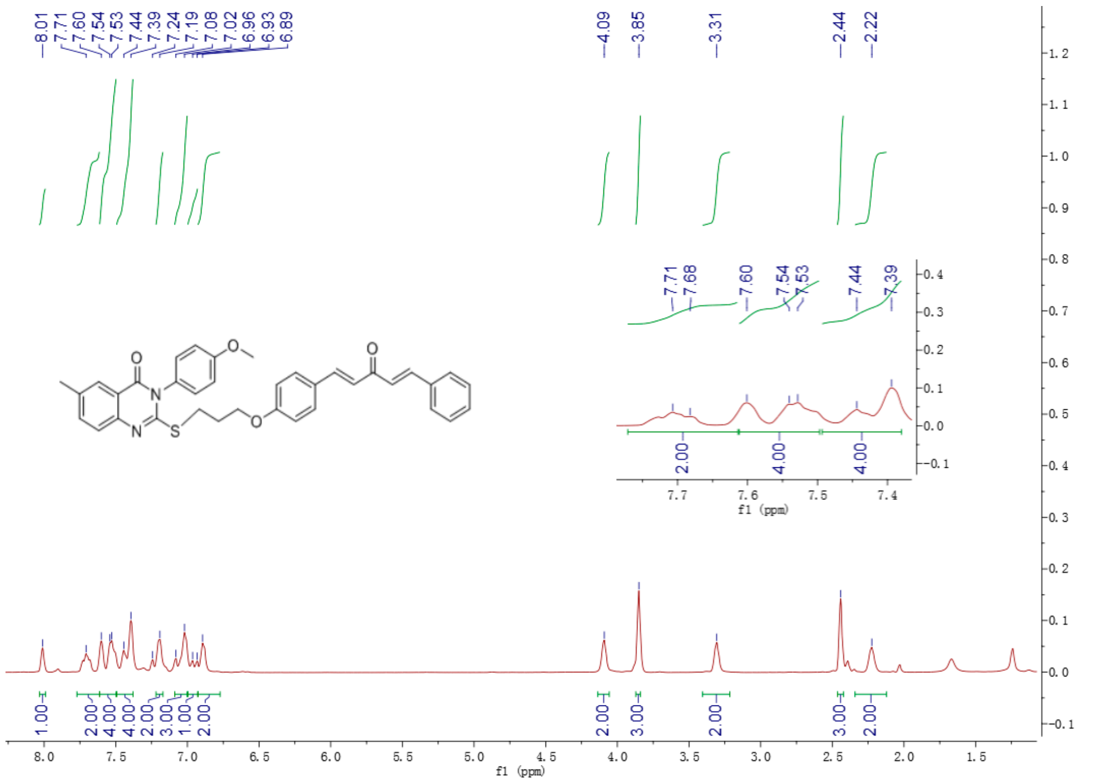

^{1}H NMR for compound **W4**

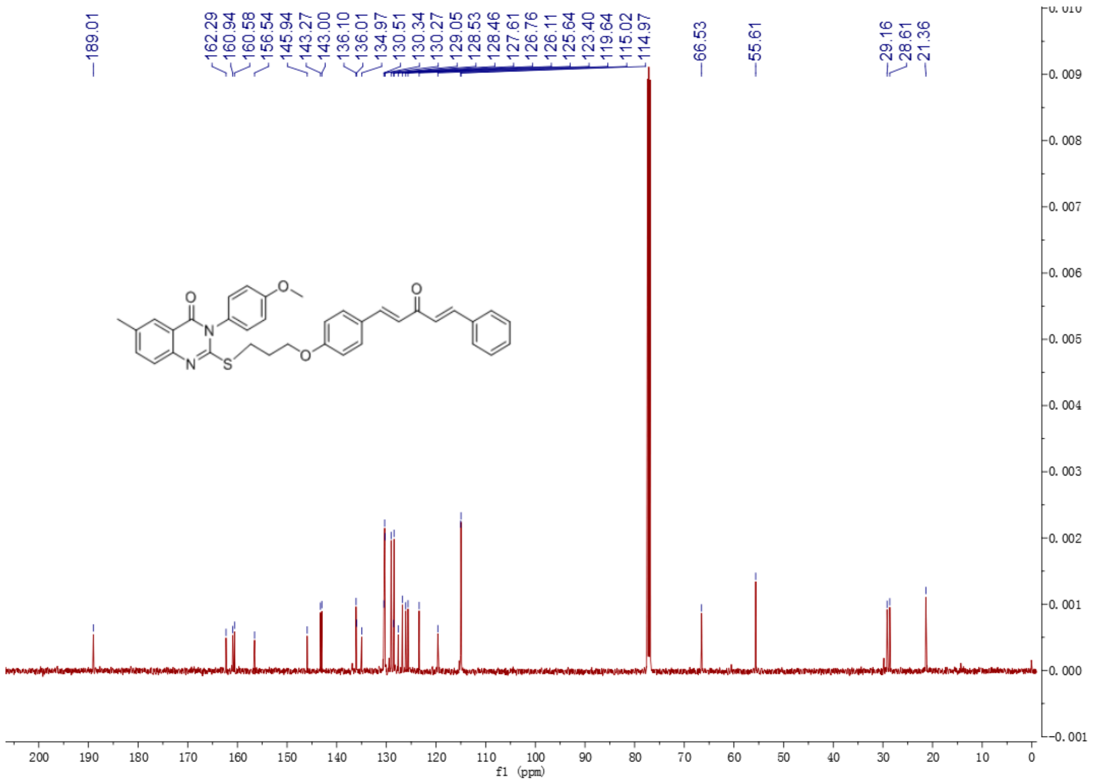

^{13}C NMR for compound **W4**

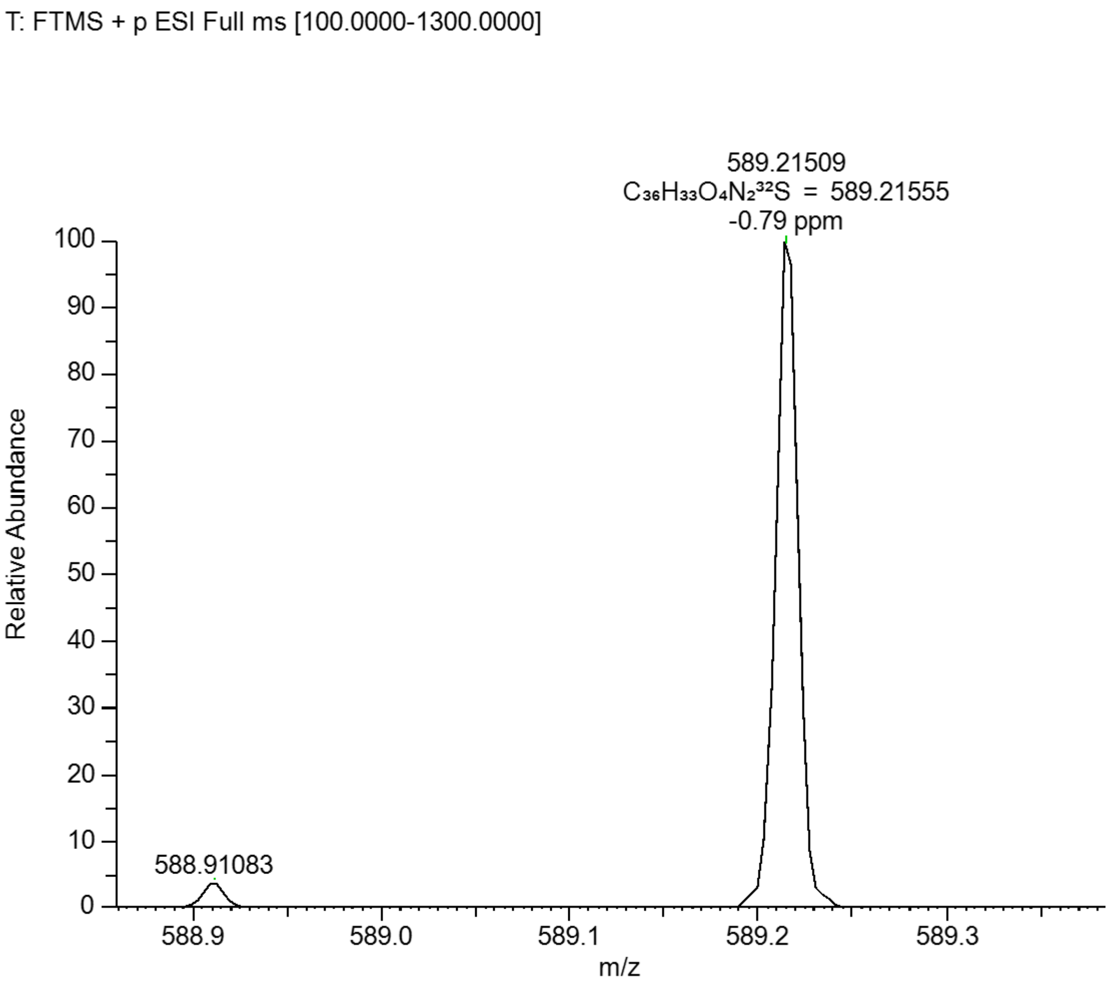

HRMS (ESI) for compound **W4**

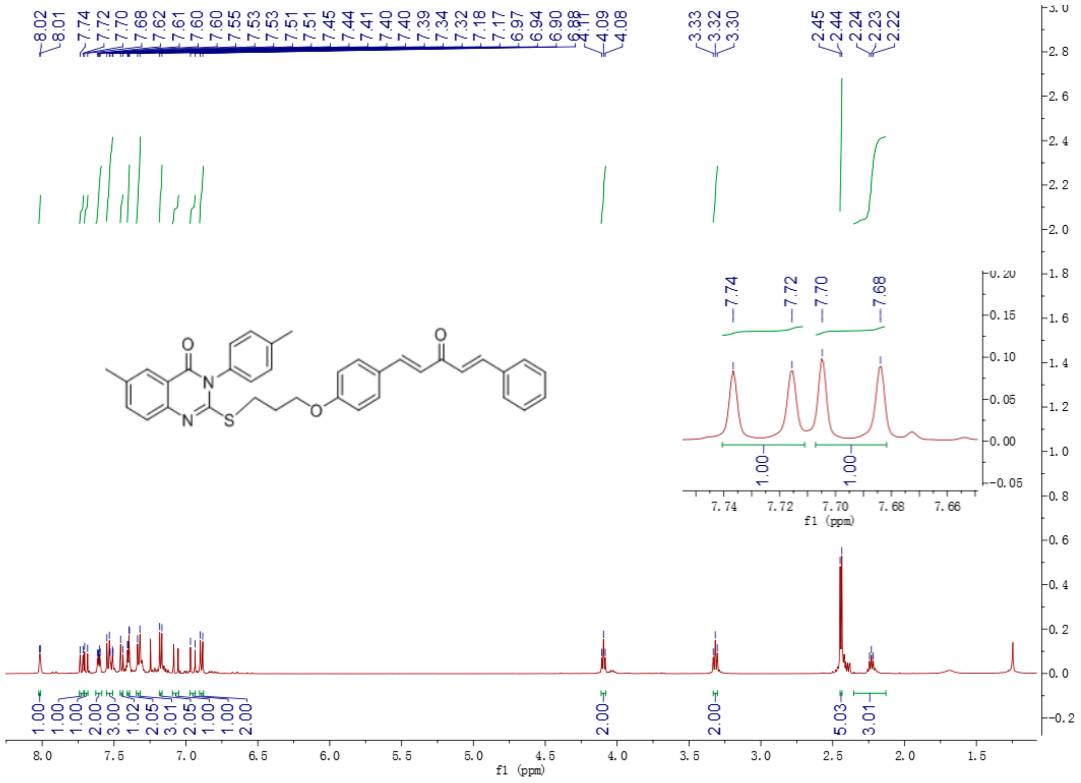

^{1}H NMR for compound **W5**

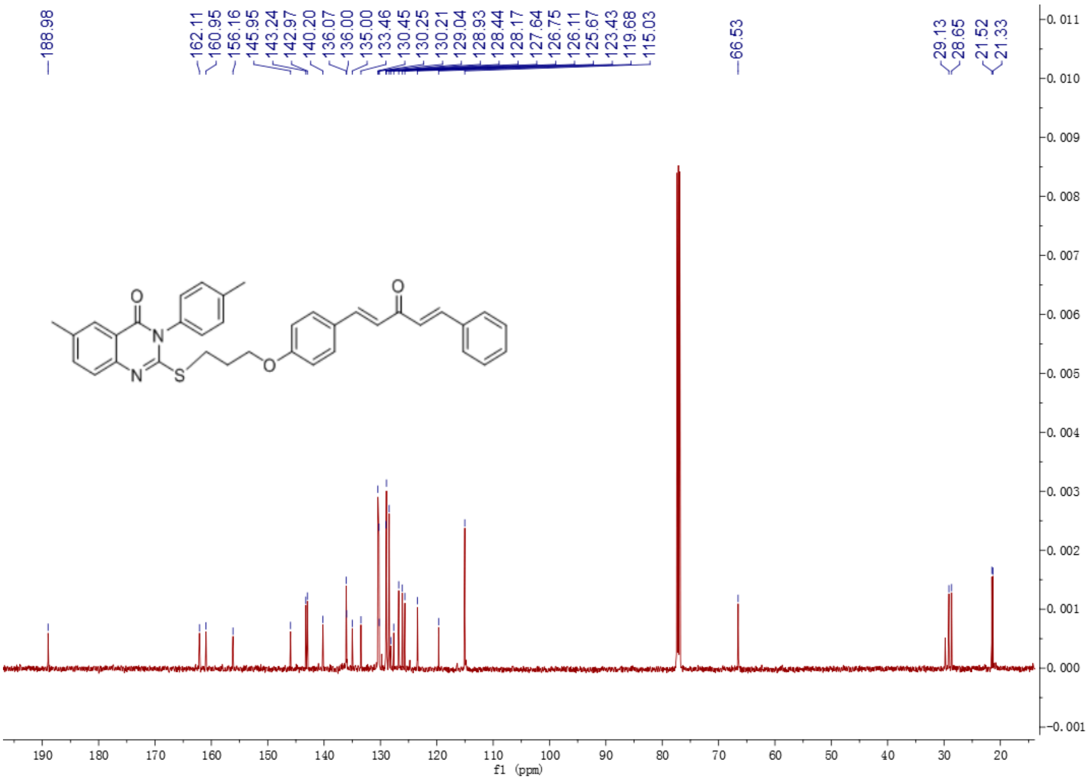

^{13}C NMR for compound **W5**

T: FTMS + p ESI Full ms [100.0000-1300.0000]

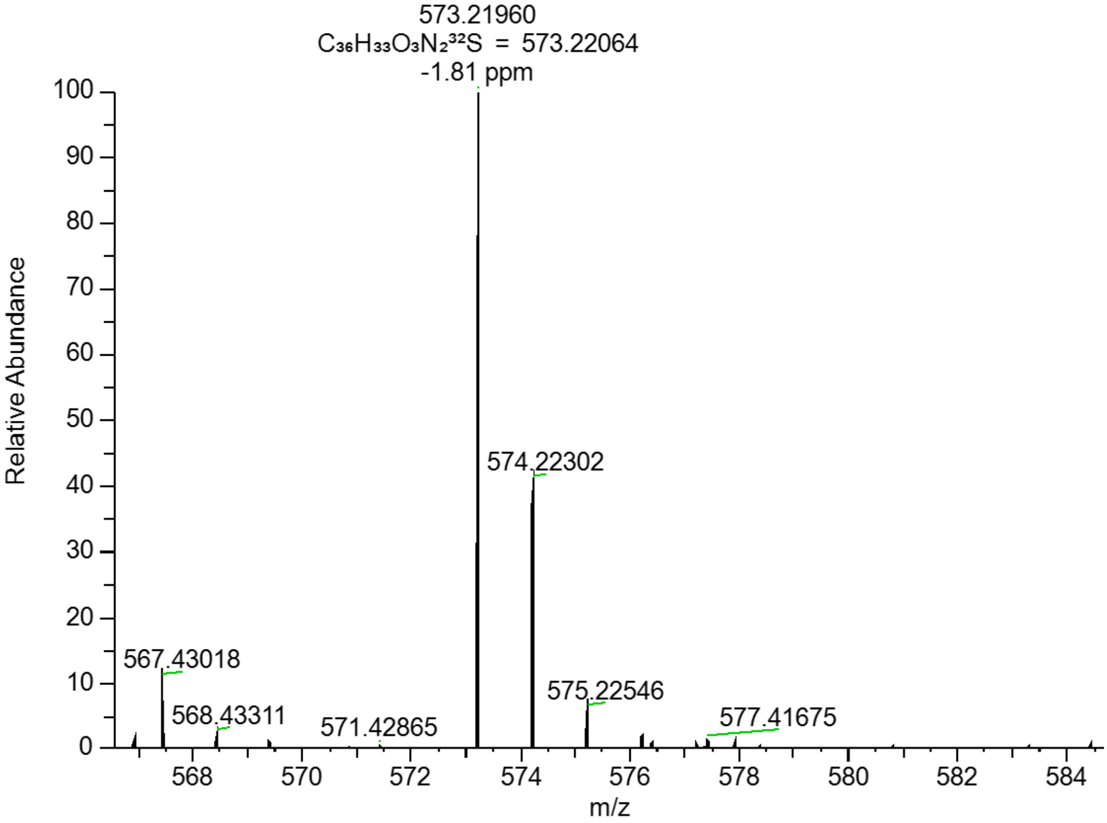

S

HRMS (ESI) for compound **W5**

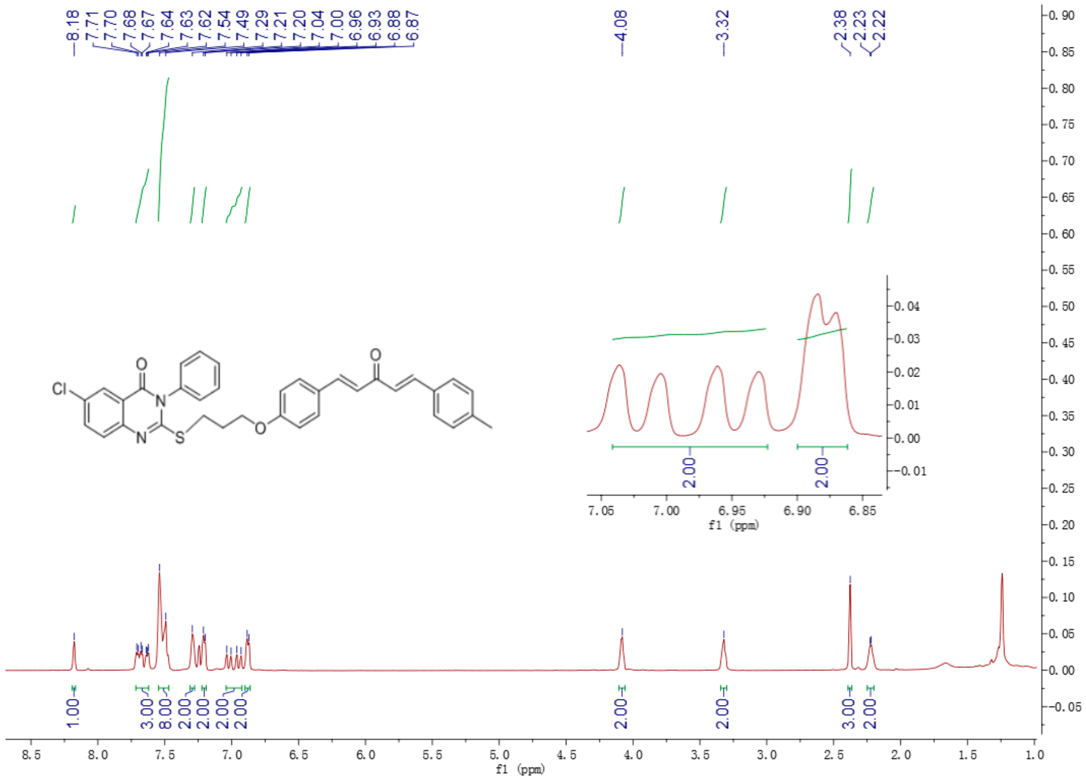

^{1}H NMR for compound **W6**

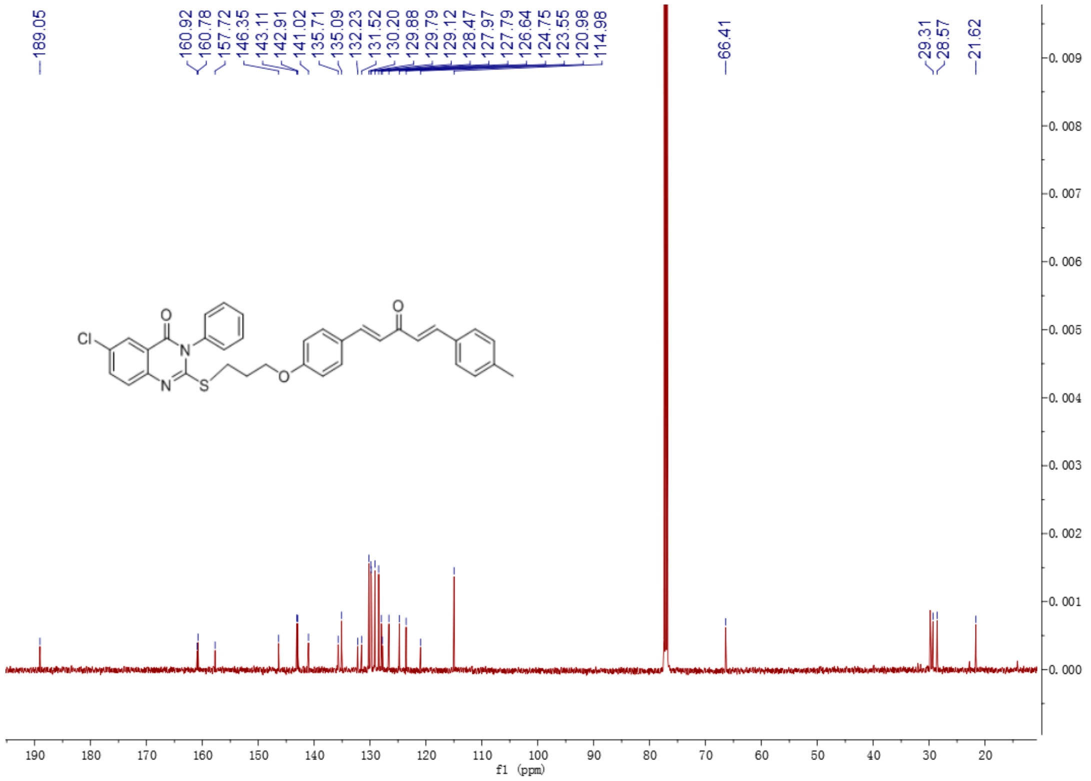

^{13}C NMR for compound **W6**

T: FTMS + p ESI Full ms [100.0000-1300.0000]

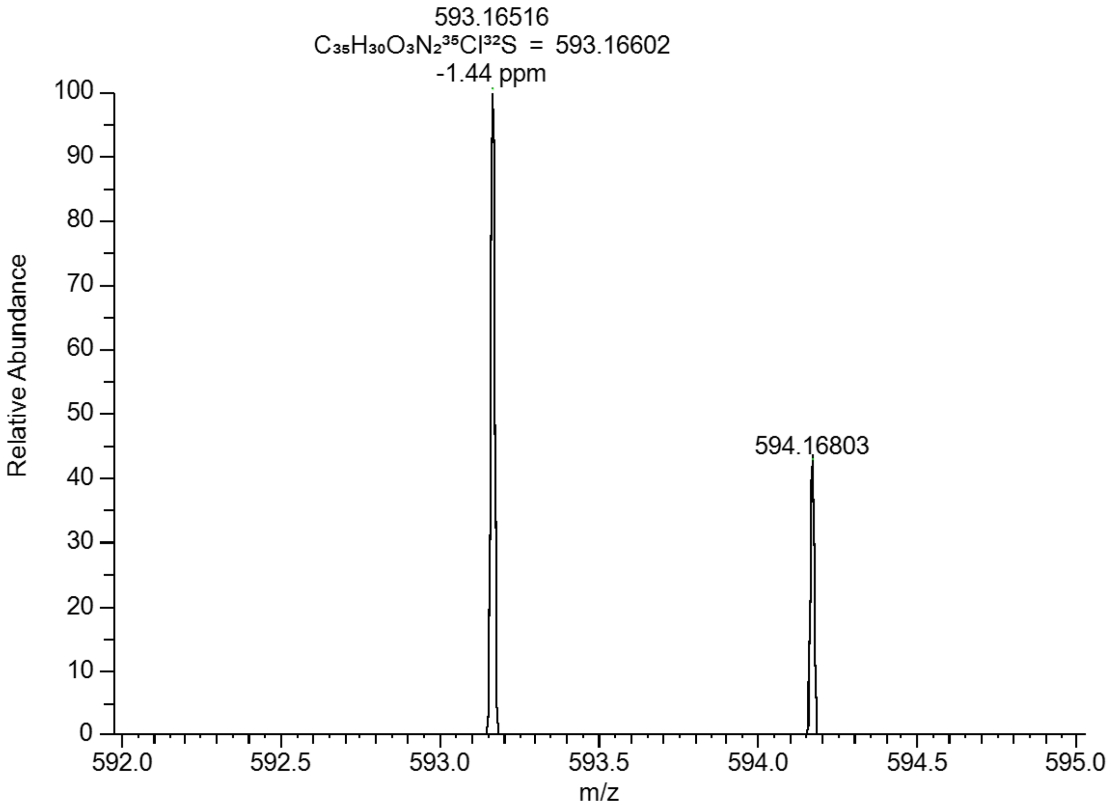

HRMS (ESI) for compound **W6**

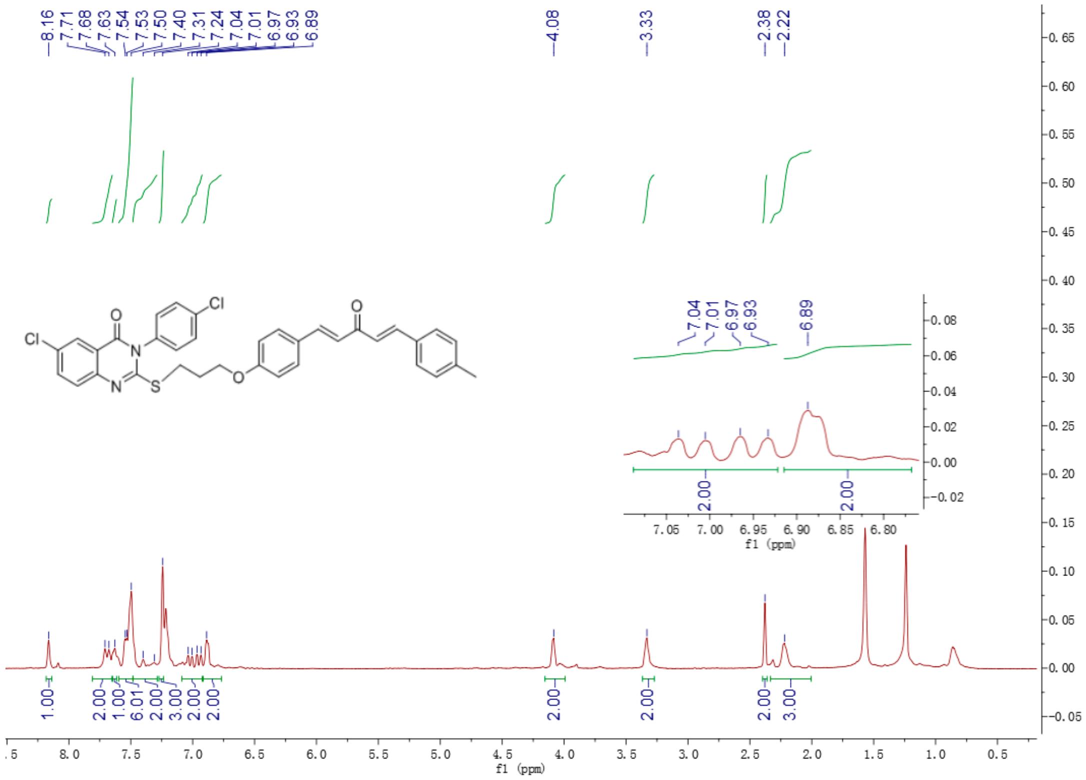

^{1}H NMR for compound **W7**

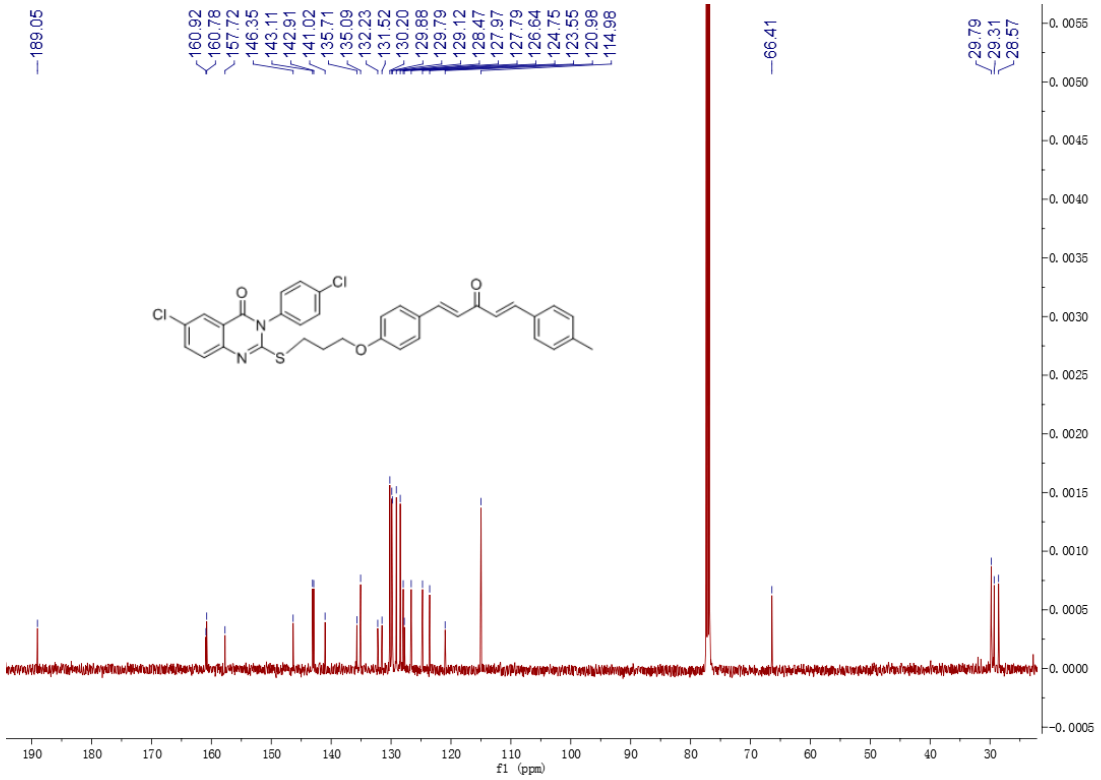

^{13}C NMR for compound **W7**

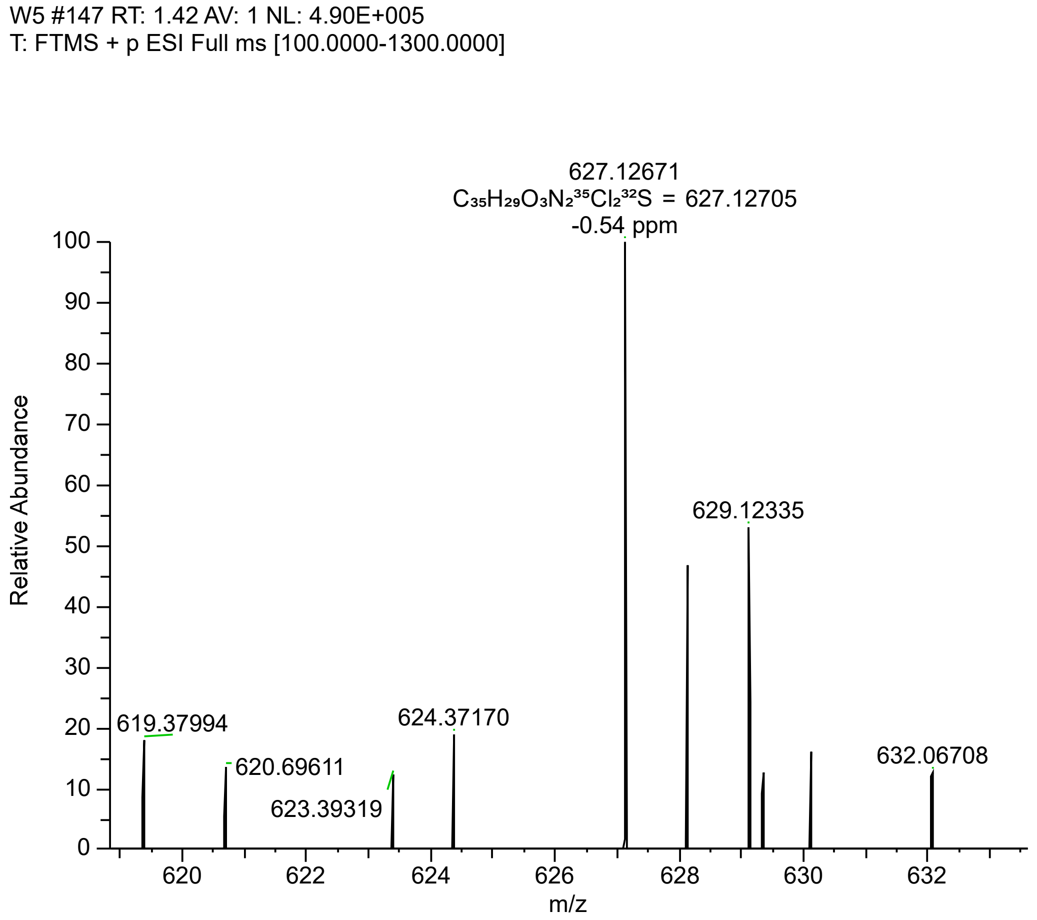

HRMS (ESI) for compound **W7**

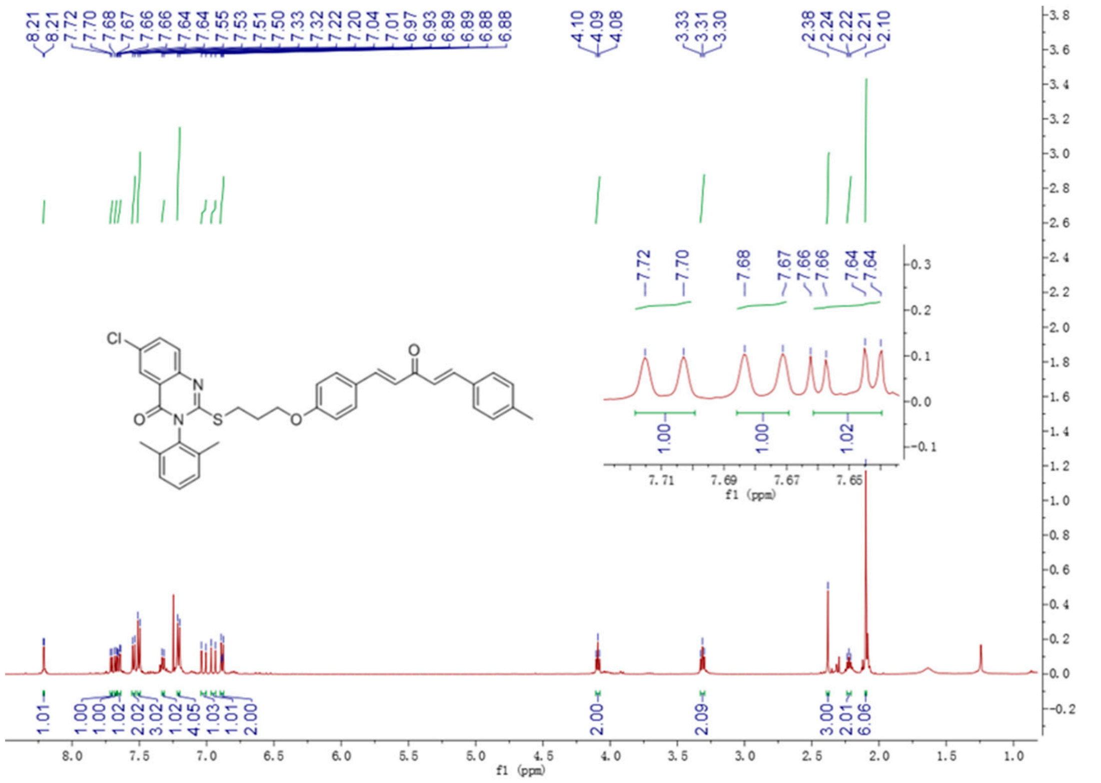

^{1}H NMR for compound **W8**

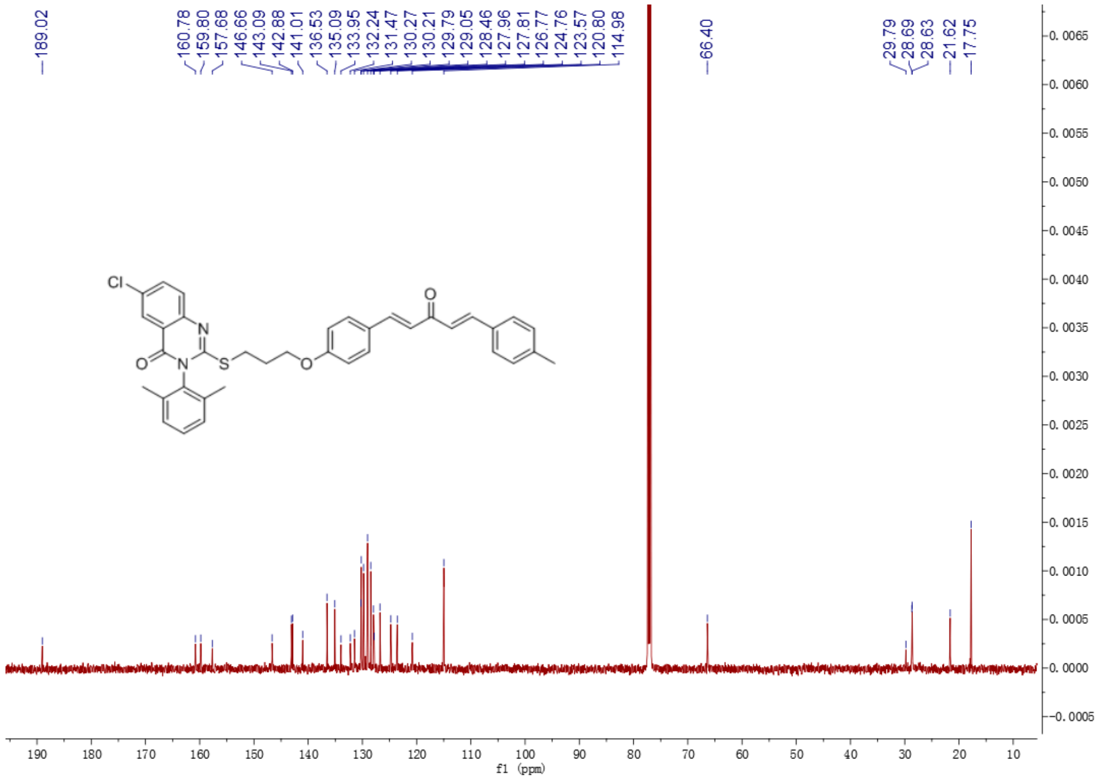

^{13}C NMR for compound **W8**

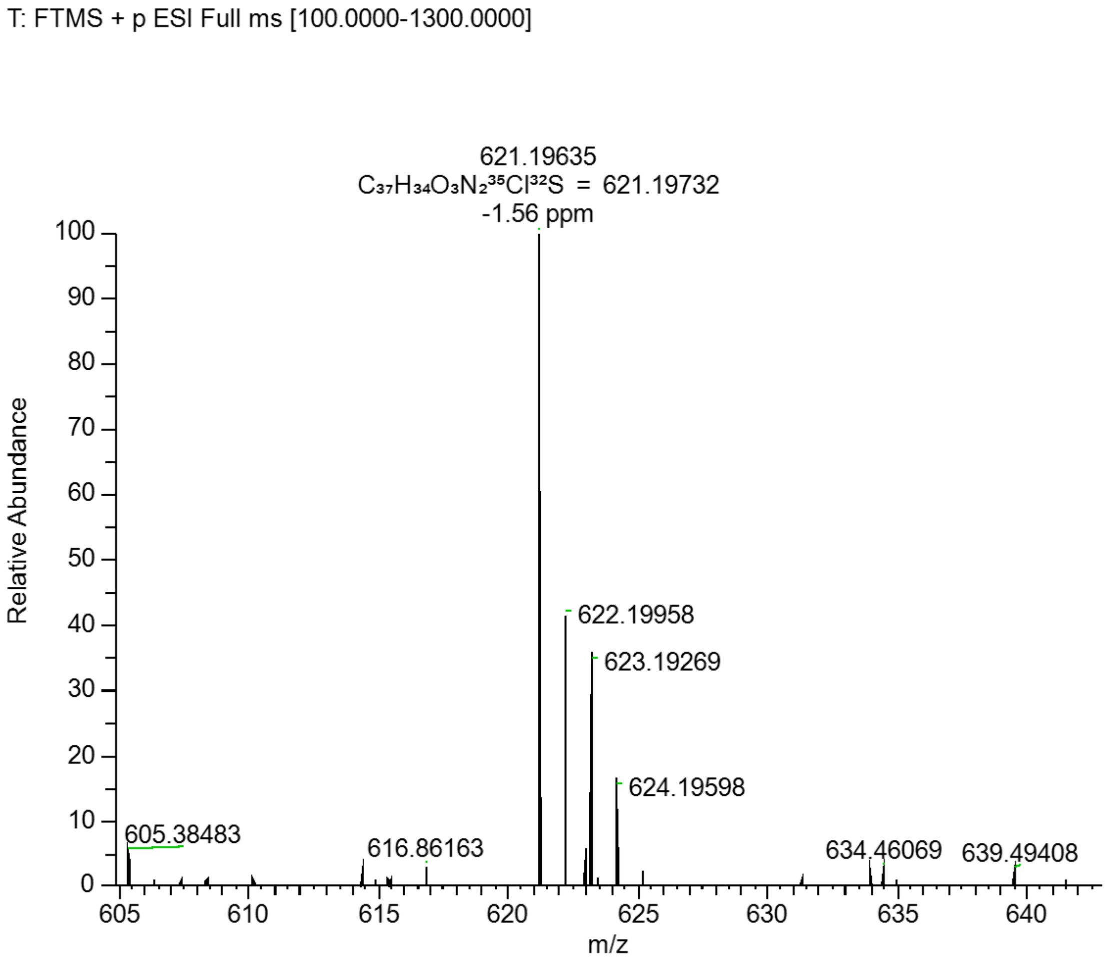

HRMS (ESI) for compound **W8**

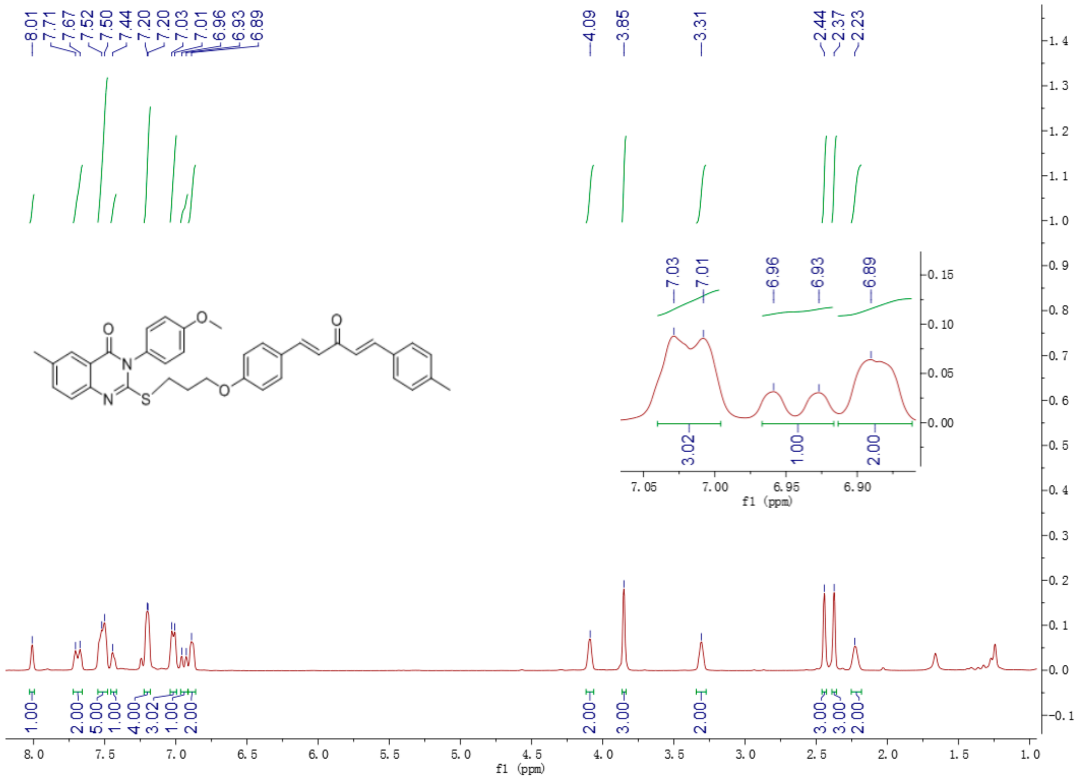

^{1}H NMR for compound **W9**

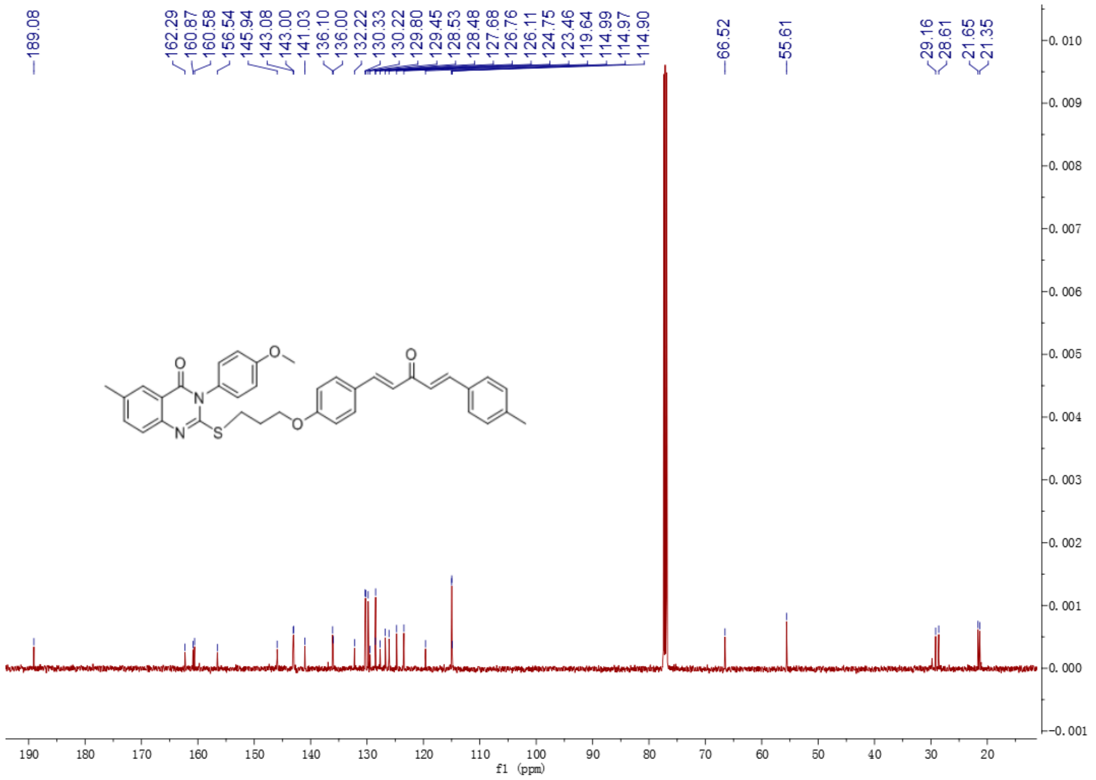

^{13}C NMR for compound **W9**

T: FTMS + p ESI Full ms [100.0000-1300.0000]

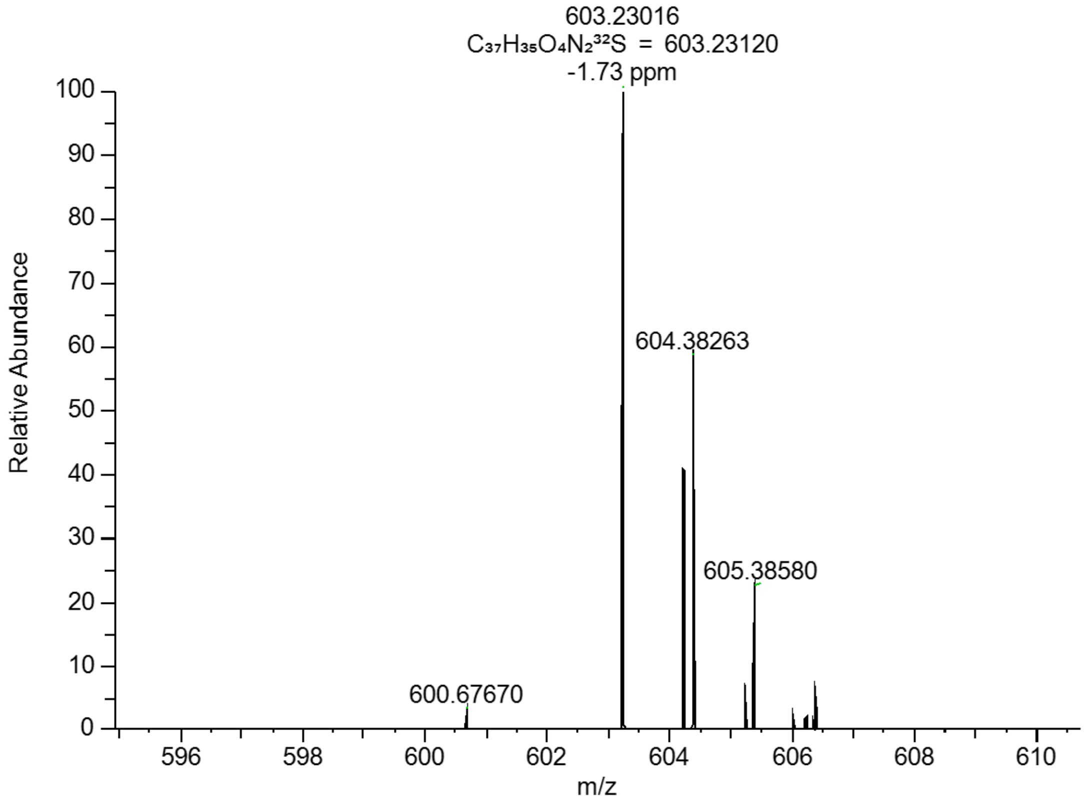

HRMS (ESI) for compound **W9**

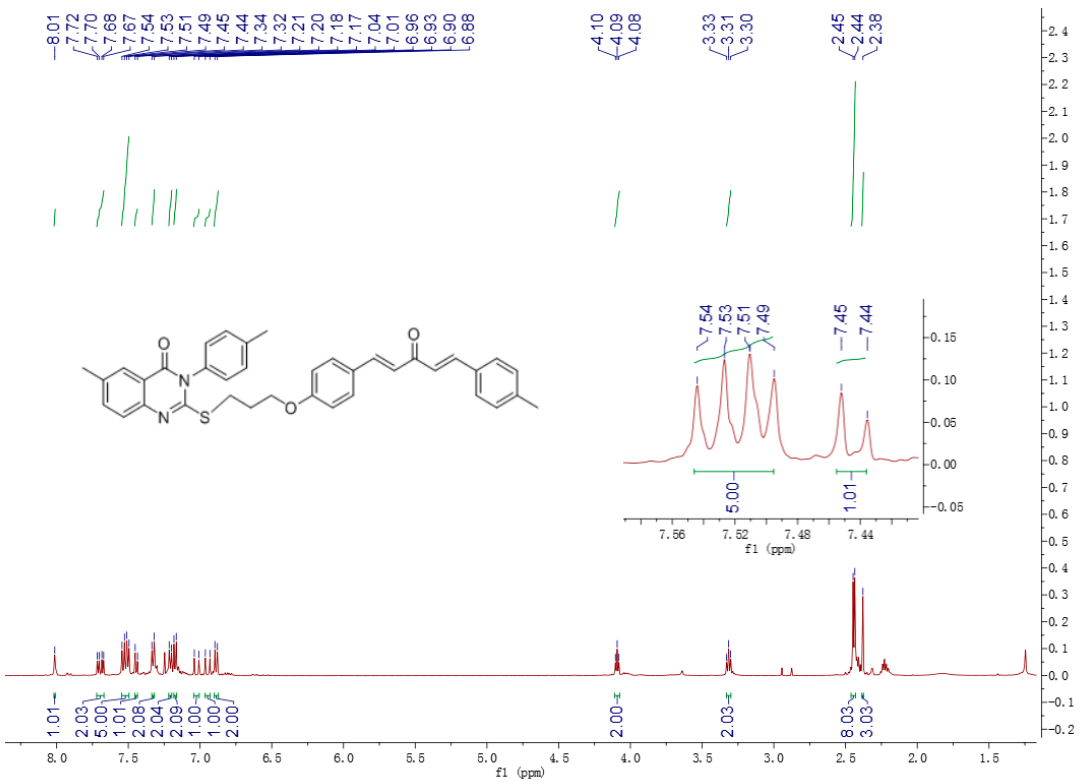

^{1}H NMR for compound **W10**

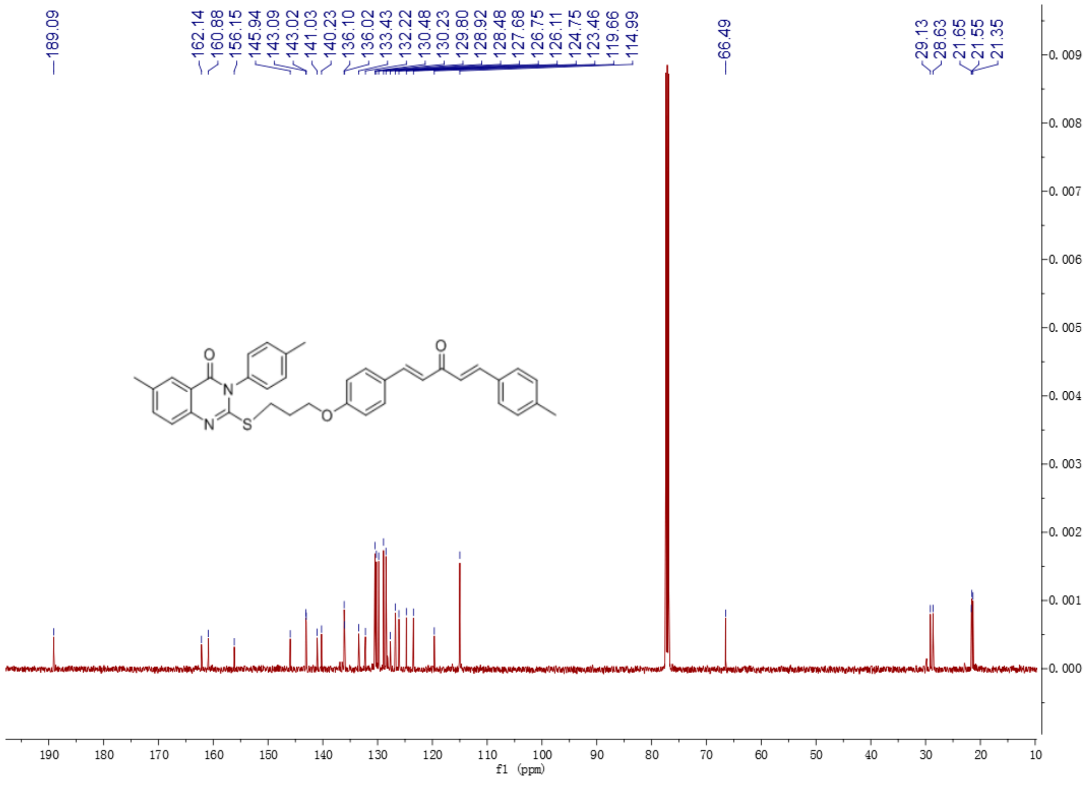

^{13}C NMR for compound **W10**

T: FTMS + p ESI Full ms [100.0000-1300.0000]

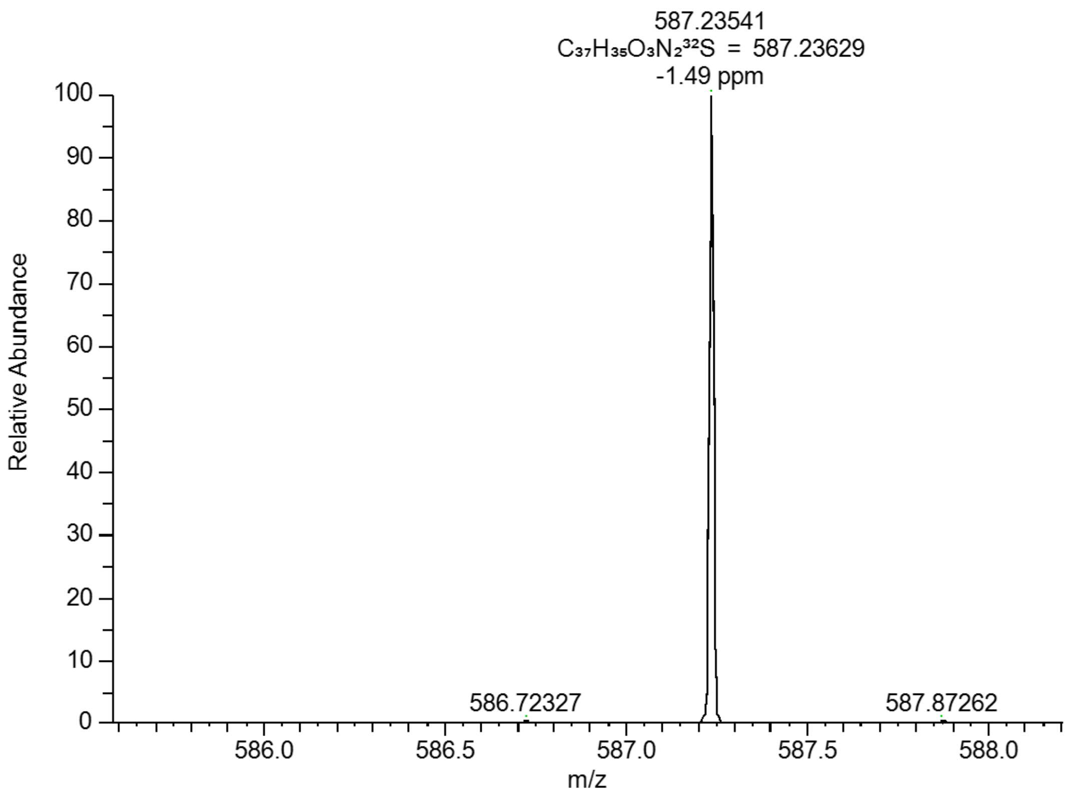

HRMS (ESI) for compound **W10**

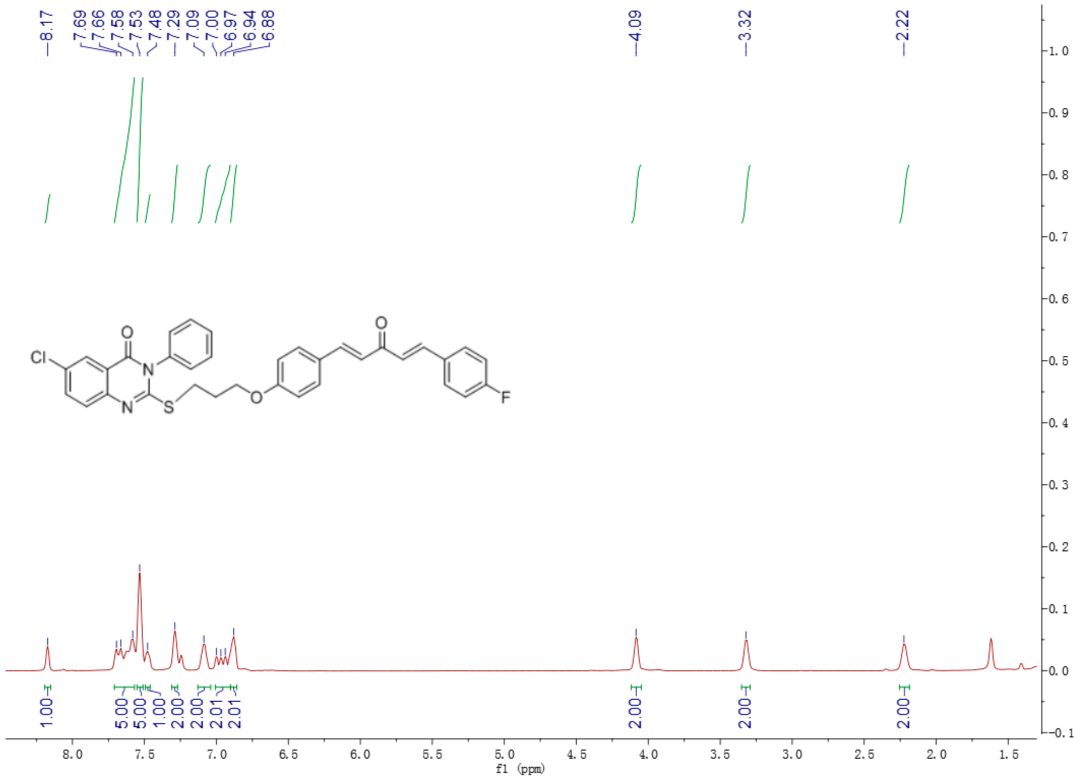

^{1}H NMR for compound **W11**

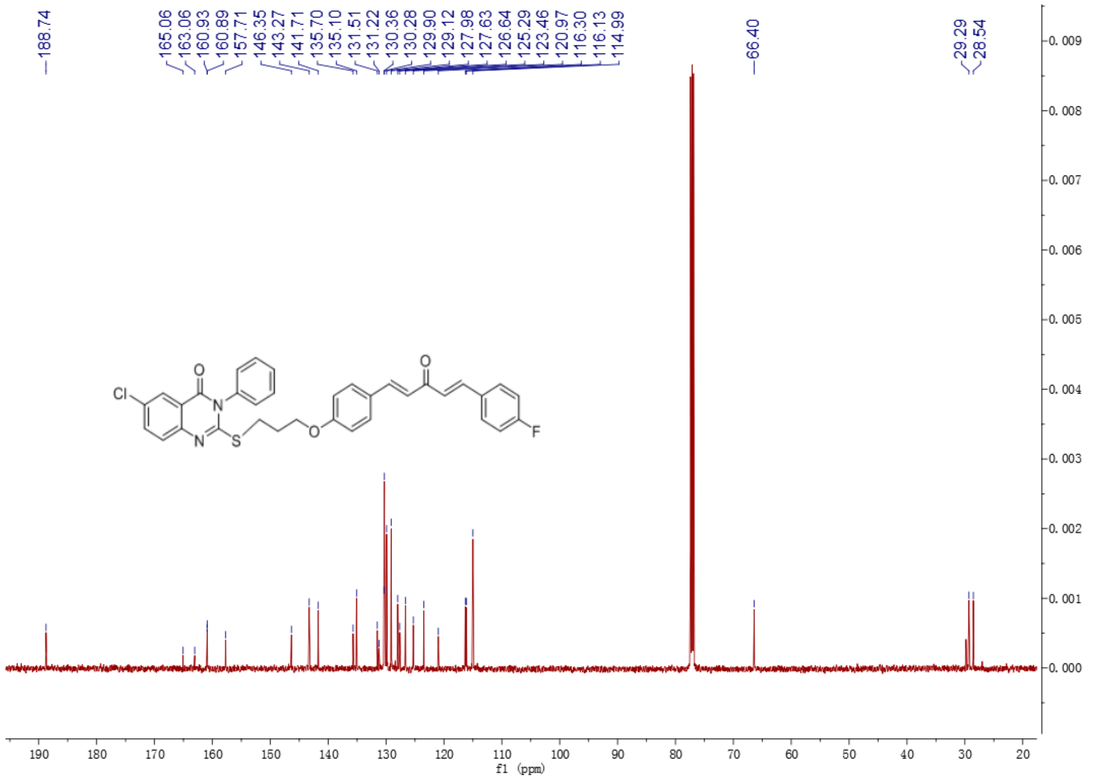

^{13}C NMR for compound **W11**

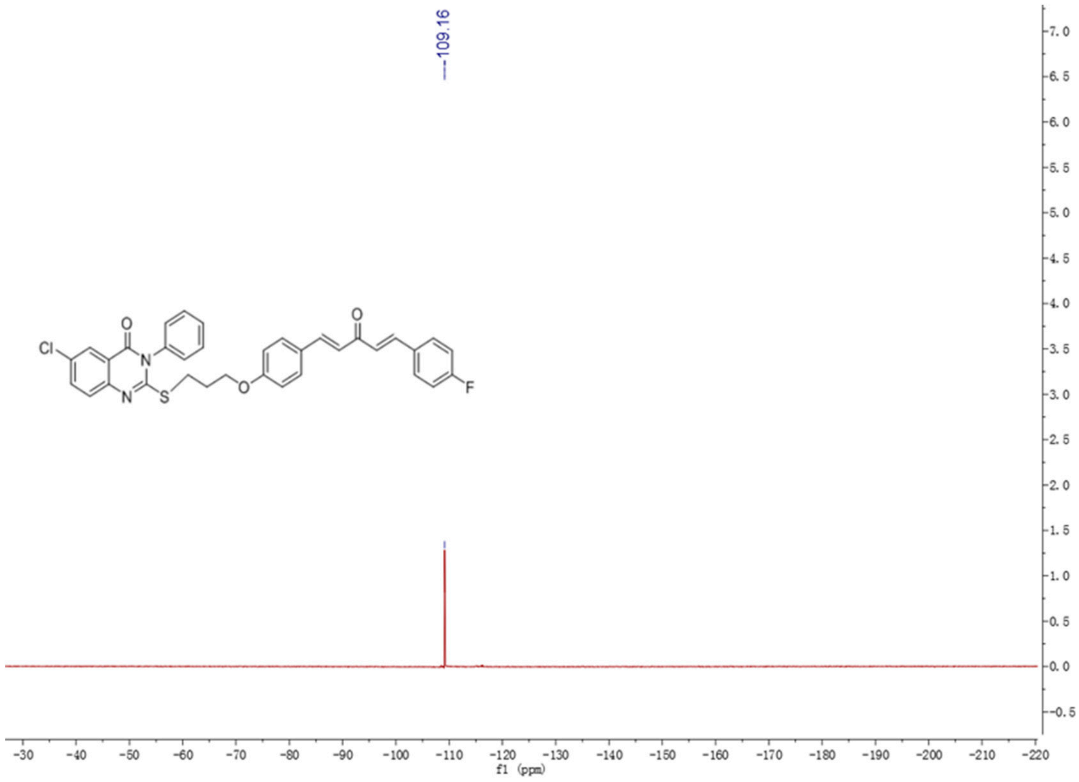

^{19}F NMR for compound **W11**

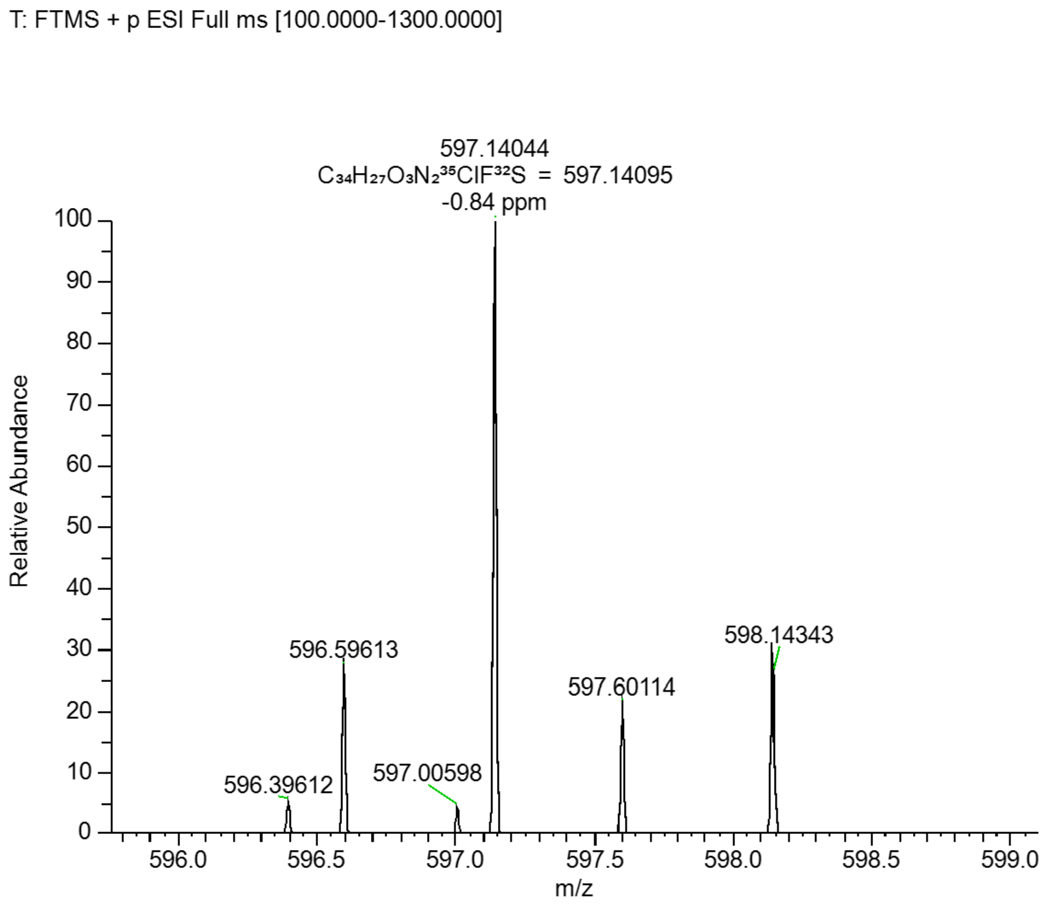

HRMS (ESI) for compound **W11**

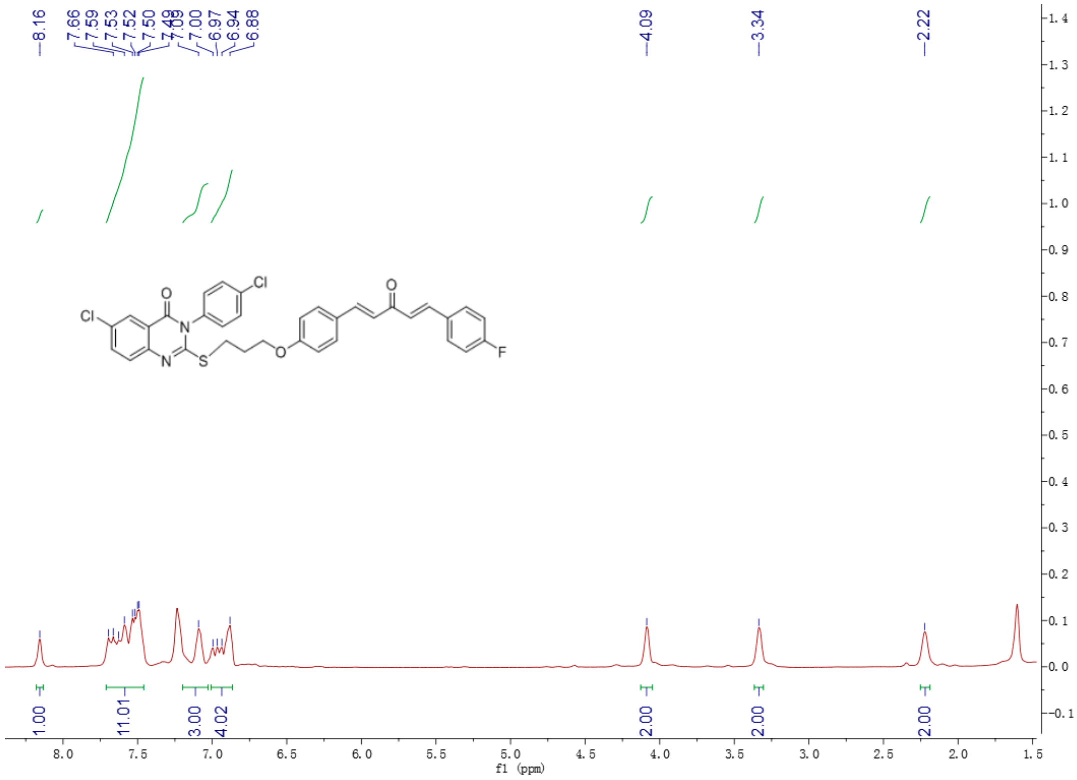

^{1}H NMR for compound **W12**

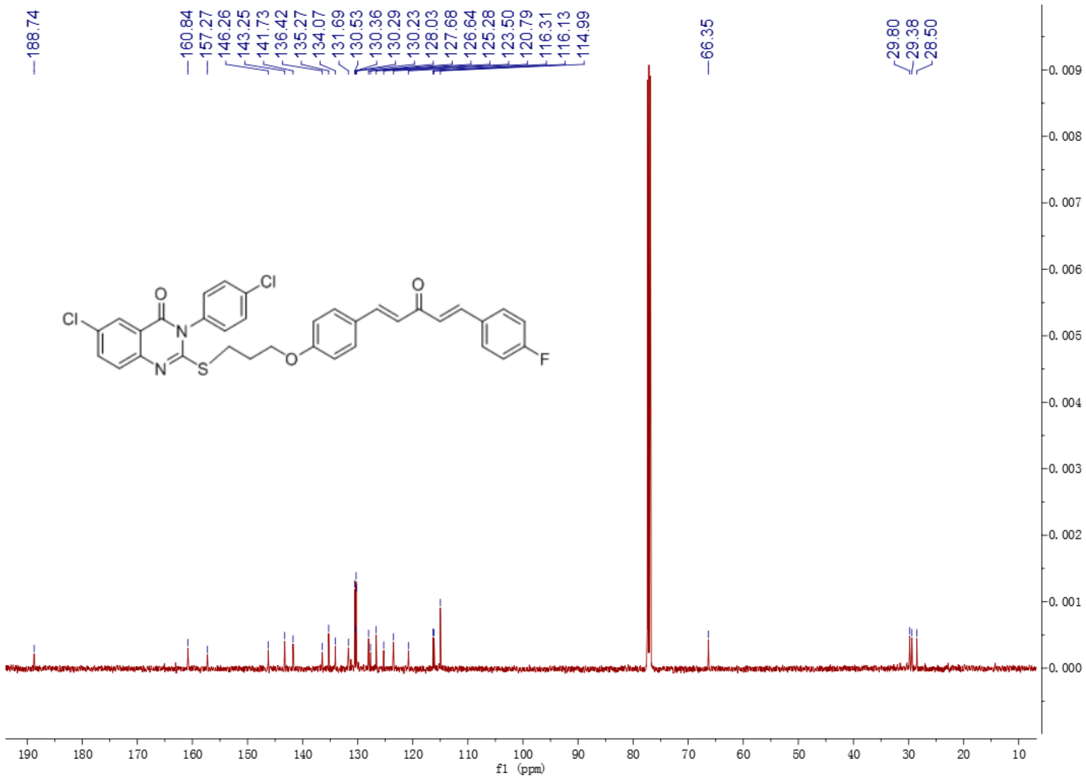

^{13}C NMR for compound **W12**

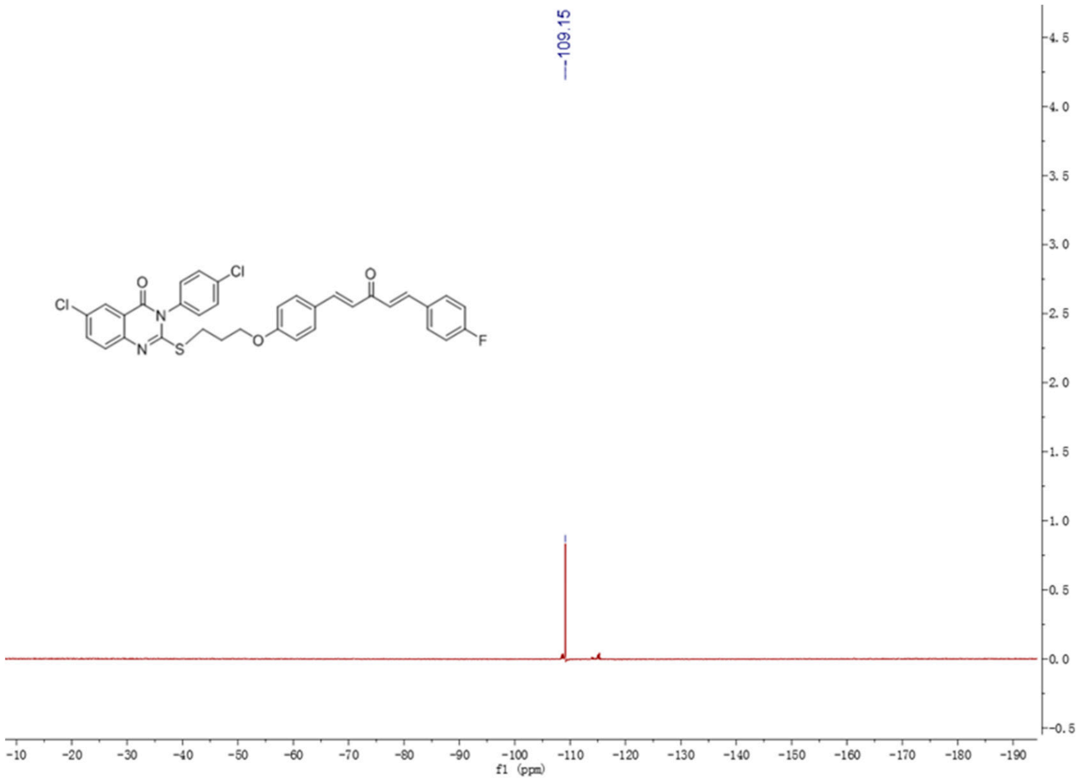

^{19}F NMR for compound **W12**

T: FTMS + p ESI Full ms [100.0000-1300.0000]

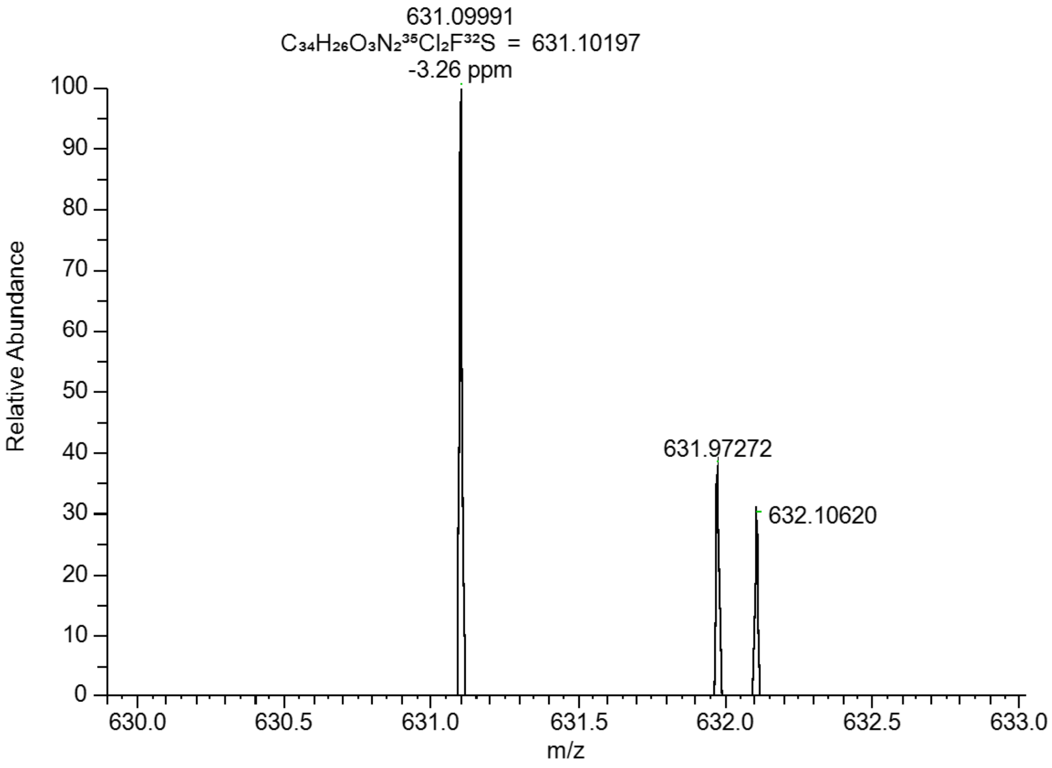

HRMS (ESI) for compound **W12**

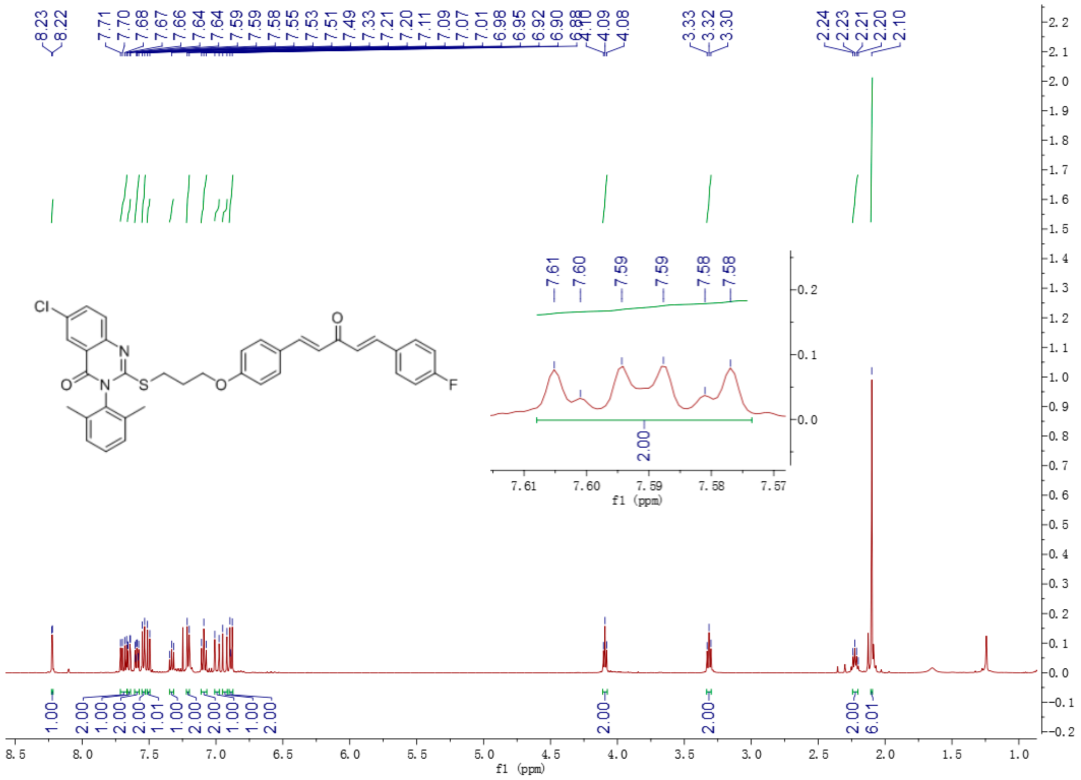

^{1}H NMR for compound **W13**

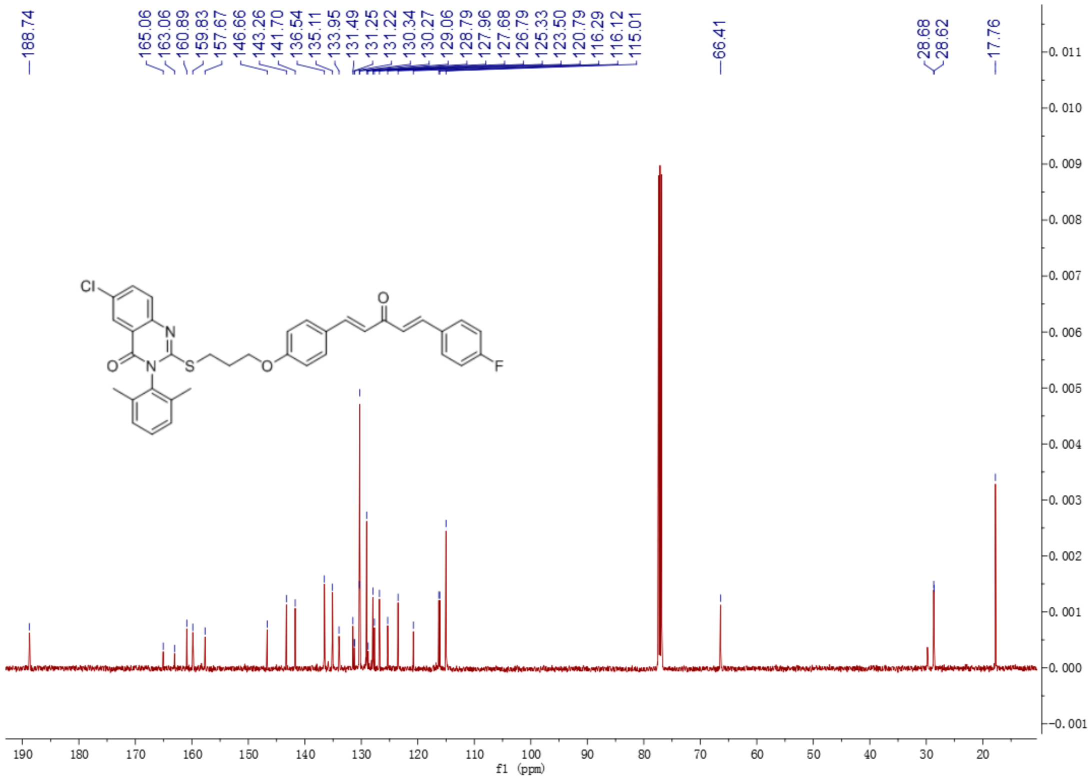

^{13}C NMR for compound **W13**

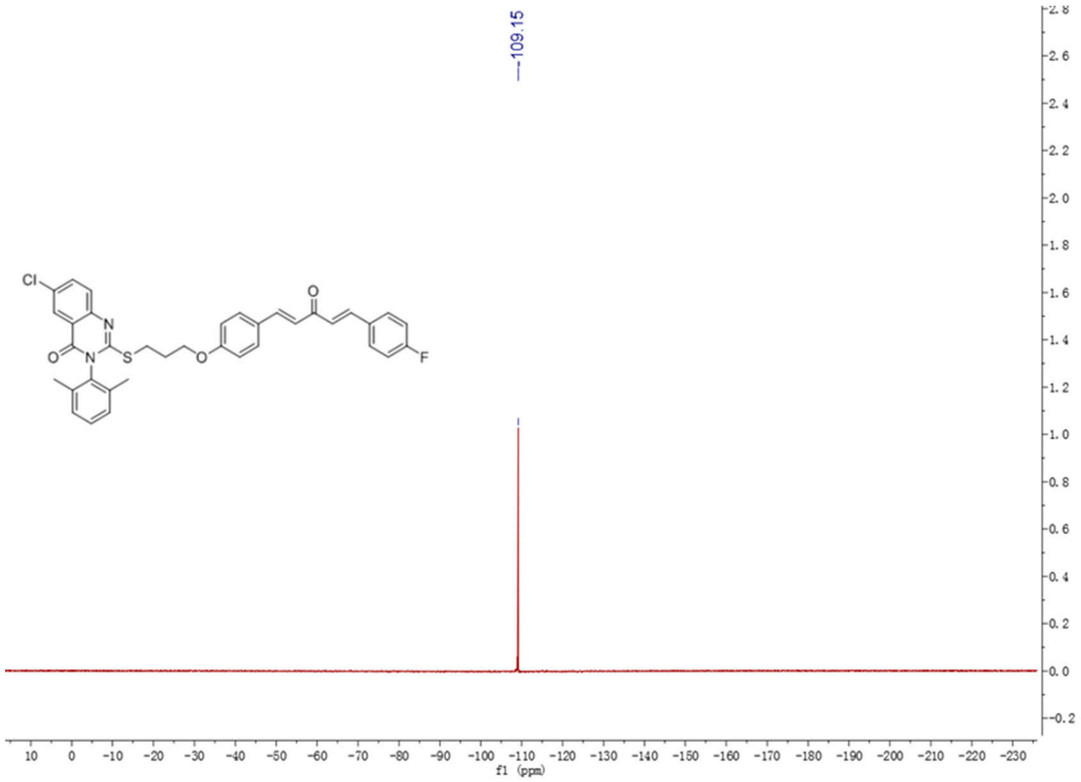

^{19}F NMR for compound **W13**

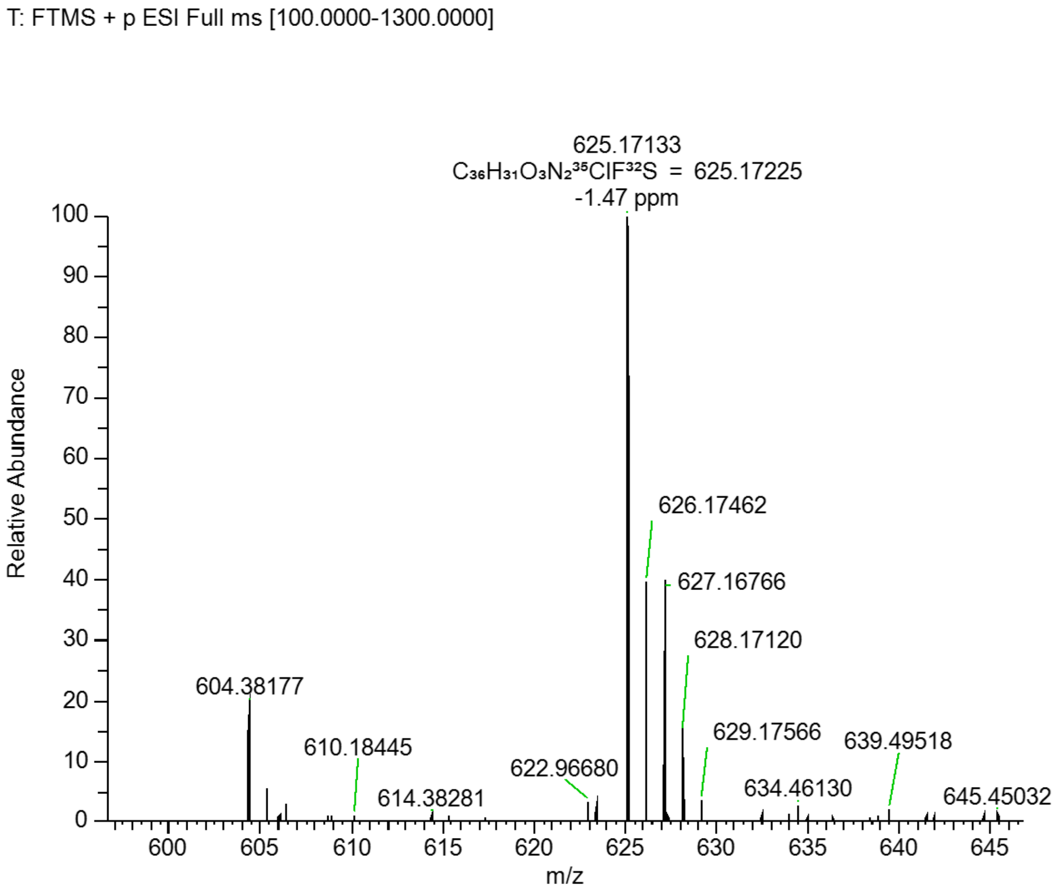

HRMS (ESI) for compound **W13**

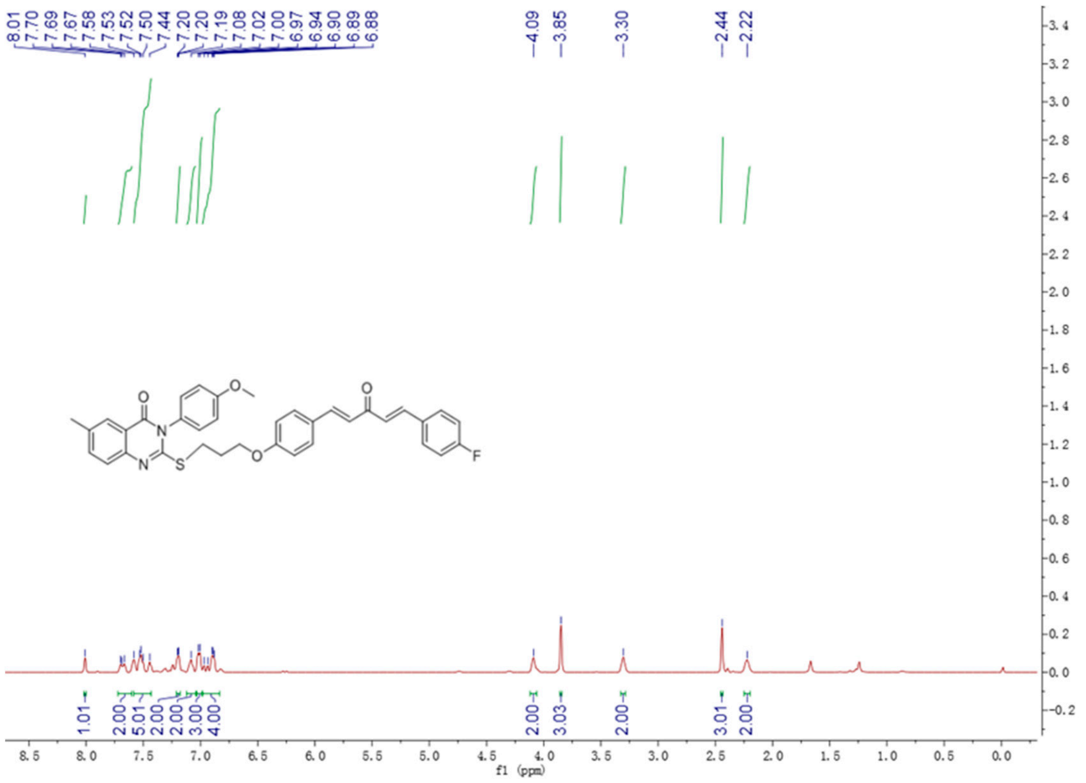

^{1}H NMR for compound **W14**

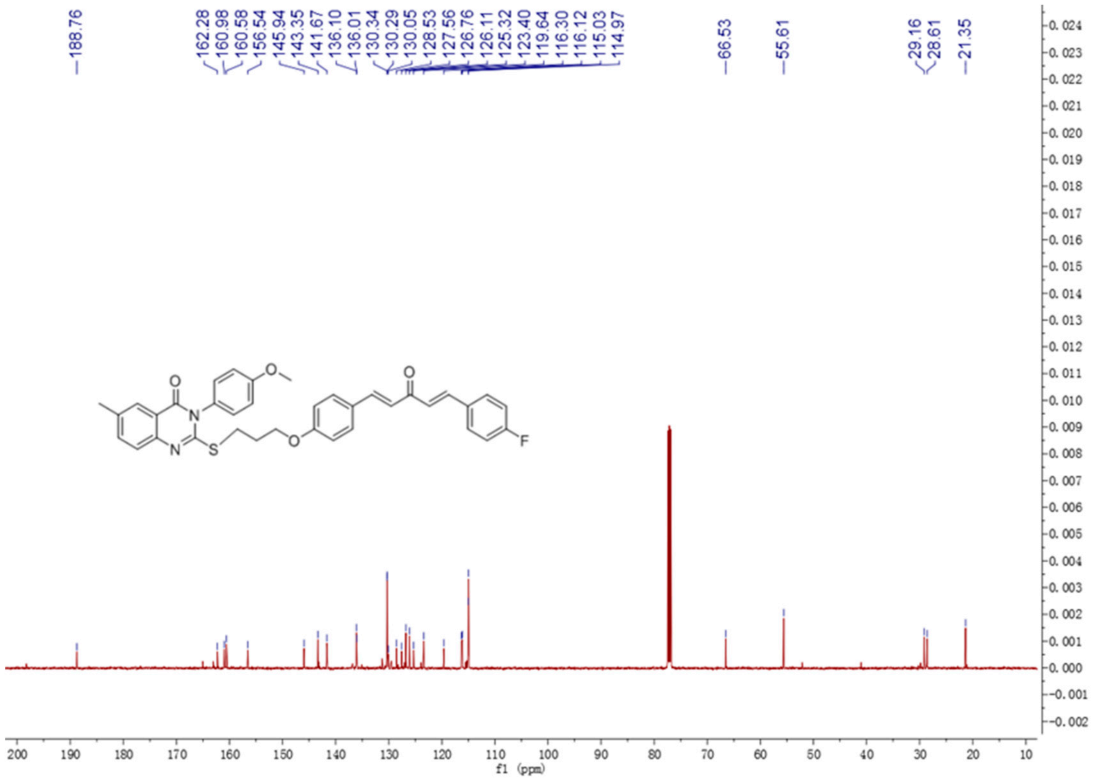

^{13}C NMR for compound **W14**

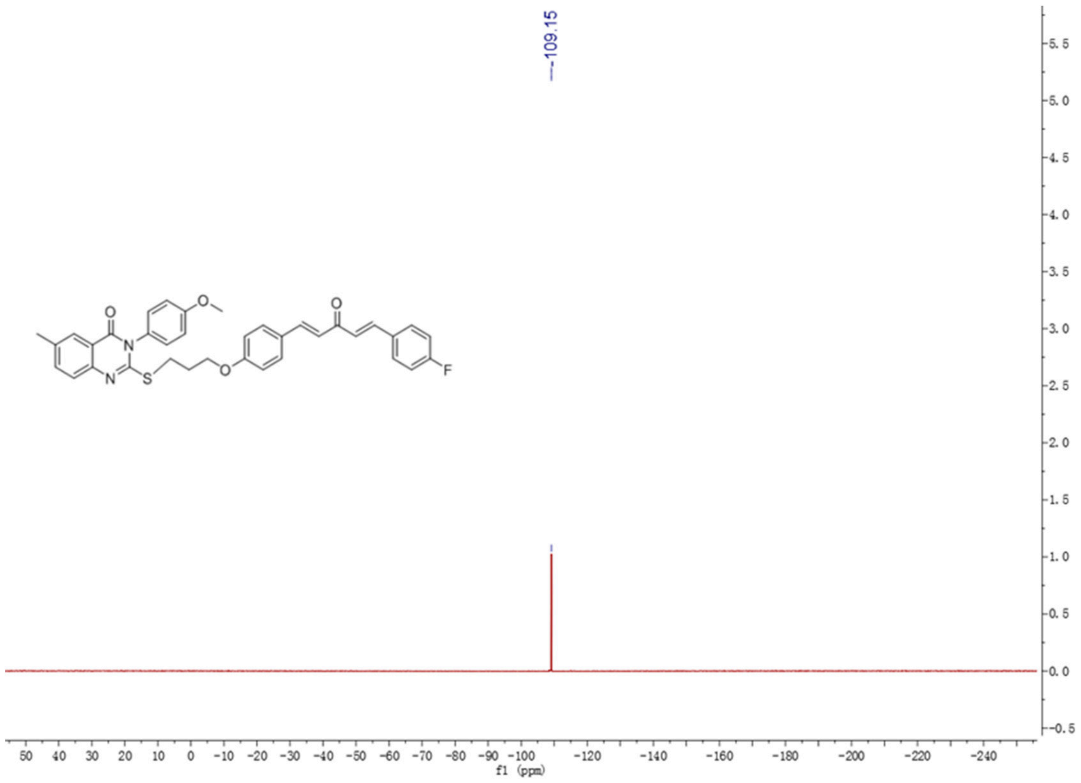

^{19}F NMR for compound **W14**

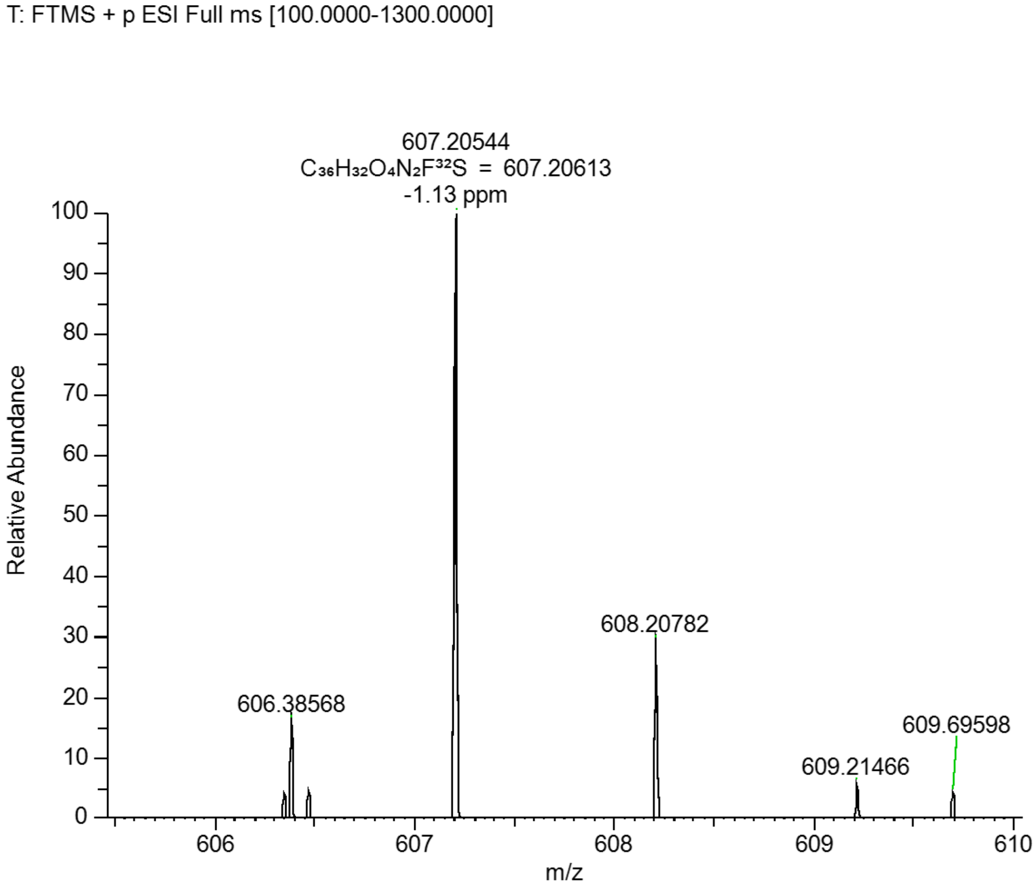

HRMS (ESI) for compound **W14**

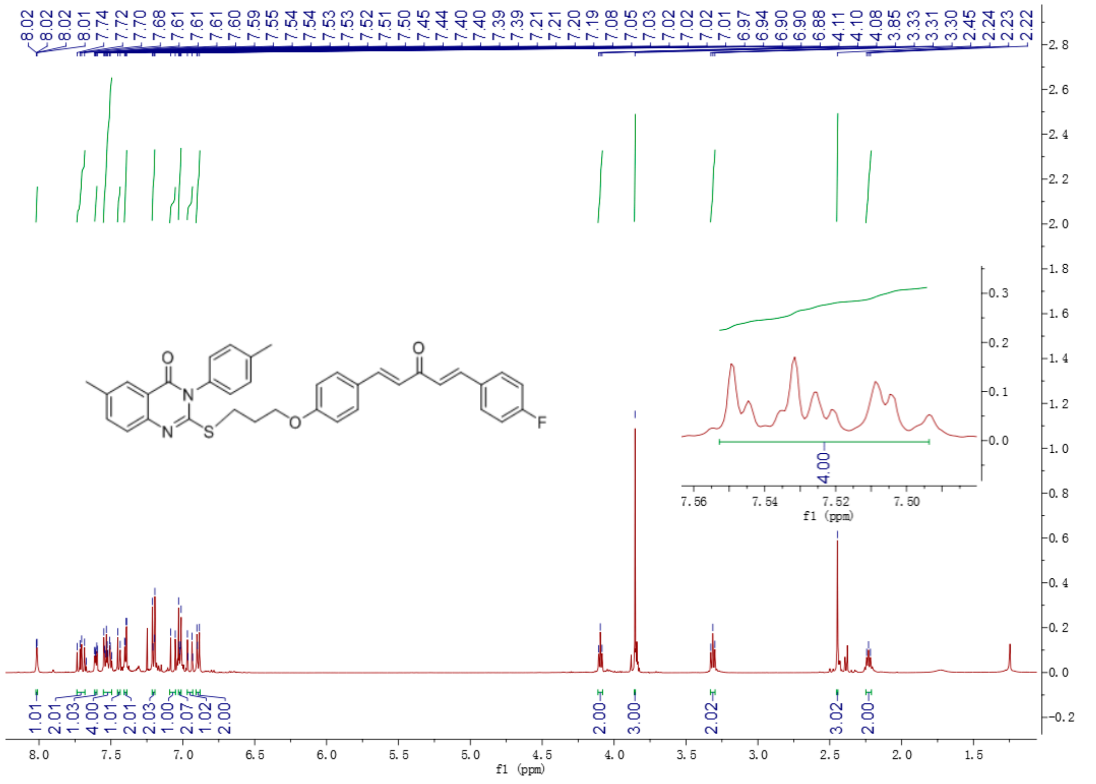

^{1}H NMR for compound **W15**

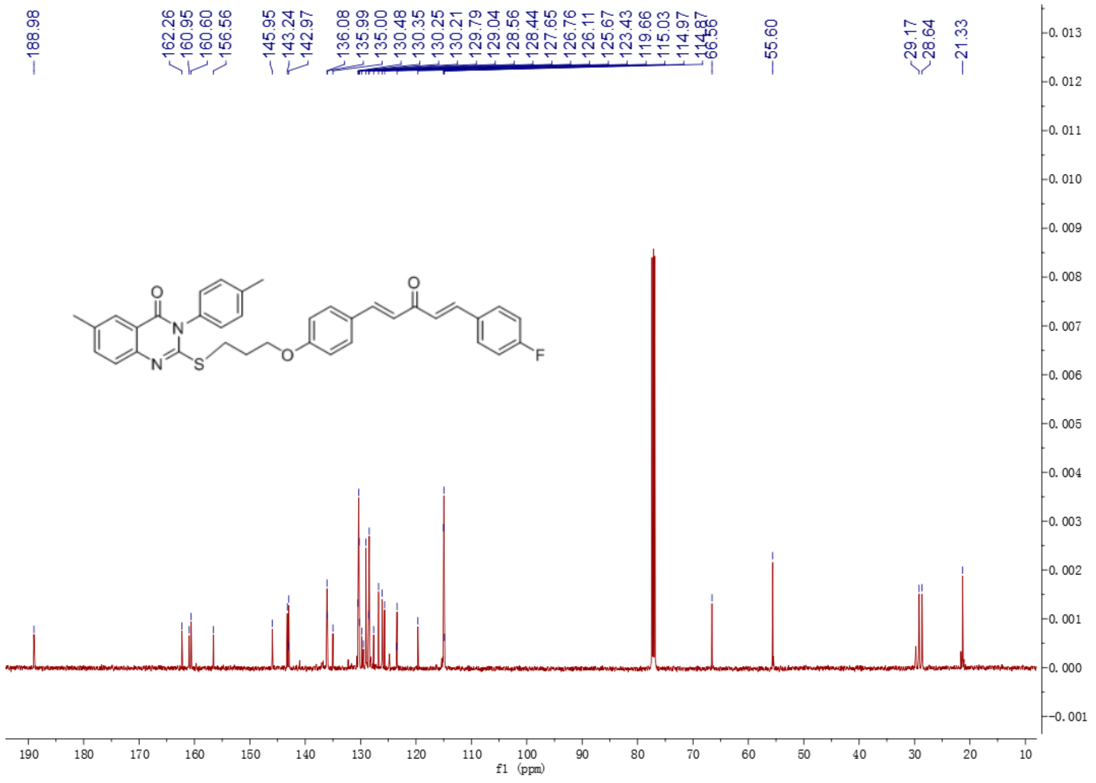

^{13}C NMR for compound **W15**

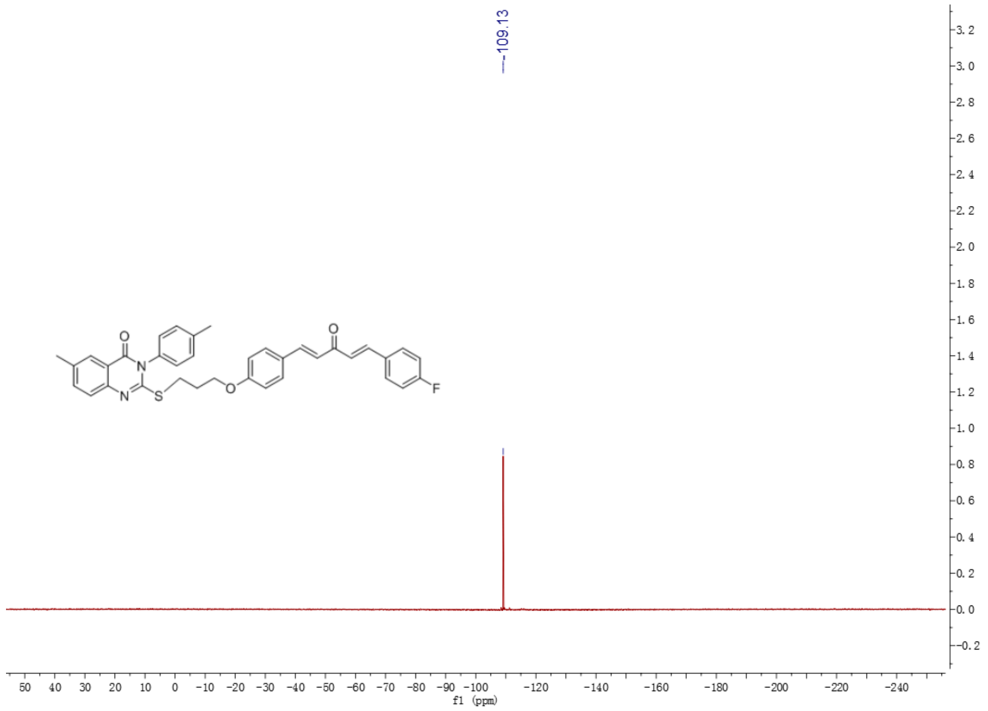

^{19}F NMR for compound **W15**

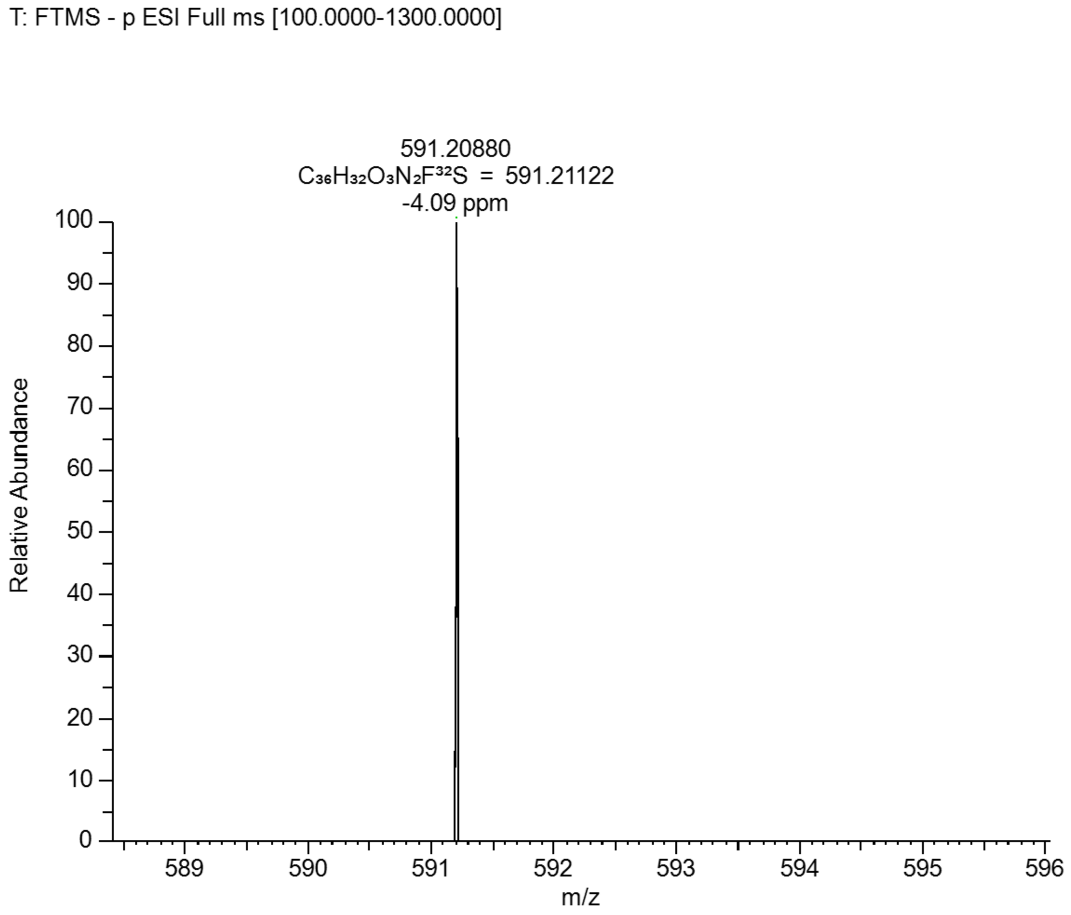

HRMS (ESI) for compound **W15**

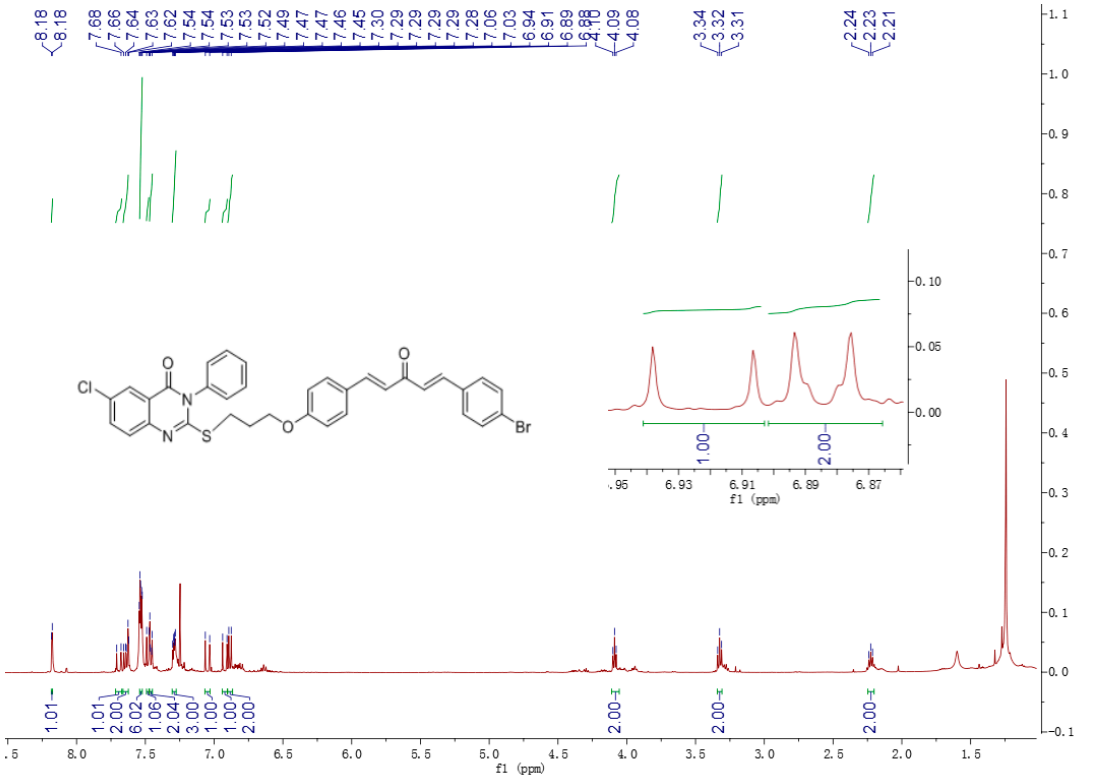

^{1}H NMR for compound **W16**

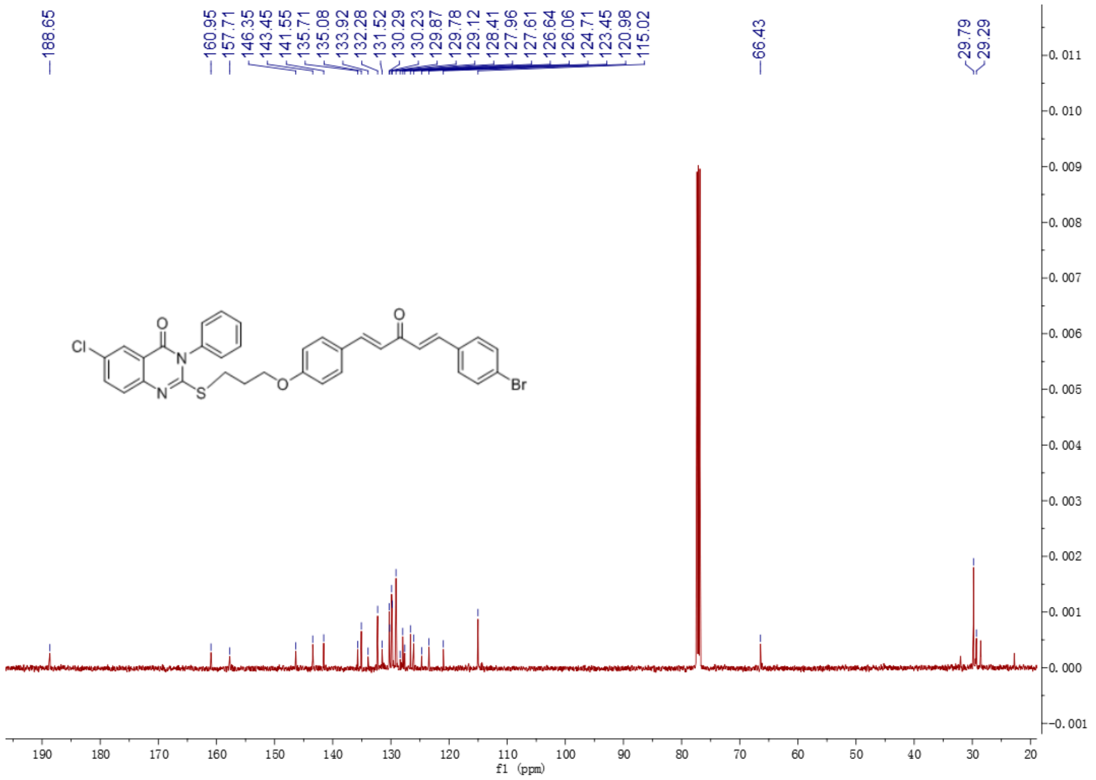

^{13}C NMR for compound **W16**

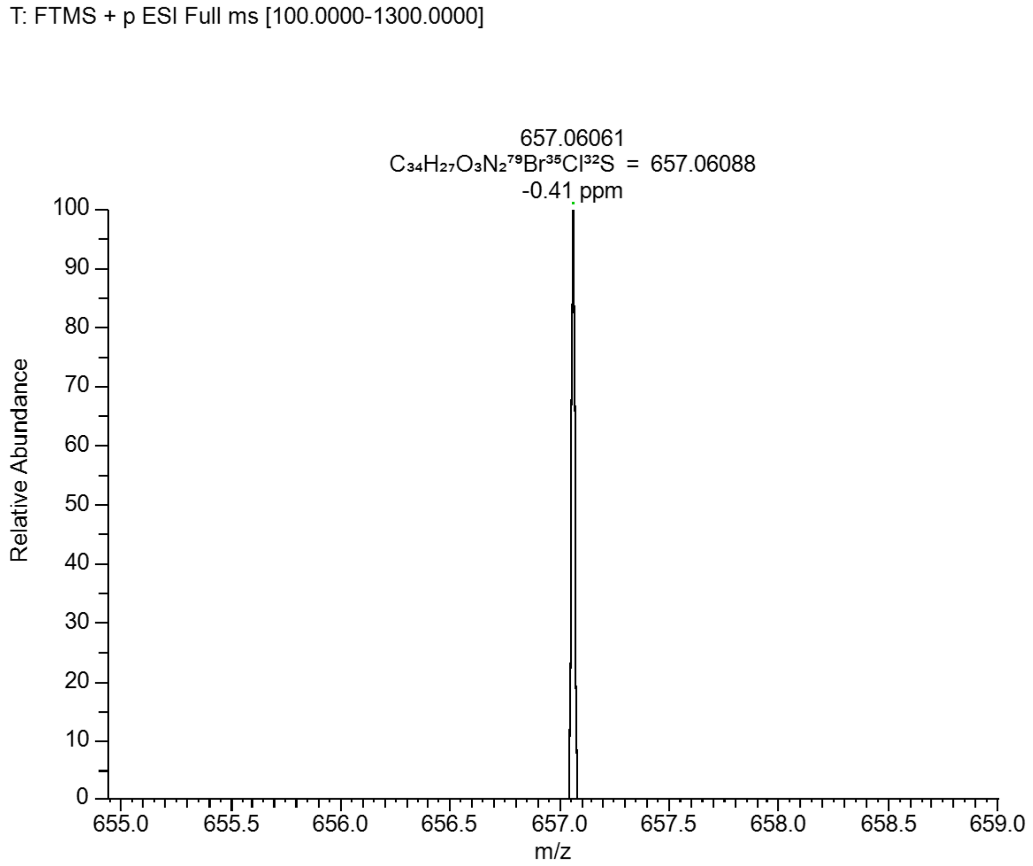

HRMS (ESI) for compound **W16**

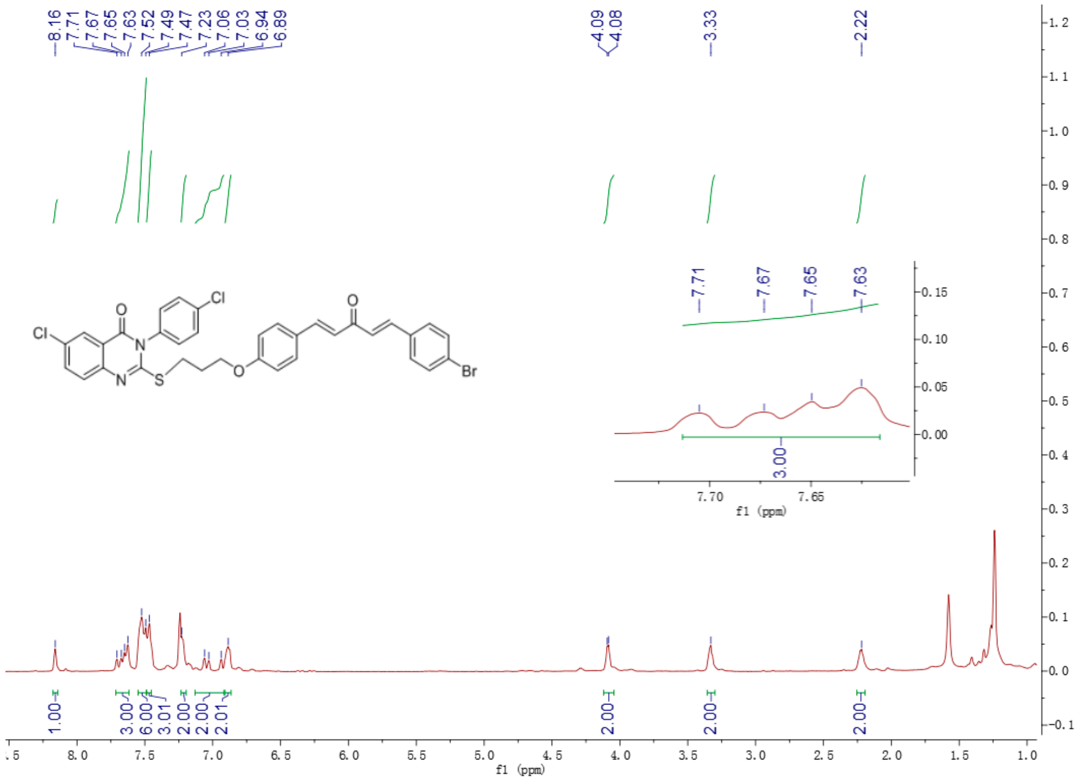

^{1}H NMR for compound **W17**

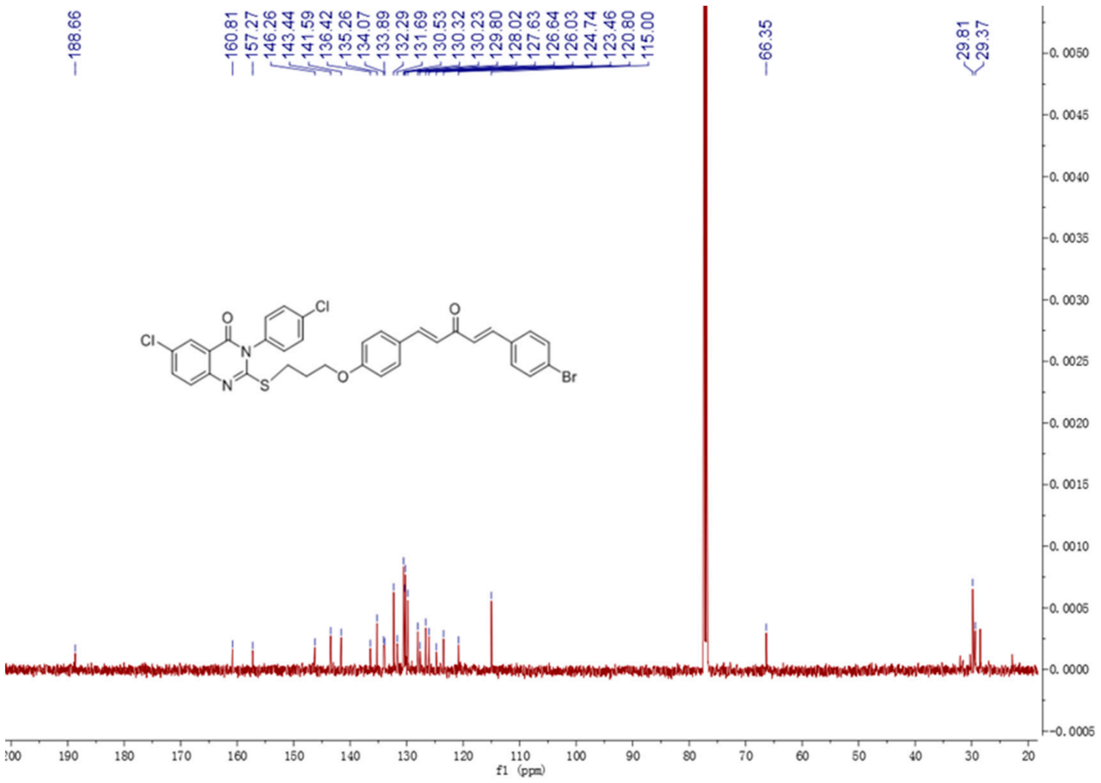

^{13}C NMR for compound **W17**

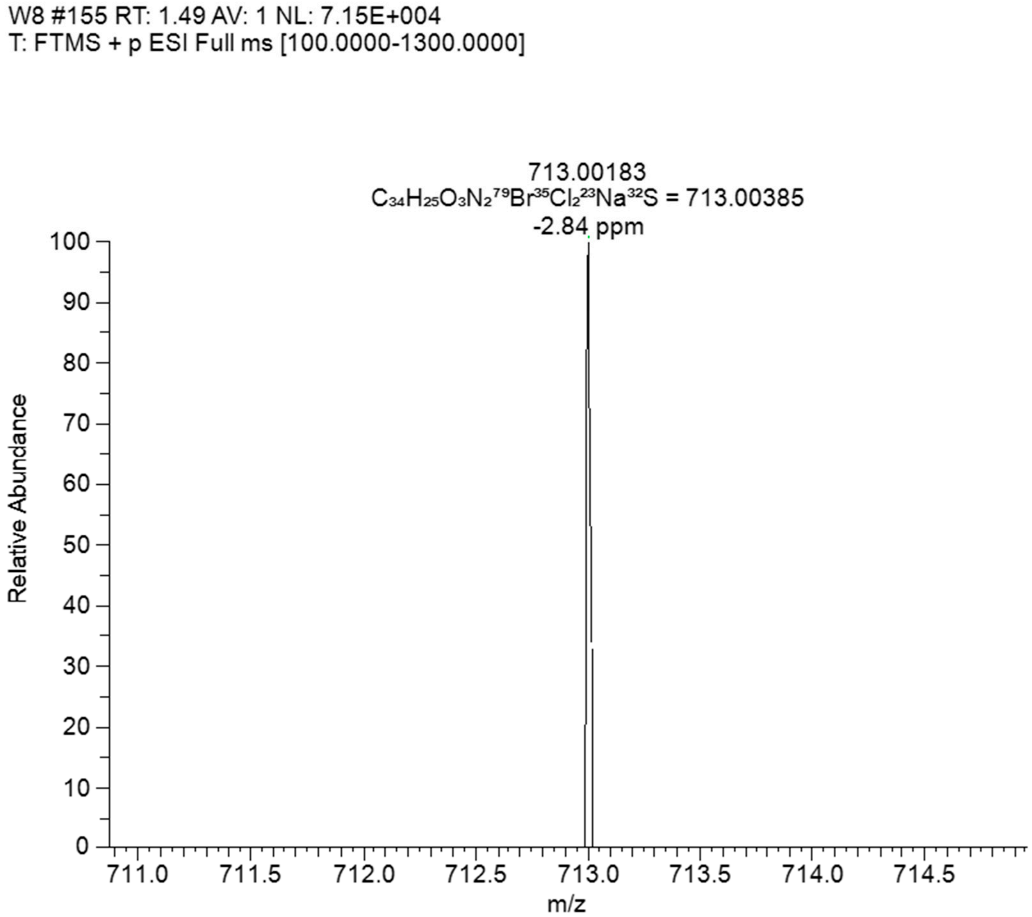

HRMS (ESI) for compound **W17**

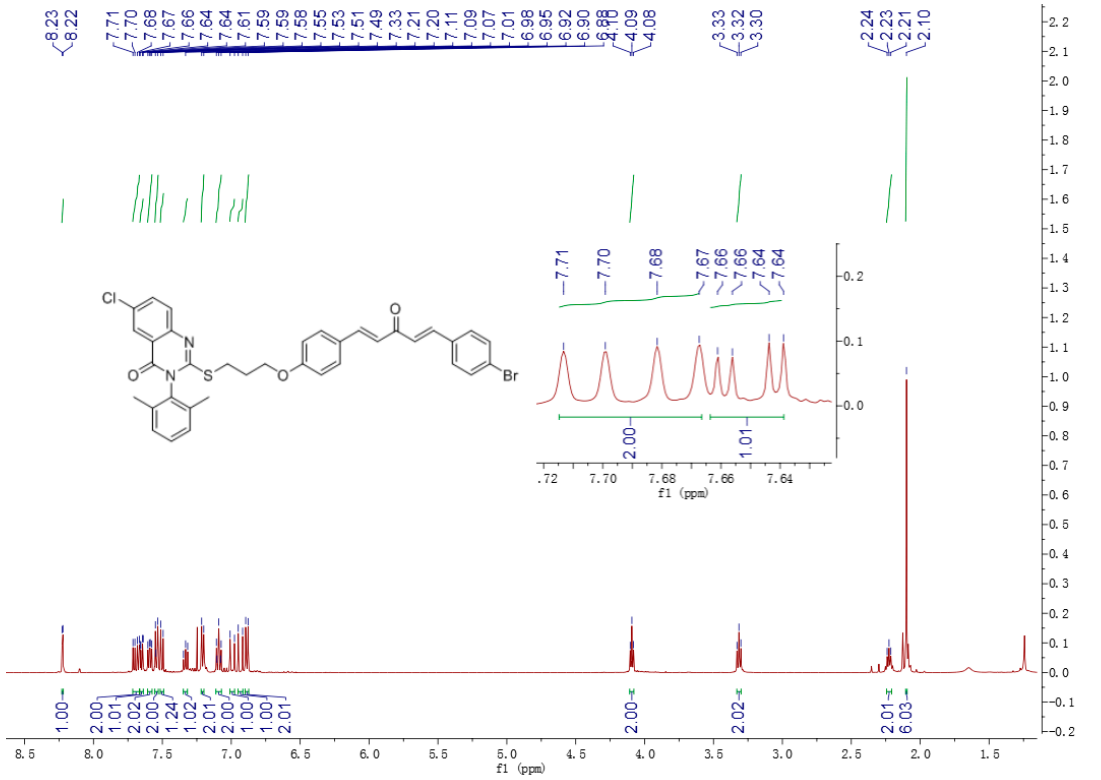

^{1}H NMR for compound **W18**

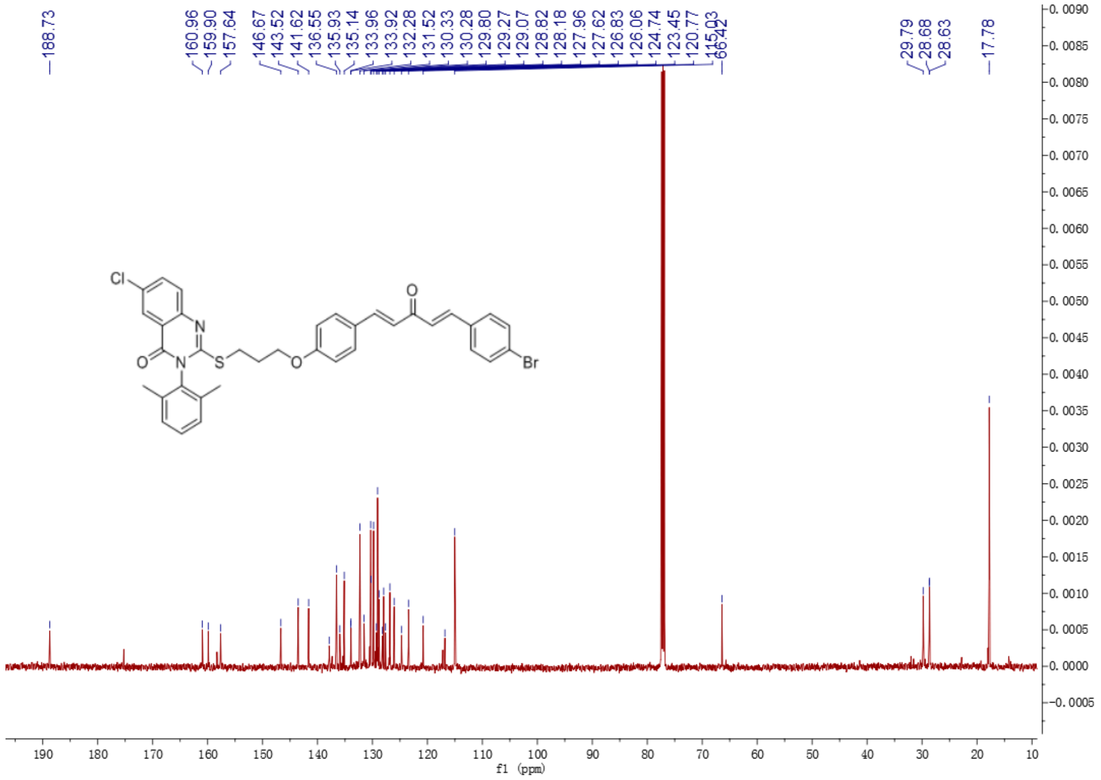

^{13}C NMR for compound **W18**

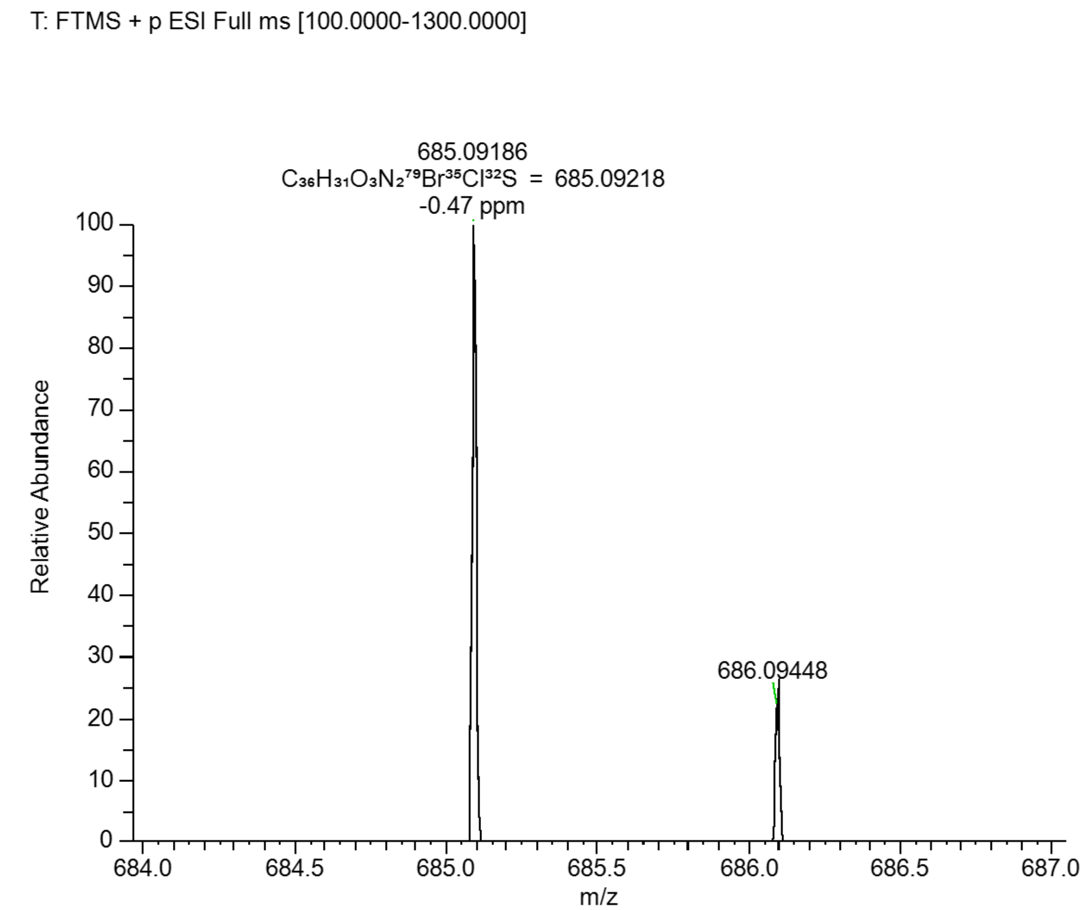

HRMS (ESI) for compound **W18**

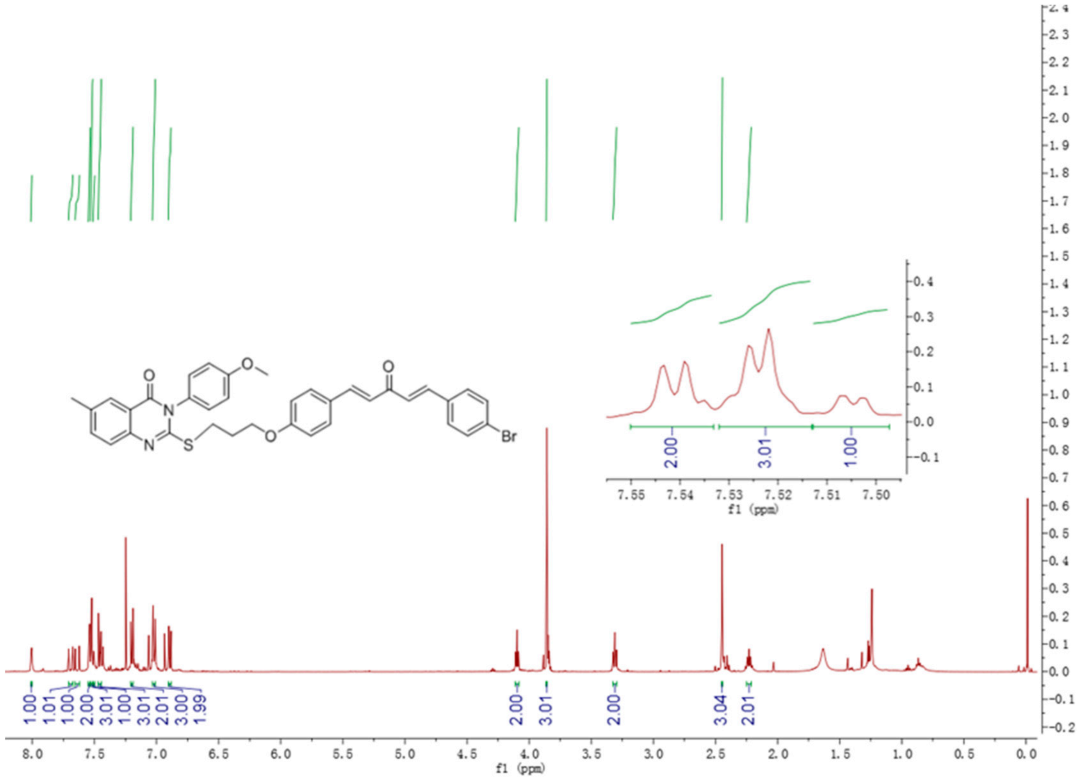

^{1}H NMR for compound **W19**

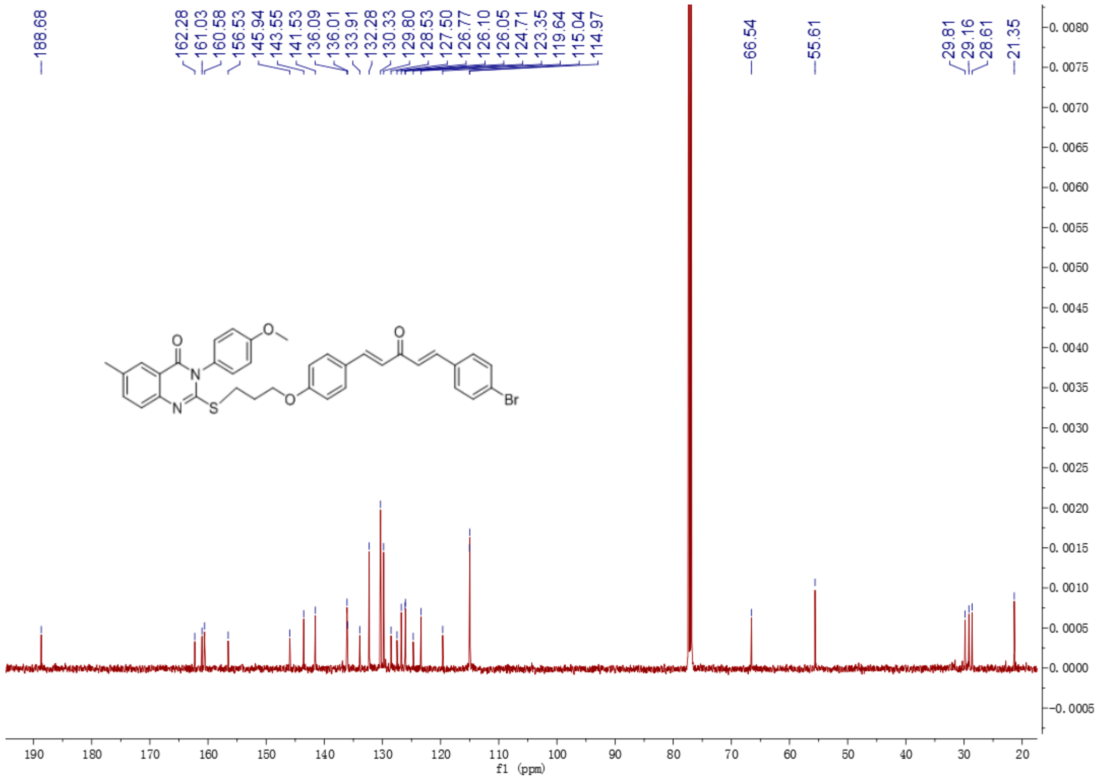

^{13}C NMR for compound **W19**

T: FTMS + p ESI Full ms [100.0000-1300.0000]

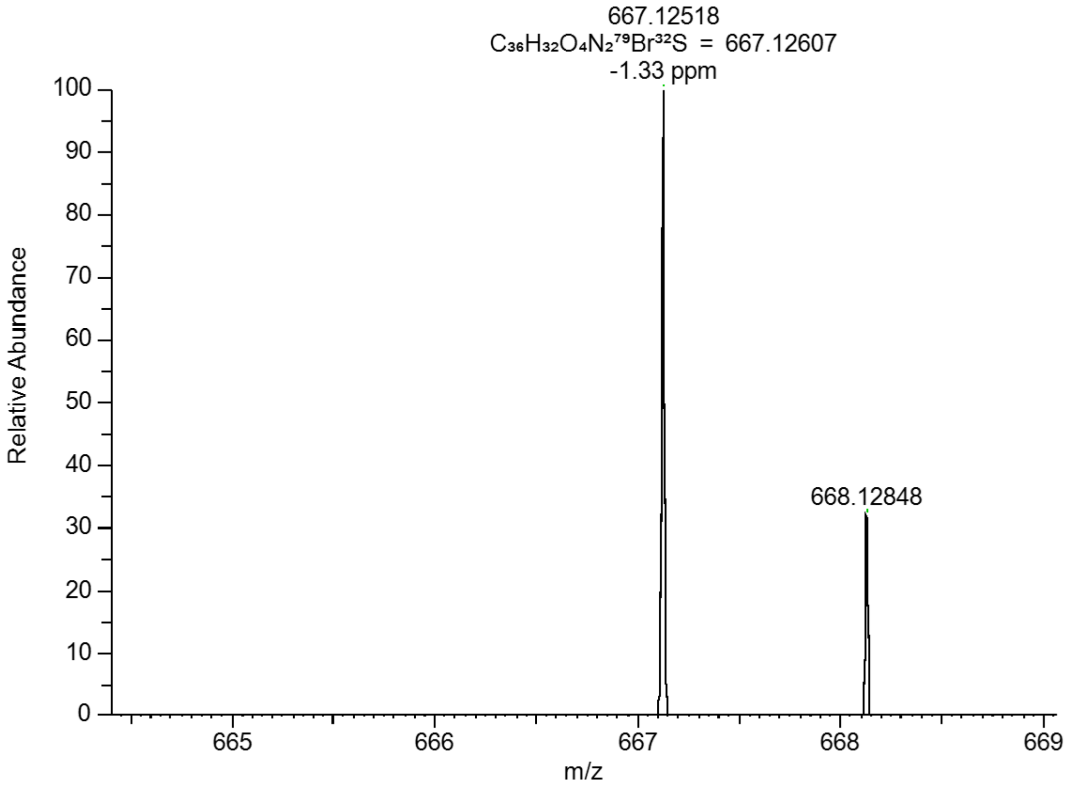

HRMS (ESI) for compound **W19**

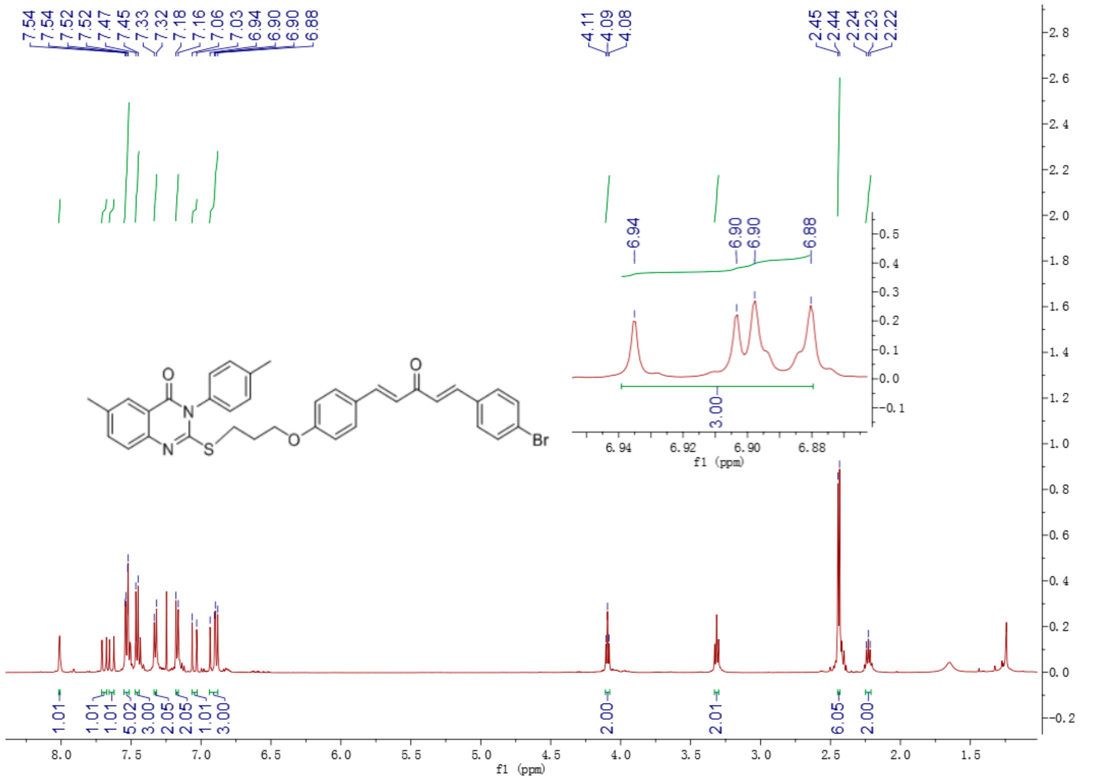

^{1}H NMR for compound **W20**

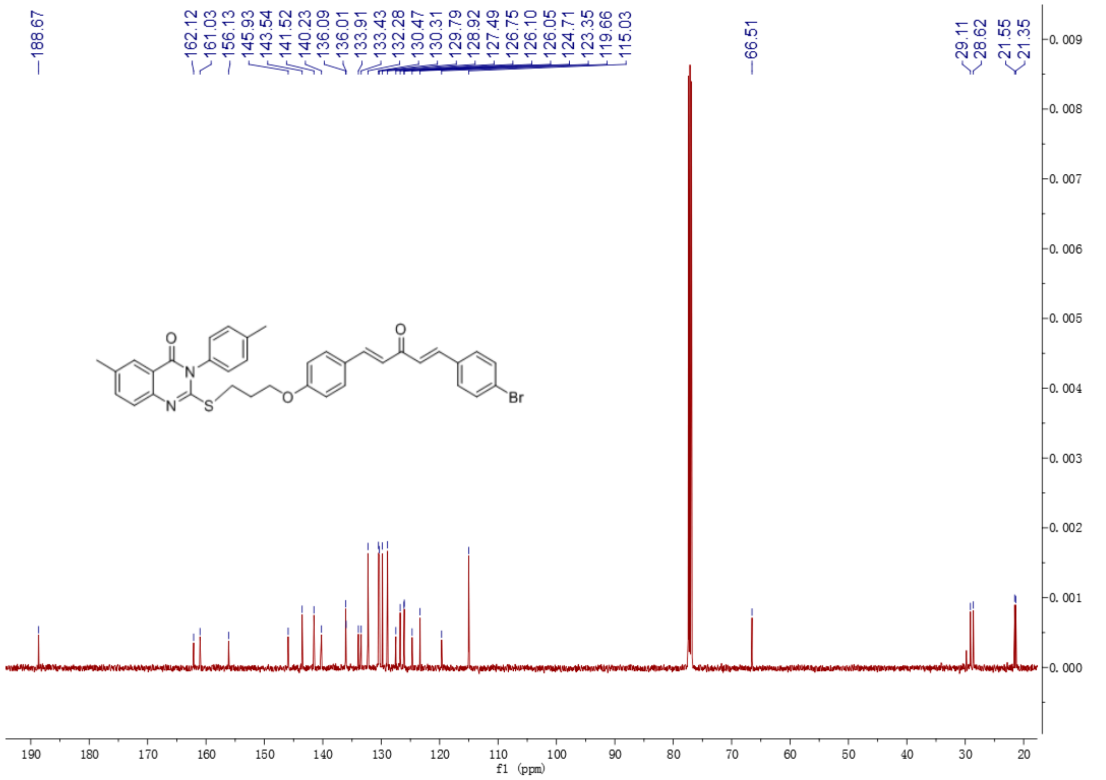

^{13}C NMR for compound **W20**

T: FTMS + p ESI Full ms [100.0000-1300.0000]

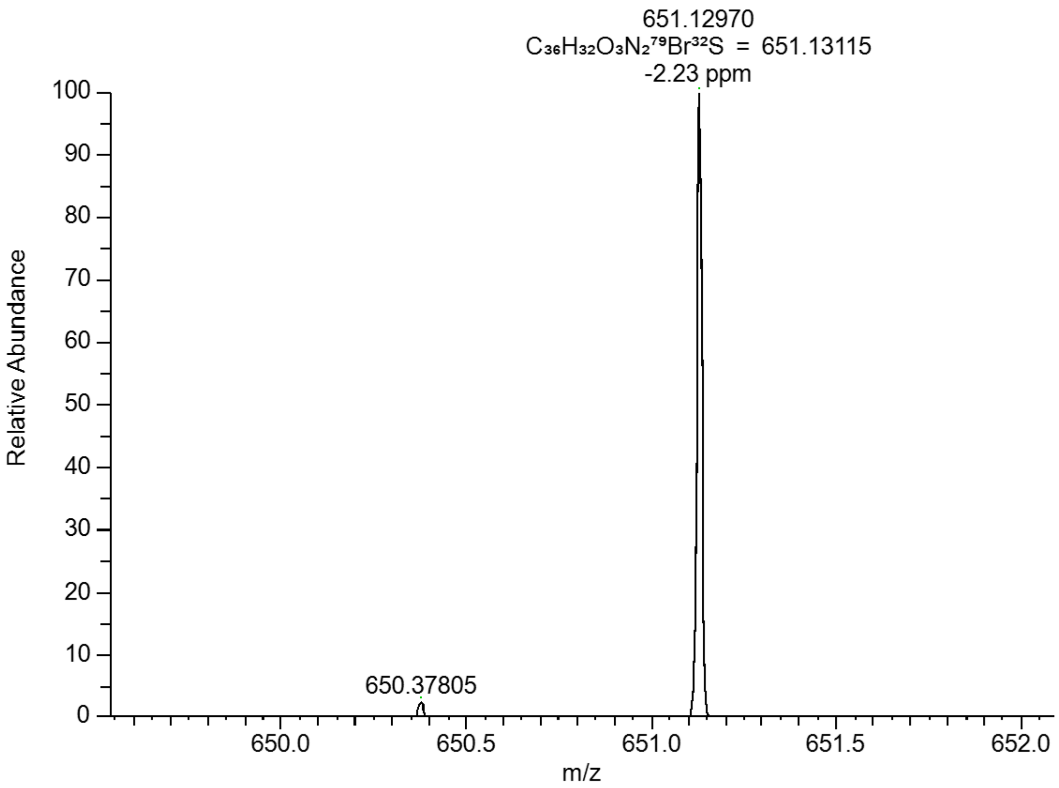

HRMS (ESI) for compound **W20**
